# A general and scalable DNA nano-chip with a fully localized architecture enables biocomputing in living cells and precisely induces cell apoptosis

**DOI:** 10.1039/d6sc01897a

**Published:** 2026-03-23

**Authors:** Jintao Yi, Tingting Chen, Jinghong Li

**Affiliations:** a Beijing Life Science Academy Beijing 102209 China jhli@mail.tsinghua.edu.cn; b State Key Laboratory of Chemo and Biosensing, College of Chemistry and Chemical Engineering, Hunan University Changsha 410082 P. R. China chenting1104@hnu.edu.cn; c Key Laboratory of Synthetic Pharmaceutical Chemistry of Jiangxi Province, Gannan Normal University Ganzhou 341000 P. R. China

## Abstract

DNA logic circuits have made important progress towards mimicking functions analogous to silicon-based electronic circuits. However, because of limitations in the orthogonality of free-floating DNA logic components and difficulty in controlling the intrinsically random collision of DNA molecules, the complexity, scalability, and information processing ability of DNA circuits are still constrained. Here, we demonstrate a general and scalable DNA nano-chip by integration of multilayer basic DNA logic gates on a DNA origami structure. We created basic DNA logic gates based on DNA localized strand displacement reactions. The basic logic gates were modularly combined into circuits by spatially arranging all of the reactive DNA components on a DNA origami structure according to the wiring instructions, establishing the generality and scalability of our DNA origami-based nano-chips. We showed that up to 11 addressable logic components were reconfigured in a single nano-chip for seven-input multi-level logic cascading and parallel biocomputing, executing highly complex tasks. We further integrated three layers of cascade logic units on the nano-chip for intracellular molecular biocomputing to execute precise identification and specific killing of tumor cells. Compared to circuits with diffusible components, our nano-chip enabled the performance of more efficient biocomputing both in solution and in living cells. Thus, we anticipate that our strategy will hold great potential for building complex DNA computing networks to perform powerful biological functions.

## Abstract 2

General and scalable nano-chips with fully localized architectures were constructed by reconfiguring basic logic gates into integrated circuits.

## Graphical abstract

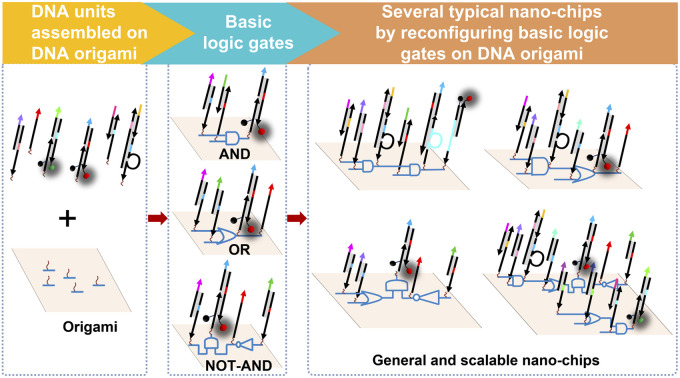

## Introduction

DNA logic circuits represent the forefront of biological computing and intelligent bionic systems. By constructing programmable DNA domino architectures driven by DNA molecular hybridization and strand displacement reactions, they precisely simulate the computational functions of electronic logic gates. These systems also exhibit remarkable modularity and scalability. Examples include Boolean logic circuits,^1^ analog neural networks,^2^ and DNA arithmetic systems,^3,4^ all enabled by the utilization of free-floating components in solution. While elegant, these integrable DNA logic systems typically rely on diffusive collisions between logic gates, resulting in slow kinetics and limited spatial controllability. Furthermore, unintentional binding interactions between free-floating DNA components could degrade the computational performance. These effects are likely to become more pronounced as the size of the circuit increases.

Several tethered circuits based on DNA origami scaffolds have been reported and take advantage of spatial constraints to overcome the limitations of slow kinetics and inefficiency.^5–7^ By harnessing the nanometer precision of DNA origami systems,^8–10^ they accomplish the precise spatial positioning of logic elements, establish a correlation between reaction rates and inter-component distances, and exhibit scalability in logic computation. However, such tethered DNA circuits are not fully localized architectures. They still require free-floating components to engage in logic operations, along with spatially localized architectures to prevent unintended signal leakage. Tethered circuits integrating both spatially localized architectures and free-floating components may pose challenges for maintaining stoichiometric balance during their delivery into living cells, potentially limiting their applicability for intracellular biocomputing. Additionally, the reliance on freely diffusing components to minimize leakage in some designs of tethered circuits could also affect the robustness of the logic operation, especially regarding the performance in complex and crowded cellular milieus.

Some nano-robot logic systems have exhibited substantial potential in intracellular biocomputing.^11–13^ They primarily focus on structural reconfiguration and the delivery of DNA components with precise stoichiometry. Limited by structural reconfiguration, these nano-robot logic systems performed relatively simple logic processing upon sensing input targets, making them difficult to scale for executing more complex biological computations. In view of the aforementioned challenges in DNA logic circuits, we thus aimed to arrange all logic elements on a single scaffold, achieving a fully localized circuit architecture. This fully localized circuit could not only preserve the generality and scalability of DNA domino circuits but also enable the co-delivery of all logic components, thereby enhancing the operational efficacy within living cells.

**Scheme 1:**
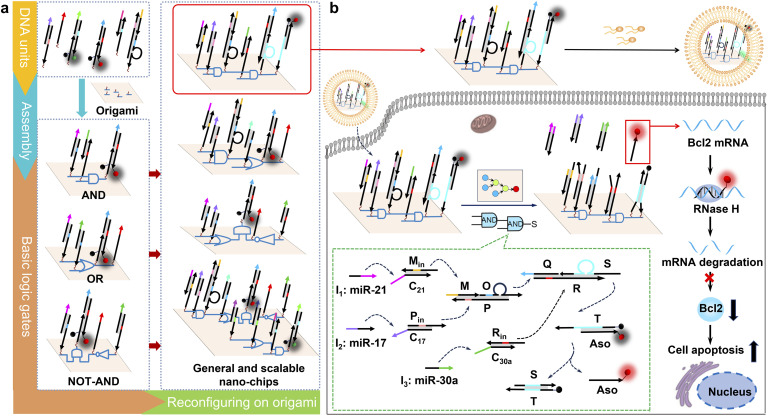
(a) Schematic illustration of the fabrication of a series of general and scalable nano-chips. Functional DNA units were assembled on a DNA origami structure to build basic DNA logic gates, including AND, OR, and NOT-AND gates. By reconfiguring basic DNA logic gates on a DNA origami structure, several typical nano-chips were constructed with multi-level logic cascades and parallel operations. (b) Schematic illustration of the nano-chip with three layers of cascade logic units to perform powerful intracellular biological functions. The nano-chip can be delivered into cells with the assistance of the transfection reagent Lipo3000. Regarding the operating mechanism of the nano-chip, briefly, two duplex DNA complexes MinC21 and PinC17 converted the input microRNAs (-21 and -17) to single-stranded DNA (ssDNA, Min, Pin), and the latter hybridized with the triple-stranded DNA complex MOP to release a ssDNA O. ssDNA Qin was the product of the hybridization reaction between input microRNA-30a and duplex DNA complex RinC30a. Subsequently, ssDNA S was released when the ssDNA O and Rin hybridized with the triple-stranded DNA complex QSR. Finally, the ssDNA S liberated ssDNA Aso from the functional reporter probe, outputting the antisense oligonucleotide of Bcl2 (Aso) to perform powerful biological functions. The segments with the same color indicate complementary sequences in the toehold-mediated strand displacement reaction. A top view of the localized circuit on the origami structure (middle, bottom) shows the three basic logic gates as color-coded circles (AND: blue; OR: yellow; NOT: brown; assistant component of the NOT gate: purple red; output S_0_: red; and output S_1_: green).

Here, we design a general and scalable DNA origami-based nano-chip with a compact structure, high efficiency and arbitrary logic. We used a toehold-mediated strand displacement reaction to construct basic logic units, including AND, OR, and NOT gates, due to its ability to take part in modular molecular design and potential to realize large-scale cascade logic computation.^14–16^ As shown in Scheme 1a, the different basic DNA logic gates can be modularly assembled on a DNA origami structure using addressable wiring instructions to form complex circuits of multi-level logic cascades and parallel operations. This modular design strategy enables spatially constrained biocomputing and directional signal transmission, establishing the generality and the scalability of our nano-chip. Furthermore, the DNA origami structure can be easily internalized by cells and remains intact in cell lysates or living cells for extended periods.^17–20^ Based on the molecular characteristics of three microRNAs (-21, -17 and -30a) that are overexpressed in HeLa cells,^21^ we reconfigured a nano-chip with three layers of cascade logic units for intracellular molecular biocomputing (Scheme 1b). In the first cascade layer, two duplex DNA complexes, M_in_C_21_ and P_in_C_17_, convert the input microRNAs (-21 and -17) into single-stranded DNA (ssDNA, M_in_, P_in_). These ssDNA strands subsequently hybridize with the triple-stranded DNA complex MOP, leading to the release of another ssDNA strand O. ssDNA R_in_ is the product of the strand displacement reaction between input microRNA-30a and duplex DNA complex R_in_C_30a_. Subsequently, ssDNA S is released when the ssDNA O and R_in_ hybridized with the triple-stranded DNA complex QSR in the second cascade logic layer. In the final stage, the third cascade layer is activated as the ssDNA S liberates the functional ssDNA Aso from the reporter probe, producing either a fluorescence signal or antisense oligonucleotides (Aso) targeting B-cell lymphoma 2 (Bcl2) for precise tumor cell identification and specific elimination. Our nano-chip demonstrates robust biological functionality within complex biological environments.

## Results and discussion

### Modular design mechanism and performance of the basic logic computing units

In the modular design mechanism, the triple-stranded DNA complex served as the key logic component in the nano-chip. This design maintained consistent forms of input and output signals, making it suitable for cascade circuits. Agarose gel electrophoresis assays confirmed that the triple-stranded DNA complex ABC could be formed by mixing three ssDNAs with equal concentrations (Fig. S1a, lane 7). Then, with the addition of two ssDNAs, A_in-1_ and B_in-1_, the ssDNA C was released from the triple-stranded DNA complex, and two new duplex DNA complexes (AA_in-1_ and BB_in-1_) emerged (Fig. S1b, lane 5), confirming the feasibility of diffusible basic logic components in biocomputing. To demonstrate that this basic biocomputing functionality could also be exhibited on the DNA origami structure, we first prepared a DNA origami structure following established methods,^20–22^ with characterization by gel electrophoresis and AFM imaging, as shown in Fig. S2. The triple-stranded DNA complex ABC was then assembled on the DNA origami structure to form an origami-based basic logic unit (Fig. S3a). As expected (shown in the truth value table in Fig. S3b), a strong fluorescence signal was observed only when both ssDNA A_in-1_ and B_in-1_ were incubated with the origami-based basic logic unit (Fig. S3c). The true/false threshold (1/0) was determined to be 1.9, calculated as the mean maximum value of false outputs plus 10 times the standard deviation (Fig. S3d). This threshold determination was consistently applied to subsequent logic operations.

### Design and performance of the basic logic gates assembled on the origami scaffold

**Fig. 1:**
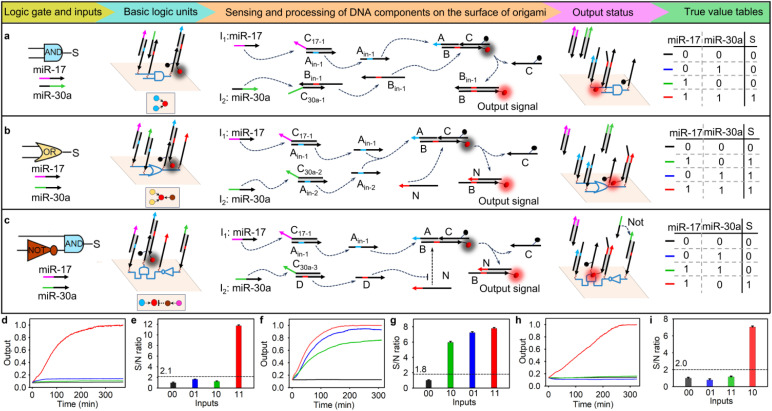
Design and performance of the basic logic gates assembled on the origami scaffold. (a) The construction of the AND logic nano-chip, an illustration of its operating mechanism and the truth value table. (b) The construction of the OR logic nano-chip, an illustration of its operating mechanism and the truth value table. (c) The construction of the AND-NOT logic nano-chip, an illustration of its operating mechanism and the truth value table. (d) Time-dependent reactivity of the AND logic nano-chip (40 nM) corresponding to different inputs (the concentration of each input miRNA was 40 nM). (e) S/N ratio obtained from (d) with the fluorescence intensity at 300 min. (f) Time-dependent reactivity of the OR logic nano-chip (40 nM) corresponding to different inputs (the concentration of each input miRNA was 40 nM). (g) S/N ratio obtained from (f) with the fluorescence intensity at 300 min. (h) Time-dependent reactivity of the AND-NOT logic nano-chip (40 nM) corresponding to different inputs (the concentration of each input miRNA was 40 nM). (i) S/N ratio obtained from (h) with the fluorescence intensity at 300 min. The top views of the localized circuits on the origami structure in (a), (b) and (c) show the three basic logic gates as color-coded circles (AND: blue; OR: yellow; NOT: brown; assistant component of the NOT gate: purple red; output: red). The segments with the same color are complementary sequences of the toehold domain. The error bars indicate mean ± standard deviation (SD, *n* = 5). The true/false (1/0) threshold values for (a), (b) and (c) were obtained by calculating the mean maximum value of false outputs plus 10 times the standard deviation to reduce misjudgements at the boundary. More detailed descriptions of the wiring instructions are given in Fig. S41.

Different basic logic gates were constructed based on the above origami-based logic computing unit. We first designed a two-input AND gate. As shown in Fig. 1a, two duplex DNA complexes—A_in-1_C_17-1_ and B_in-1_C_30a-1_—and a triple-stranded DNA complex, ABC, were spatially arranged by wiring instruction to form an origami-based AND gate. In the AND logic operation, two duplex DNA complexes, A_in-1_C_17-1_ and B_in-1_C_30a-1_, converted the input microRNAs (-17 and -30a) into single-stranded DNA (A_in-1_, B_in-1_), and the latter hybridized with the triple-stranded DNA complex ABC to release ssDNA C and produce a new duplex DNA complex BB_in-1_, resulting in a fluorescence signal. We used agarose gel electrophoresis assays to confirm the orthogonal performance of the diffusible logic components. When two duplex complexes, A_in-1_C_17-1_ and B_in-1_C_30a-1_, were incubated with the triple-stranded complex ABC, distinct bands were observed (Fig. S4, lane 5). After introducing two input microRNAs, a ssDNA C was obtained (Fig. S4, lane 2). In contrast, no ssDNA C emerged when only one input microRNA was incubated with triple-stranded DNA complex ABC (Fig. S4, lanes 3 and 4). These results implied that two input miRNAs could activate the corresponding toehold-mediated strand displacement.

Considering that the distance-dependent hybridization efficiency directly impacts the signal propagation on the nano-chip, we first optimized the assembled distance between the input and output DNA components on the DNA origami structure. It was found that when the assembled distance was 12 nm on the AND logic nano-chip, the maximum signal-to-noise (S/N) ratio could be obtained (Fig. S5). Subsequently, we performed AND biocomputing in the nano-chip. As expected (shown in the truth value table in Fig. 1a), when both miRNAs were present (1,1), a strong fluorescence signal was obtained with the true/false (1/0) threshold calculated as 2.1. The fluorescence intensity nearly reached its maximum after 240 min of incubation (Fig. 1d and e). In comparison, the diffusible AND biocomputing system at the same concentration recovered only 60% of the maximum signal achieved by the nano-chip, even after incubation with two miRNAs for 350 min (Fig. S6). The data were subsequently analyzed using a first-order kinetic model to estimate the kinetic rate constants for these two systems. The kinetic rate constants were 0.0013 min^−1^ and 0.0303 min^−1^ for the diffusible system and nano-chip, respectively. These results indicated that spatially localized logic components on the nano-chip enable faster computation and higher orthogonality than the diffusible system, which could be attributed to their elevated local concentration and directional information transmission.

In the operation of the two-input OR gate, one or both input miRNAs displaced ssDNA A_in-1_ or A_in-2_ from its respective duplex DNA complex (A_in-1_C_17-1_ or A_in-2_C_30a-2_). The released ssDNA strands then hybridized with the triple-stranded DNA complex ABC, leading to the formation of duplex DNA complex BC. Subsequently, ssDNA N released ssDNA C and produced duplex DNA complex BN (Fig. 1b). The agarose gel electrophoresis assays showed that the presence of either miRNA-17 or miRNA-30a could directly result in the hybridization between strand B and strand N, releasing ssDNA C (Fig. S7, lanes 2, 3, and 4). In the OR logic nano-chip, the maximum signal-to-noise (S/N) ratio could be obtained when the assembly distance between the input and output DNA components was 12 nm (Fig. S8). Experimentally, negligible output fluorescence was observed in the absence of both inputs. However, the addition of either one or both inputs resulted in significant output fluorescence signals (Fig. 1f), and the true/false (1/0) threshold was calculated to be 1.8 (Fig. 1g).

As NOT gates inherently begin generating output signals before receiving input information,^23^ their experimental implementation presents significant challenges. To implement a NOT gate in biocomputing, we constructed a two-input logic cascade circuit in which a logical “NOT” fed into a logical “AND”, to avoid a false output. As shown in Fig. 1c, the input miRNA-17 displaced ssDNA A_in-1_ from duplex DNA complex A_in-1_C_17-1_, and the latter hybridized with the triple-stranded DNA complex ABC to release duplex DNA complex BC. In the absence of miRNA-30a, ssDNA N released ssDNA C and produced duplex DNA complex BN, resulting in a truth fluorescence signal output. Once in the presence of miRNA-30a, duplex DNA complex DC_30a-3_ converted it into the ssDNA D, which could preferentially hybridize with ssDNA N to prevent subsequent displacement reaction. The agarose gel electrophoresis assays verified that hybridization between strands B and N, with consequent release of ssDNA C, occurred exclusively in the presence of miRNA-17 and absence of miRNA-30a (Fig. S9, lane 3). The optimal assembly distance between the input and output DNA components remained at 12 nm in the AND-NOT logic nano-chip (Fig. S10). The fluorescence experiments also demonstrated that the cascade nanochip generated a high output signal only under these specific conditions (Fig. 1h), and the true/false (1/0) threshold was calculated to be 2.0 (Fig. 1i).

To demonstrate the generality of the design strategy, we constructed other basic logic nano-chips using two microRNAs (-122a, -15a) as inputs, including AND, OR, and NOT-AND gates. As shown in Fig. S11–S16, upon reprogramming a duplex DNA complex for different logic operations, the diffusible basic logic components exhibited the expected orthogonality, and the nano-chips correctly generated TRUE outputs. Notably, the triple-stranded DNA complex ABC, as the core component for the final signal output, could be shared across all two-input logic gates, while the same assistant strand N was also used in both OR and NOT gates for their respective logic operation. Additionally, 5-nucleotide (nt) toehold-mediated strand displacement reactions were utilized in all logic circuits for the input identification, information transmission and signal outputting, due to their rapid displacement and minimized leakage in the DNA domino circuits.^23^ The above results and the modular design mechanism illustrated that our nano-chips had a fully localized architecture through spatial arrangement of the modular logic units on DNA origami structures, and had the potential to achieve fast and arbitrary logic cascades.

### Complex DNA cascade circuits with fully localized architectures

To demonstrate the feasibility of constructing DNA cascade circuits with fully localized architectures through modular recombination of logic units on DNA origami structures, we built a series of different multi-input DNA cascade circuits. On the basis of the basic AND nano-chip, we constructed a two-layered AND-OR circuit, in which the output of the first AND gate was relayed to the input of the second OR gate (Fig. S17a). Using three miRNAs (-122a, -15a and -17) as inputs and testing with specific combinations [(001), (110), (101), (011), (111)], the nano-chip showed high output fluorescence (Fig. S17b and c) and the true/false (1/0) threshold was calculated to be 2.1 (Fig. S17d). The experimental data showed the expected Boolean logic from our design strategy. Next, another nano-chip with a cascade circuit was constructed by cascading AND-NOT-AND logic. Specifically, we replaced the second OR in the above three-input cascade circuit with a NOT-AND gate, in which the output of the first AND gate and the output of the NOT gate were relayed to the input of the final AND logic (Fig. S18a). When all miRNAs except miRNA-30a were introduced [inputs (110)], there was high output fluorescence (Fig. S18b and S18c), which was consistent with the design of this three-input AND logic, and the true/false (1/0) threshold was calculated to be 2.5 (Fig. S18d).

Next, on the basis of the basic OR nano-chip, we constructed a complex OR-NOT-AND cascade circuit on the DNA origami structure. By replacing the first AND logic in the above OR-NOT-AND nano-chip with a logical OR (Fig. S19a), a strong fluorescence signal was obtained after the addition of either one or both of the input miRNA-122a and miRNA-15, simultaneously without miRNA-30a (Fig. S19c). The experimental data were consistent with the design of OR-NOT-AND logic nano-chips and the true/false (1/0) threshold was calculated to be 2.1 (Fig. S19d). We also built a four-input AND gate by cascading a pair of two-input OR gates, in which two outputs of the OR gate were relayed to the inputs of the final AND logic (Fig. S20a). Using four miRNAs (-122a, -124a, -30a and -15a) as inputs, when any of the following combinations of the miRNAs [(1010), (1001), (0110), (0101), (0011), (1110), (1101), (0111), (1011) and (1111)] were introduced to the OR-OR-AND logic nano-chip, enhanced signals were observed (Fig. S20b and c), and the true/false (1/0) threshold was calculated to be 2.0 (Fig. S20d).

**Fig. 2:**
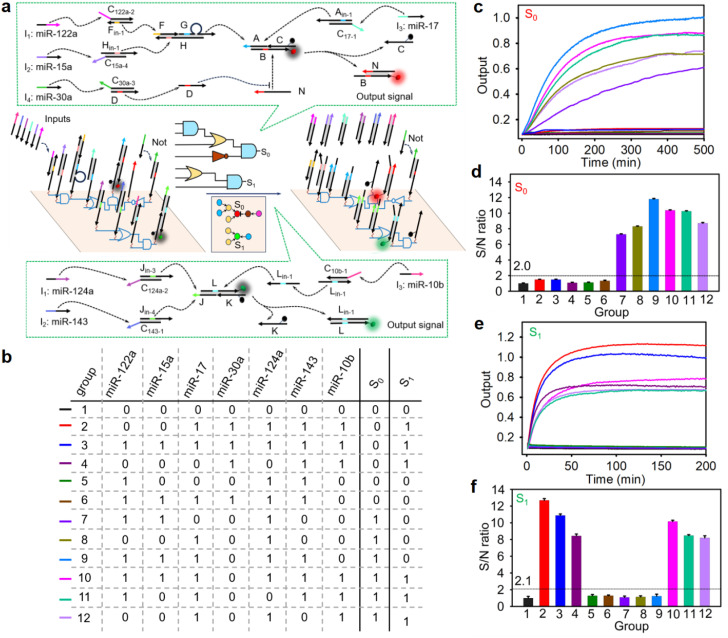
Design and performance of the scalable nano-chip with 11 addressable logic components for seven-input multi-level logic cascading and parallel biocomputing. (a) An illustration of the nano-chip operating mechanism. In brief, in the AND-OR-NOT-AND circuit, two duplex DNA complexes F_in-1_C_122a-2_ and H_in-1_C_15a-4_ converted the input microRNAs (-122a and -15a) to single-stranded DNA (ssDNA, F_in-1_, H_in-1_), and the latter hybridized with the triple-stranded DNA complex FGH to release ssDNA G. Input miR-17 displaced ssDNA A_in-1_ from duplex DNA complex A_in-1_C_17-1_. One of the ssDNA G or ssDNA A_in-1_ hybridized with the triple-stranded DNA complex ABC to release the duplex DNA complex BC. Then, ssDNA N liberated ssDNA C and produced duplex DNA complex BN, resulting in the fluorescence signal S_0_. Once in the presence of miR-30a, duplex DNA complex DC_30a-3_ converted it to ssDNA D, which could preferentially hybridize with ssDNA N to prevent subsequent displacement reaction. In the OR-AND circuit, one or both input miRNAs displaced ssDNA J_in-3_ or J_in-4_ from duplex DNA complex J_in-3_C_124a-2_ or J_in-4_C_143-1_, and the latter hybridized with the triple-stranded DNA complex JKL to release duplex DNA complex KL. Input miR-10b displaced ssDNA L_in-1_ from duplex DNA complex L_in-1_C_10b-1_. Then, ssDNA L_in-1_ liberated ssDNA K and produced duplex DNA complex LL_in-1_, resulting in the fluorescence signal S_1_. The top view of the localized circuit on the origami structure shows the three basic logic gates as color-coded circles (AND: blue; OR: yellow; NOT: brown; assistant component of the NOT gate: purple red; output S0: red; output S1: green). The segments with the same color are complementary sequences of the toehold domain. More detailed descriptions of the wiring instructions are given in Fig. S44a. (b) The truth value table of the parallel nano-chip; (c) time-dependent reactivity of the AND-OR-NOT-AND circuit corresponding to different inputs; (d) the S/N ratio obtained from (c) with the fluorescence intensity at 500 min; (e) time-dependent reactivity of the OR-AND circuit corresponding to different inputs; and (f) the S/N ratio obtained from (e) with the fluorescence intensity at 200 min. The error bars indicate mean ± SD (*n* = 5).

To further demonstrate the generality and scalability of our nano-chip, we positioned two distinct multi-input circuits side by side on a single DNA origami structure in accordance with the wiring instructions (called dual-parallel integrated circuits, Fig. 2a), and triggered them simultaneously to determine whether two circuits on the same origami structure could biocompute different input groups concurrently. As expected (see the truth value table in Fig. 2b), one four-input circuit (AND-OR-NOT-AND circuit) exported fluorescence signal S_0_ (Cy5 signal) after responding to the corresponding input combinations of four miRNAs [miR-122a, -15a, -17 and -30a were combined into input groups as follows: (1100), (0010), (1110), (1010)], even when there were different combinations of interfering miRNAs (miR-124a, -143, -10b, input-groups 7–12, Fig. 2c). The true/false (1/0) threshold of the S_0_ rail circuit was calculated to be 2.0 (Fig. 2d). Another three-input circuit (OR-AND circuit) exported fluorescence signal S_1_ (FAM signal) when three miRNAs (miR-124a, -143, -10b) were combined into input groups as follows: (111), (011), (101), even when there were different combinations of interfering miRNAs (miR-122a, -15a, -17 and -30a, input-groups 2–4, 10–12, Fig. 2e). The true/false (1/0) threshold of the S1 rail circuit was calculated to be 2.1 (Fig. 2f). In addition, the circuits consisting of the same discrete logic components in the solution at concentrations identical to the corresponding nano-chip exhibited a lower kinetic rate constant and inefficient biocomputing (Fig. S21 and S22). These results indicated that the integrated circuits with a fully localized architecture on the DNA origami structure not only accelerated the reaction kinetics but also reduced crosstalk between orthogonal gates. Moreover, in a typical nano-chip loading with an AND-OR-NOT-AND circuit, some essential DNA components (strand N or triple-stranded DNA complex FGH) that determine the directional flow were not assembled onto the DNA origami structure, resulting in the failure of signal transduction after response to the correct inputs (Fig. S23). This result demonstrated that our DNA nano-chip carried out biocomputing in a directional transmission manner.

To further illustrate the intelligence of our nano-chip in biological information processing, a cross-selective nano-chip with dual-parallel integrated circuits on a DNA origami structure was constructed. As shown in Fig. S24a, miR-122a and miR-17 were the common inputs to the dual-parallel integrated circuits and miR-30a and -10b, as the selected signal inputs, which determined the final outputs. As expected (see the truth value table in Fig. S24b), the dual-parallel integrated circuits exhibited enhanced fluorescence signals S_0_ (Cy5 signal) and S_1_ (FAM signal) (Fig. S24c–f), after responding to the corresponding input combinations of miRNAs [miR-122a, -15a, -17, and -30a were combined into input groups as follows: (0010), (1100), (1010), (1110) for the S_0_ signal, and miR-122a, -17 and -10b were combined into input groups as follows: (101), (111), (011) for the S_1_ signal]. The data also reflected that there was no crosstalk signal in the two parallel biocomputing systems (Fig. S24d: group 3, 5, 6, 8, and 13–16; and Fig. S24f: group 3, 5, 6, and 8–16), demonstrating good applicability for complex information processing.

### Intracellular biocomputing based on the nano-chip

**Fig. 3:**
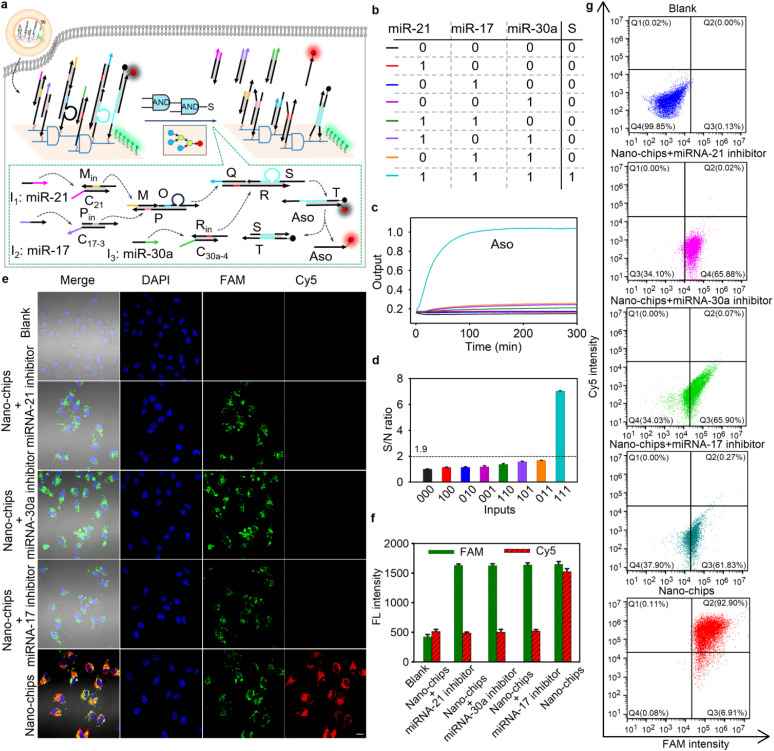
The design and performance of the nano-chip with three layers of cascade logic units. (a) An illustration of the nano-chip operating mechanism in living cells. More detailed descriptions of the wiring instructions are given in Fig. S45. (b) The truth value table of the nano-chip; (c) time-dependent reactivity of the nano-chip corresponding to different inputs; (d) S/N ratio obtained from (c) with the fluorescence intensity at 300 min; (e) CLSM images of HeLa cells after different treatments (scale bar: 20 µm); (f) relevant statistical histogram analysis of the CLSM images; and (g) the corresponding flow cytometry analysis for HeLa cells after different treatments.

Based on the molecular characteristics of three microRNAs (-21, -17 and -30a) overexpressed in HeLa cells,^24^ a nano-chip with a three-input AND cascade logic circuit was designed for intracellular molecular biological computing. As shown in Fig. 3a, when all three miRNAs were present, the circuit produced output Cy5-labeled ssDNA Aso. The logic operation was verified by agarose gel electrophoresis assays (Fig. S25). To further confirm the feasibility of the three-input logic operation, fluorescence recovery assays were carried out in a homogeneous solution. As expected (see the truth value table in Fig. 3b), when all three input-miRNAs were added, the circuit exhibited an enhanced fluorescence signal. Conversely, the circuit produced a low output for any combination of two or fewer inputs (Fig. 3c). Additionally, the true/false (1/0) threshold was calculated to be 1.9 (Fig. 3d). All these results indicated that the performance of the three-input nano-chip was consistent with the pre-designed logic operation.

Previous studies had shown that DNA origami structures could remain intact in complex biological environments.^17–19^ To maintain the integrity of the nano-chip, all oligonucleotides of logic components assembled on DNA origami structures were modified with phosphorothioate bonds to resist nuclease degradation. Under different biological conditions, including culture medium (containing 10% FBS) and cell lysates, the bands of the nano-chip remained clear (Fig. S26a) and over 80% of the payload on the origami structure remained largely intact (Fig. S26b), even after incubation at 37 °C for 60 h, demonstrating the stability of our nano-chip in complex biological environments. Furthermore, the nano-chip exhibited only marginal toxicity to the HEK293 cells, even at a concentration as high as 200 nM, and the cell viability remained over 95% after 48 h of incubation (Fig. S27), indicating the good biocompatibility of our nano-chip. By labeling the DNA origami structure with fluorescein dye (FAM), we investigated the intracellular distribution of the nano-chip. As shown in Fig. S28, the green fluorescence obtained from the nano-chip overlapped slightly with the red fluorescence exhibited by commercial Lyso@tracker, implying the efficient escape of our nano-chip from the lysosome into the cytoplasm, which benefits intracellular biocomputing by using miRNAs.

Next, we investigated the feasibility of using the nano-chip with three layers of cascade logic units for three miRNA pattern imaging and AND gate operation in living cells. To verify the sensing specificity of the nano-chip, we chose HEK293 cells (human embryonic kidney cells) as the intracellular biocomputing model due to simultaneous relatively low expression levels of miRNA-21, miRNA-17 and miRNA-30a (Fig. S29), but provision of a complex biological environment. As shown in Fig. S27a, confocal laser scanning microscopy (CLSM) images showed bright green fluorescence in the cells, which was obtained from the FAM-labeled DNA origami scaffold, indicating that the nano-chip could be easily internalized by cells with the assistance of a commercial transfection reagent. When the DNA analogues of three miRNAs were all pre-delivered into HEK293 cells, obvious red fluorescence in the cells was exhibited after incubation with the nano-chip (Fig. S30a and b). Flow cytometry also verified that the nano-chip could output a signal after cell pretreatment with three DNA analogues of miRNAs (Fig. S30c). In contrast, no obvious fluorescence signal could be observed when one of the DNA analogues was not delivered into the cells (Fig. S31). These results demonstrated the ability of the nano-chip to perform powerful information processing in a complex biological environment.

Subsequently, we demonstrated the operation of the nano-chip in HeLa cells. Fig. 3e and f show the fluorescence imaging of HeLa cells and the relevant intensity statistical results after different treatments. The CLSM images show bright red fluorescence in HeLa cells after incubation with the nano-chip. In contrast, the cells pre-treated with different miRNA inhibitors showed hardly any red fluorescence signal, suggesting that the absence of one of the miRNA inputs resulted in the fluorescence signal output not being activated. Furthermore, the results of the flow cytometry analysis were consistent with the CLSM images (Fig. 3g). These results were also in good agreement with the RT-qPCR detection of relative miRNA expression levels in HeLa cells with different treatments (Fig. S32), suggesting the accuracy and reliability of our nano-chip in cell biocomputing. Moreover, to demonstrate that the integrity of the structure in the nano-chip was critical for maintaining its bio-function, three control nano-chips, in which one of the three duplex DNA complexes used to target miRNAs was not assembled on the DNA origami structure, were built. The CLSM images and relevant intensity statistical results showed that for HeLa cells treated with the control nano-chips, a negligible red fluorescence signal was observed (Fig. S33a and b). The flow cytometry result was consistent with that of the fluorescence imaging of HeLa cells with different treatments (Fig. S33c). The above results indicated that the intracellular bio-functional activity of the nano-chip could be realized only when all DNA logic units were spatially arranged on the DNA origami structure based on the wiring instructions.

Next, we compared the efficiency of the nano-chip and diffusible DNA integrated circuit in intracellular biocomputing. The nano-chip and the DNA logic units of three layers of cascade circuit with the same concentration (40 nM) were delivered into HeLa cells. As shown in Fig. S34, significant red fluorescence was obtained in the HeLa cells after treatment with the nano-chip. Meanwhile, HeLa cells incubated with the discrete logic components only displayed a weak fluorescence signal, indicating inefficient biocomputing due to their intrinsically random collisions in the complex cell environment. These results implied that the nano-chip with a fully localized structure had an advantage in intracellular biocomputing and executed precise identification in the cell with specific molecular features.

### Specific gene-silencing and cell apoptosis induced by the nano-chip

**Fig. 4:**
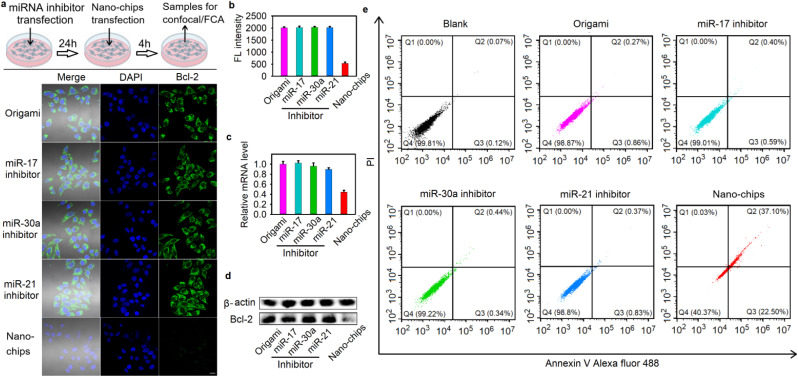
Specific gene-silencing and cell apoptosis induced by nano-chip. (a) Immuno-fluorescence imaging of Bcl2 protein in HeLa cells after different treatments, scale bar: 20 µm. The inhibitors were introduced first, followed by incubation with the nano-chip after 24 hours. (b) The relevant statistical histogram analysis of the Immuno-fluorescence imaging. (c) The relative expression levels of Bcl2 mRNA in HeLa cells after different treatments. (d) Western blotting assay of Bcl2 protein from cell lysate from HeLa cells after different treatments. (e) Flow cytometry assay for analyzing apoptosis in HeLa cells after different treatments by using Annexin V and the PI dead cell apoptosis kit.

Having demonstrated the accuracy of the nano-chip in intracellular biocomputing, we investigated its bio-function for the specific killing of tumor cells, which could be realized by outputting the antisense oligonucleotide of Bcl2 (Aso-Bcl2), because silencing of Bcl2 is an effective approach to promote cell apoptosis.^25^ We evaluated the gene-silencing efficiency of Bcl2 in HeLa cells after different treatments by cellular immunofluorescence experiments, quantitative reverse transcriptase polymerase chain reaction (qRT-PCR) and western blot assays. Compared with the blank cells without any treatment, the relative protein and mRNA expression levels of Bcl2 were downregulated after the HeLa cells were incubated with the nano-chip for 48 h. However, when the cells were pre-treated with any one of three miRNA inhibitors, no obvious fluctuation of protein and mRNA expression levels of Bcl2 were observed (Fig. 4a–d). In addition, we also investigated the effect of the nano-chip on Bcl2 expression levels in HEK293 cells. As shown in Fig. S35, the HEK293 cells treated with the nano-chip, even when two target DNA analogues were pre-delivered into the cells, showed no obvious gene silencing compared to blank HEK293 cells. These results confirmed that the nano-chip could be used for specific gene silencing efficiently.

After the silencing of Bcl2 expression was demonstrated, we next validated the specific cell apoptosis induced by the nano-chip in HeLa cells. The cells were incubated with the nano-chip for 48 h and then stained with Annexin V Alexa Fluor 488 and PI dye for confocal imaging to observe the apoptotic cells. The images showed extremely bright green fluorescence on the membranes and significant red fluorescence in the nuclei of almost all of the cells treated with nano-chip (Fig. S36a), indicating that substantial apoptosis was induced, while the blank cells and the cells pre-treated with any one of three miRNA inhibitors showed lower green and red fluorescence. We quantified the average fluorescence intensity from the images, and the results showed that the cells only treated with the nano-chip had the highest green and red fluorescence intensity (Fig. S36b), which implied a large amount of cell apoptosis induced by the nano-chip. We also carried out the flow cytometry assay of Annexin V/PI-stained apoptotic cells after different treatments with HeLa cells, and the apoptotic efficiency of HeLa cells induced by the nano-chip was 59.6% (calculated as *Q*_2_% + *Q*_3_%, all Annexin V-positive cells, Fig. 4e). In contrast, there was no apoptosis in HEK293 cells after incubation with the nano-chip, even when two target DNA analogues were pre-delivered into the cells (Fig. S37). All these results implied that the nano-chip could perform powerful biofunctions in cells with specific molecular features, such as specific gene-silencing and cell apoptosis.

## Conclusions

In this study, we successfully designed a general and scalable DNA nano-chip with modular logic units on a DNA origami structure.

The modular logic units, including AND, OR, and NOT gates, were constructed based on DNA strand displacement reactions and spatially arranged on a DNA origami structure according to wiring instructions. By recombining modular logic units on DNA origami structures, a series of multiple-input DNA nano-chips could perform information processing based on the physical localization of elements and directional transmission of signals, thereby establishing the generality and scalability of our nano-chip for biocomputing. We demonstrated that reconfiguration of up to 11 addressable logic components on a single nano-chip for seven-input multilayer logic cascading and parallel biocomputing could result in faster information processing compared to integrated circuits with discrete logic components. Moreover, based on the molecular characteristics of three microRNAs overexpressed in HeLa cells, as a paradigm, we further integrated three layers of cascade logic units on the nano-chip for intracellular molecular biocomputing. The results reflected that our three-input nano-chip could be used for the precise identification and specific killing of tumor cells. Compared to intracellular circuits with diffusible components, this nano-chip also enabled the performance of more efficient biocomputing in living cells. While our current intracellular demonstration focused on a simple three-input AND gate, and more complex circuits incorporating NOT gates had yet to be applied in cellular biocomputing (an important direction in future studies), the nano-chip loaded with dual-parallel integrated circuits we constructed nevertheless demonstrated significant capability. It enabled seven-input multi-level logic cascades and parallel operations in a homogeneous solution, responding to sensing inputs derived from intracellular miRNAs. Due to its inherent generality and scalability, our nano-chip paves the way for the development of sophisticated DNA computation networks in living cells, which would be capable of executing precise, multi-functional biological programs, with particular promise for applications in precision diagnostics and targeted therapy.

## Author contributions

Jintao Yi: conceptualization, data curation, formal analysis; Tingting Chen: investigation, writing – original draft, writing – review & editing, funding acquisition; Jinghong Li: supervision, project administration, writing – review & editing, funding acquisition.

## Conflicts of interest

There are no conflicts to declare.

## Supplementary Material

SC-017-D6SC01897A-s001

## Data Availability

The data supporting this article have been included as part of the supplementary information (SI). Supplementary information: experimental procedures, agarose gel electrophoresis, characterization, fluorescence measurements, CLSM images, wiring instructions of the logic gates on the DNA origami structure, and sequence of oligonucleotides used in this work. See DOI: https://doi.org/10.1039/d6sc01897a.
