# Supplementary material for: A general and scalable DNA nano-chip with a fully localized architecture enables biocomputing in living cells and precisely induces cell apoptosis
Source: Chem Sci. 2026 Mar 23;17(19):9674–83. doi: 10.1039/d6sc01897a (PMC13036796; doi:10.1039/d6sc01897a)
Supplement: SC-017-D6SC01897A-s001 [file SC-017-D6SC01897A-s001.pdf]

Supplementary Information (SI) for Chemical Science.
This journal is © The Royal Society of Chemistry 2026

Supporting Information

A General and Scalable DNA Nano-chip with Fully Localized Architecture Enables Biocomputing in Living cells and Precisely Induces Cell Apoptosis

Jintao Yi, Tingting Chen* and Jinghong Li*

Table of Contents

Experimental Procedures......S3
Figure S1. Agarose gel electrophoresis analysis of the comple ABC and its reaction with ssDNA......S7
Figure S2. Characterization of DNA origami......S 8
Figure S3. Reaction performance of basic DNA units (the triple-stranded DNA complex ABC) on DNA origami......S9
Figure S4. Agarose gel electrophoresis analysis of the toehold mediated strand displacement reaction in AND logic design......S10
Figure S5. The optimizing the assembly distance between input and output DNA components on the AND logic nano-chip......S11
Figure S6. Time-dependent Cy5 fluorescence signals of the AND gate in nano-chip or discrete DNA circuit response to different inputs......S12
Figure S7. Agarose gel electrophoresis analysis of the toehold mediated strand displacement reaction in OR logic design......S13
Figure S8. The optimizing the assembly distance between input and output DNA components on the AND logic nano-chip......S14
Figure S9. Agarose gel electrophoresis analysis of the toehold mediated strand displacement reaction in AND-NOT logic design......S15
Figure S10. The optimizing the assembly distance between input and output DNA components on the AND-NOT logic nano-chip......S16
Figure S11. Design and performance of the general AND logic nano-chip using two microRNAs (-122a, -15a) as inputs......S17
Figure S12. Agarose gel electrophoresis analysis in general AND logic nano-chip using two microRNAs (-122a, -15a) as inputs......S18
Figure S13. Design and performance of the general OR logic nano-chip using two microRNAs (-122a, -15a) as inputs......S19
Figure S14. Agarose gel electrophoresis analysis in general OR logic nano-chip using two microRNAs (-122a, -15a) as inputs......S20
Figure S15. Design and performance of the general NOT-AND logic nano-chip using two microRNAs (-122a, -15a) as inputs......S21
Figure S16. Agarose gel electrophoresis analysis in general AND-NOT logic nano-chip using two microRNAs (-122a, -15a) as inputs......S22
Figure S17. Design and performance of the AND-OR logic nano-chip using three microRNAs (-122a, -15a and -17) as inputs......S23
Figure S18. Design and performance of the AND-NOT-AND logic nano-chip using three microRNAs (-122a, -15a and -30a) as inputs......S24
Figure S19. Design and performance of the OR-NOT-AND logic nano-chip using three microRNAs (-122a, -15a and -30a) as inputs......S25
Figure S20. Design and performance of the OR-OR-AND logic nano-chip using three microRNAs (-122a, -124a, -15a and -30a) as inputs......S26
Figure S21. Time-dependent Cy5 fluorescence signal of the S_0 circuit in nano-chip or discrete DNA circuit response to different inputs......S27
Figure S22. Time-dependent FAM fluorescence signal of the S_1 circuit in nano-chip or discrete DNA circuit response to different inputs......S28
Figure S23. The performance of different nano-chip in responding to inputs (miR-122a, miR-15, miR-17)......S29
Figure S24. Design and performance of the scalable cross-selective nano-chip......S 30
Figure S25. Agarose gel electrophoresis analysis in three-inputs AND nano-chip using two microRNAs (-21, -17 and -30a) as inputs......S31
Figure S26. Study on the stability of the nano-chip......S32
Figure S27. Cell viability of HEK293 cells after incubated with different concentrations of nano-chip......S33
Figure S28. Endo-lysosomal release of nano-chip in HeLa cells......S34
Figure S29. The relative expression levels of miRNAs in different cells......S35
Figure S30. The performance of nano-chip in HEK293 cell......S36
Figure S31. CLSM images of HEK293 cells after pre-treated with different miRNA mimics......S37
Figure S32. The relative expression levels of miRNAs in HeLa cells after treated with different miRNA inhibitors......S38
Figure S33. The performance of nano-chip in Hela cells......S39
Figure S34. CLSM images of Hela cells after treatment with nano-chip or discrete DNA logic components......S40
Figure S35. The expression levels of Bcl-2 in HEK293 cells after different treatment......S41
Figure S36. Analyzing apoptosis of Hela cells after different treatments......S42
Figure S37. CLSM images of apoptosis in HEK293 cells after different treatments......S43
Figure S38. The wiring instructions of the AND logic gate with different assembly distance......S44
Figure S39. The wiring instructions of the OR logic gate with different assembly distance......S45
Figure S40. The wiring instructions of the AND-NOT logic gate with different assembly distance......S46
Figure S41. The wiring instructions of three basic logic gates on DNA origami......S47
Figure S42. The wiring instructions of complex DNA cascade circuits on the basis of the basic AND nano-chip......S48
Figure S43. The wiring instructions of complex DNA cascade circuits on the basis of the basic OR nano-chip......S49
Figure S44. The wiring instructions of the scalable nano-chip for multi-levels logic cascading and parallel biocomputing......S50
Figure S45. The wiring instructions of the three layers of cascade logic units on the nano-chip for intracellular molecular biocomputing......S51
Table S1. Sequence of oligonucleotides used in modular logic units......S52
Table S2. Sequence of oligonucleotides used to synthesize DNA origami......S53

Experimental Procedures

Materials and Reagents

Acetic acid, Magnesium acetate, EDTA and Tris base were purchased from Sigma-Aldrich (St. Louis, MO, USA). RIPA Lysis buffer, PMSF and BCA protein assay kit were obtained from Beyotime Biotechnology (Shanghai, China). Cell Counting Kit 8 was provided by Dojindo Laboratories (Kumamoto, Japan). Anti-bcl2 antibody, anti-β-actin antibody, Alexa Fluor 488 annexin V, PI dead cell apoptosis kit, LysoTracker Red DND-99, bisbenzimide H 33342 trihydro chloride (Hoechst 33342), RPMI 1640 medium, fetal bovine serum, Dulbecco's modified Eagle's medium (DMEM), streptomycin and penicillin were obtained from Thermo Fisher Scientific Inc. (MA, USA). HeLa cells and HEK293 cells were provided by the cell bank of Central Laboratory at Xiangya Hospital (Changsha, China). micrOFFTMhsa-miR-30a-5p inhibitors, micrOFF hsa-miR-21-5p inhibitor and micrOFF rno-miR-17-5p inhibitor were purchased from RiboBio Company (Guangzhou, China). M13mp18 was purchased from New England Biolabs (NEB, Ipswich, MA, USA). All other chemicals were of analytical grade and purchased from Sinopharm Chemical Reagent Co. Ltd. (Shanghai, China). All solutions were prepared by using ultrapure water, which was obtained through a Millipore Milli-Q water purification system (Billerica, MA, USA), with an electric resistance of >18.2 MΩ. All DNA oligonucleotides (listed in Table S1 and Table S2) were synthesized and purified by HPLC by Sangon Biological Engineering Technology (Shanghai, China).

Apparatus.

The fluorescence spectra were recorded using Fluorescence Spectrometer FS5 (Edinburgh Instruments, UK). With a 900 V PMT voltage, the excitation and emission slit were set at 5.0 nm. The gel image was obtained via a Tanon 4200SF gel imaging system (Tanon Science & Technology Co., Ltd., China). Atomic force microscopy (AFM) images of samples were measured on a Multimode 8 (Bruker, USA). The flow cytometric analysis was carried on the CytoFLEX flow cytometry system (Beckman, USA). The cell viability was evaluated by a microplate reader (ELx800, BioTek, USA). The fluorescence kinetics experiments were carried out on BIO-RAD PCR instruments (USA). All fluorescence imaging was measured on a confocal laser scanning fluorescence microscope (Nikon, Japan).

Agarose Gel Electrophoresis.

All samples were prepared in advance. The triple-stranded DNA complex (named ABC) and all the duplex DNA complex (such as AR, BR, and DR etc.) were respectively prepared at 10 μM through mixing each DNA strands with an equal concentration and annealing from 90°C to room temperature in 1×TAE buffer (40 mM Tris base, 20 mM Acetic acid, 2 mM EDTA and 12.5 mM Magnesium acetate, pH 8.0). In the gel electrophoresis assay of the DNA strand displacement reactions, 10 μL mixture solution containing 1 μL of 10 μM ABC, 1 μL of 10 μM A_{in-1}, 1 μL of 10 μM B_{in-1} or other given DNA strands was incubated in 1×TAE buffer at 37°C for 2 h, then the obtained samples were mixed with 2 μL of 6×loading buffer and performed on 4% (w/v) agarose gel.

To assess the stability of DNA origami logic chip in complex conditions, 50 nM DNA origami logic chip was dispersed in 10 μL of RPMI-1640 (Gibco) medium including fetal bovine serum (10% (v/v), FBS) or 10 μL of cell lysates (0.5 mL, $1×10^6$ cells mL^{-1}) at 37°C for different times (0, 6, 12, 18, 24, 30, 36, 42, 48, 54, 60 and 72 h). Each sample was mixed with 2 μL of 6×loading buffer and carried out on 1% (w/v) agarose gel.

All the gel electrophoresis assays were performed in 1×Tris-borate-EDTA (TBE) buffer (90 mM Tris-HCl, 2 mM EDTA, 90 mM boric acid and pH 8.0) at 110 V for 2 h at room temperature.

Synthesis of DNA Origami and Preparation of the DNA Origami Logic Chip

DNA origami was synthesized according to the previous reported literature. The mixture solution containing 20 μL of 100 nM M13mp18 scaffold and 80 μL of 1 μM helper strands (including staple strands and capture strands for triple-stranded or duplex DNA complex loading) was annealed in 200 μL of 1×TAE buffer by slowly cooling from 95°C to 25°C through PCR instrument more than 12 h. After that, the resulting solution was centrifuged at 7000g by using 100 kDa MWCO centrifuge filters for three times to remove the additional helper strands. The obtained solution was re-suspended with 1×TAE buffer to stock concentrations of 20 nM and stored at 4°C for future using.

The DNA origami logic chip was prepared by incubating 100 μL of 20 nM DNA origami with 20 μL of 1 μM each DNA logic components (including the triple-stranded DNA complex and the other duplex DNA complex) in 200 μL of 1×TAE buffer at 37°C for 2 h. After that, the additional DNA strands were removed by 100 kDa MWCO centrifuge filters for three times. The obtained solution was re-suspended with 1×TAE buffer to stock concentrations of 20 nM and stored at 4°C for future using.

In Vitro Biocomputing of the DNA Origami Logic Chip

In a typical fluorescence response assay of the DNA origami logic chip, 50 μL reaction mixture containing 40 nM DNA origami logic chip and 200 nM of different input targets was incubated at 37°C for 330min and monitored its fluorescence signal on the real-time fluorescence quantitative PCR instrument.

To compare the kinetic profiles of different samples, the fluorescence intensity at each time point was normalized to the maximum fluorescence intensity observed within that sample during the assay period (expressed as a percentage of the maximum).

Cell Culture and Fluorescence Imaging

HeLa cells and HEK293 cells were cultured in RPMI-1640 (Gibco) medium supplemented with 10% fetal bovine serum, streptomycin (100 μg mL^{-1}) and penicillin (100 units mL^{-1}). All the cells were cultured in a humidified atmosphere (95 % air and 5 % CO_2) at the temperature of 37°C. Before incubated with cell, the DNA origami logic chip should be concentrated to be 100 nM in cold PBS by using 100 kDa MWCO centrifuge filters.

For the biocomputing in the living cells, HEK293 cells (0.5 mL, 1×10^6 cells mL^{-1}) was seeded in a 35-mm confocal dish with 10 mm well and cultured in 2 mL RPMI-1640 medium for 24 h at 37°C. Then, cells were incubated with 250 μL of culture medium containing 300 nM of different input targets and 3 μL Lipo3000 at 37°C for 2 h, followed by incubating with 50 nM DNA origami logic chip and 3 μL Lipo3000 in culture medium at 37°C for another 4 h. Subsequently, the cells were washed three times with cold phosphate buffer saline (PBS) and stained with Hoechst 33342 (10 μg/mL) for 10 min at 37°C before imaging.

For the cell identification, HeLa cells or HEK293 cells (0.5 mL, 1×10^6 cells mL^{-1}) was seeded in a 35-mm confocal dish with 10 mm well and cultured in 2 mL RPMI-1640 medium for 24 h at 37°C. Then, cells were incubated with 250 μL of culture medium containing 50 nM DNA origami logic chip and 3 μL Lipo3000 at 37°C for 4 h. Subsequently, the cells were washed and stained with Hoechst 33342 before imaging.

For the fluorescence imaging of miRNAs inhibition, HeLa cells were pretreat with 250 μM miRNA inhibitors (including MicrOFFTMhsa-miR-30a-5p inhibitor, micrOFF hsa-miR-21-5p inhibitor and micrOFF rno-miR-17-5p inhibitor, respectively) and 6 μL Lipo3000 in 1 mL culture medium at 37°C for 24 h. Then, the cells were incubated with DNA origami logic chip, washed three times with cold PBS and stained with Hoechst 33342 before imaging.

For the fluorescence imaging of bcl-2 silencing, the cells were treated with the given reagents at 37°C for 4 h, and kept culturing in RPMI-1640 medium for another 24 h. After that, the cells were fixed in 4% paraformaldehyde for 20 min, treated 0.2% Triton X-100 for permeabilization, and added 5% BSA for another 30 min at room temperature. Subsequently, the cells were successively incubated with 2 μg mL^{-1} anti-bcl-2 antibodies and 4 μg mL^{-1} the secondary antibodies of m-IgGk BP-CFL 488 in 1×PBS (6.7 mM, pH 7.4) at room temperature for 1 h, followed by washing with 1×PBS before imaging.

All fluorescence images were acquired by using an oil dipping objective (60×) on a Nikon confocal laser scanning fluorescence microscope at a sampling speed of 6.2 pixel/dwell and a size of 1024.

Determination of Cell Viability

HeLa cells or HEK293 cells (5×10^3 cells) were seed on 96-well plates in the RPMI 1640 medium for 24 h under 5 wt %/vol CO_2 atmosphere. Subsequently, the cells were treated with different concentrations of DNA origami logic chip (0, 40, 80, 100, 150, 200 nM) for 24 h. Subsequently, the cells were washed twice with cold PBS and incubated with 100 μL culture medium containing 10 μL of CCK-8 stored solution at 37°C for another 2 h. Finally, the absorbance at 450 nm was measured on a microplate reader to calculate the cell viability.

Western Blot Analysis

HeLa cells or HEK293 cells (0.5 mL, 1×10^6 cells mL^{-1}) were incubated with the given DNA origami logic chip at 37°C for 4 h, and kept culturing in RPMI-1640 medium for another 24 h. Subsequently, the cells were collected and lysed in 75 μL of lysis buffer (50 mM Tris, 150 mM NaCl, 1% Triton X-100, 1% sodium deoxycholate, 5 mM EDTA, 1 mM PMSF, 0.1% SDS, 2 μg mL^{-1} each of sodium orthovanadate, sodium fluoride, leupeptin and pH 7.4) at 4 °C for 30min. Then the lysate was centrifuged at 13000 g for 15 min to collected the supernatant. The concentration of total cellular proteins in the supernatant was determined by using BCA protein assay kit. Next, the total cellular proteins were separated and transferred to PVDF membranes. After incubated with anti-bcl-2 antibodies and secondary antibodies, the membranes were sent to detection on Gel Imaging System (Bio-RAD).

Flow Cytometry Experiments

HeLa cells (0.5 mL, 1×10^6 cells mL^{-1}) were seeded in a 35-mm dish and cultured in RPMI-1640 medium at 37°C for 24 h. Subsequently, the cells were incubated with the given reagents, then treated with 0.25% trypsin for 2 min and centrifuged at 1000 g for 5 min followed by washing with PBS twice. Finally, the cells were re-dispersed in 500 μL of cold PBS for flow cytometric analysis on a Cytoflex flow cytometry system.

For apoptosis analysis, the cells (0.5 mL, 1×10^6 cells mL^{-1}) were incubated with the given reagents for 4 h at 37°C. Then the cells were washed twice with cold PBS and continue cultured in RPMI-1640 medium for 24 h. After that, the cells were treated with 2 μg mL^{-1} Alexa Fluor 488 annexin V and 10 μg mL^{-1} PI dye in 1 mL cold PBS at room temperature for 15 min. Then cells were re-suspended in 1 mL cold PBS followed by flow cytometry assay with FITC and PI channel.

Real-Time Reverse Transcriptase-PCR Analysis

HeLa cells or HEK293 cells (0.5 mL, 1×10^6 cells mL^{-1}) were seeded in a 35-mm dish, cultured in 3 mL RPMI-1640 medium for 24 h at 37°C, and incubated with the given reagents for 4 h at 37°C. Then the cells were washed twice with cold PBS and continue cultured in RPMI-1640 medium for 24 h. After that, the cells were treated with the given reagents. After 24 h cultivation, the total cellular RNAs were extracted from HeLa cells by using Uniq-10 column Trizol total RNA extraction kit (Sangon) according to the manufacturer's instructions. The cDNA samples were prepared with Revert Aid Premium Reverse Transcriptase (Thermo Fisher Scientific)

according to the indicated protocol. The analysis of cDNA was performed with SybrGreen PCR Master Mix (ABI, USA) on an ABI StepOnePlus qPCR instrument. The 20 μL reaction solution contained 2 μL cDNA sample, 10 μL of SG Fast qPCR Master Mix (High Rox, 2×), 0.4 μL of primer forward (10 μM), 0.4 μL of primer reverse (10 μM) and 7.2 μL of the nuclease-free water. The PCR conditions were as follows: an initial 95°C for 3 min followed by 40 cycles for 15 s at 95°C, for 20 s at 57°C and for 30 s at 72°C. The endogenous beta-actin mRNA was used as control for quantifying the relative level of bcl-2 mRNA. The target miRNAs were absolutely quantified by using fluorescent quantitative PCR method. The primers for bcl-2 used as follow: bcl-2 forward primer, 5'-gctggattataactcctcttctttc-3'; bcl-2 reverse primer, 5'- ctctgcgacagcttataatggat-3'. The primers for miRNA-21 used as follow: miRNA-21 forward primer, 5'- acactccagctgggtagcttatcagact-3'; miRNA-21 reverse primer, 5'-ctcaactggtgtcgtggagtcggcaattcagttgagtcaacatc-3'. The primers for miRNA17 used as follow: miRNA-17 forward primer, 5'-acactccagctgggcaaagtgcttacagtg-3'; miRNA-17 reverse primer, 5'-ctcaactggtgtcgtggagtcggcaattcagttgagctacctg-3'. The primers for miRNA-30a used as follow: miRNA-30a forward primer, 5'-acactccagctgggtgtaaacatcctcg-3'; miRNA-30a reverse primer, 5'-ctcaactggtgtcgtggagtcggcaattcagttgagcttccagt-3'.

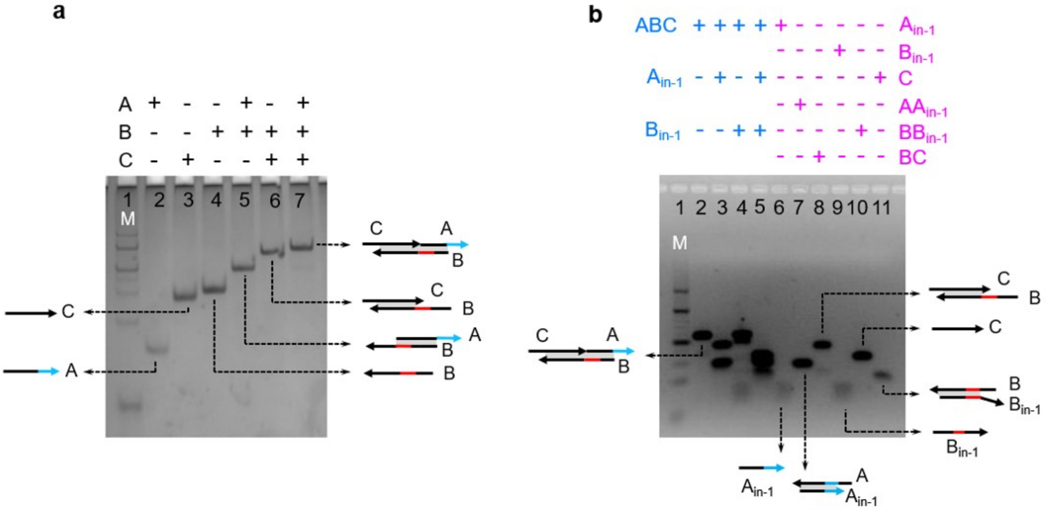

Figure S1. (a) Agarose gel electrophoresis analysis of the assembly of triple-stranded DNA complex ABC. (b) Agarose gel electrophoresis analysis of the toehold mediated strand displacement reaction between ssDNA (A_{in-1}, B_{in-1}, or both of the two ssDNA) and the complex ABC. Lane 2-5 showed the products of the reactions between ssDNA (A_{in-1}, B_{in-1}, or both of the two ssDNA) and the complex ABC marked in blue words. Lane 6-11 exhibited the products of the corresponding ssDNA and DNA duplexes marked in purple-red words. The error bars indicate mean $\pm$ standard deviation (SD, n = 5).

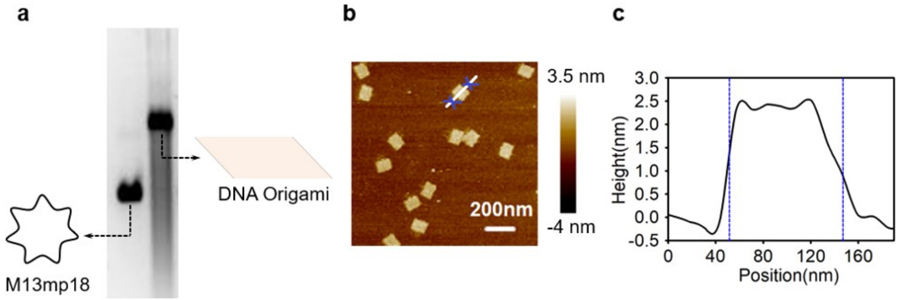

Figure S2. (a) Agarose gel electrophoresis analysis of the DNA origami. (b) Atomic force microscopy (AFM) image of the DNA origami. (c) Height profile of a cross-section of origami. The height of DNA origami is 2 nm.

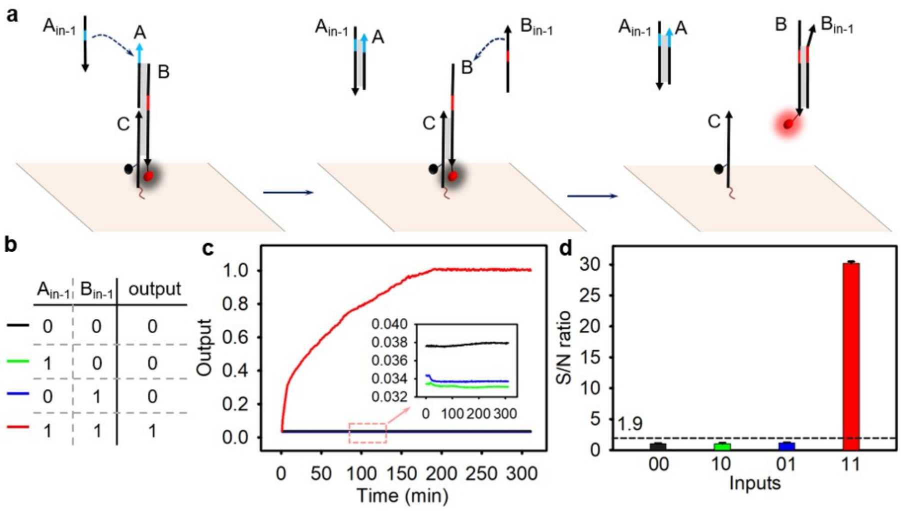

Figure S3. Reaction performance of basic DNA units (the triple-stranded DNA complex ABC) on DNA origami. (a) Schematic illustration of the triple-stranded DNA complex reacted with ssDNA A_{in-1} and B_{in-1}. (b) The truth value table of the logic reactions. (c) Time-dependent Cy5 fluorescence signals (output) of the triple-stranded DNA complex ABC response to different inputs. (d) Fluorescence intensity of the triple-stranded DNA complex ABC response to different inputs after 200 min of reaction. The true/false threshold (1/0) was calculated to be 1.9, which was the mean maximum value of false outputs plus 10 times of its standard deviation. The error bars indicate mean ± standard deviation (SD, n = 5).

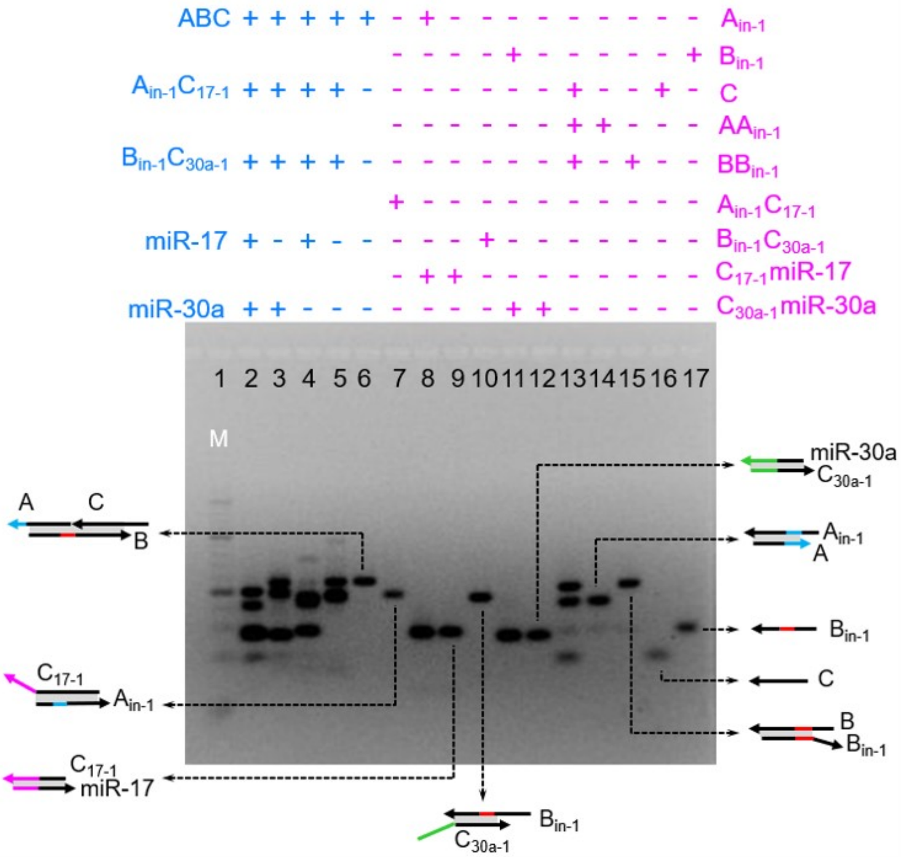

Figure S4. Agarose gel electrophoresis analysis of the toehold mediated strand displacement reaction between inputs (miRNA-17 and miRNA-30a) and the functional DNA units, including two DNA duplex $A_{in-1}C_{17-1}$, $B_{in-1}C_{30a-1}$ and the triple-stranded complex ABC. Lane 2-6 showed the products of the reactions between inputs and the functional DNA units marked in blue words. Lane 7-17 exhibited the products of the corresponding ssDNA and DNA duplexes marked in purple-red words.

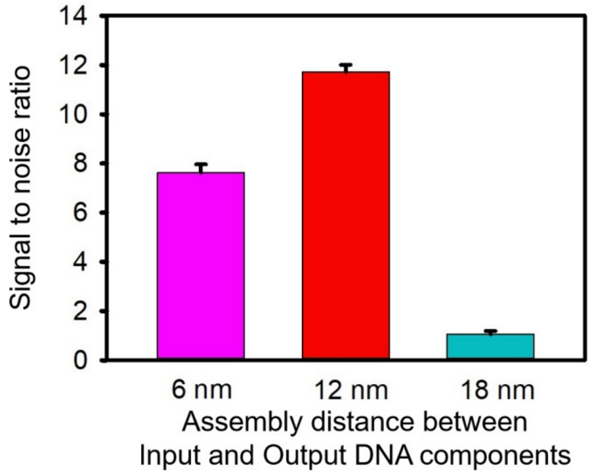

Figure S5. The optimizing the assembly distance between input and output DNA components on the AND logic nano-chip. The error bars indicate mean± standard deviation (SD, n = 5). The more detailed descriptions about wiring instructions were given in Figure S43.

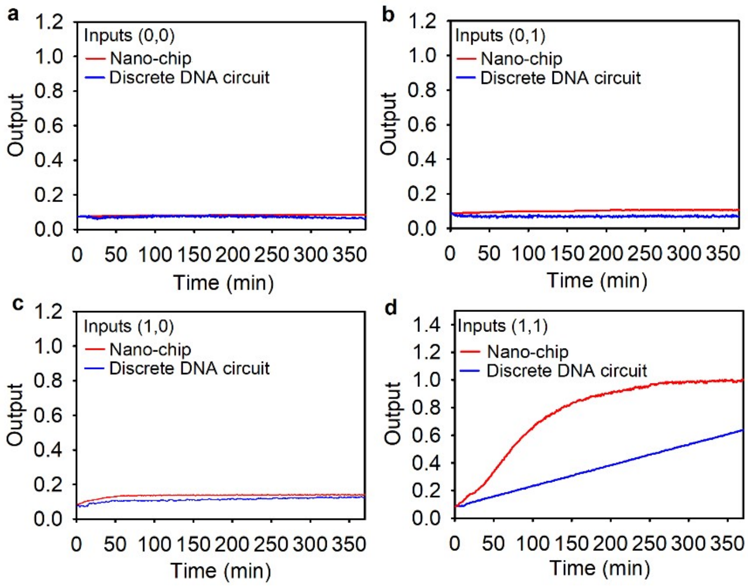

Figure S6. Time-dependent Cy5 fluorescence signals (output) of the AND gate in nano-chip or discrete DNA circuit response to different inputs (miRNA-17 and miRNA 30a). (a) The basic fluorescence signals of AND gate in nano-chip or discrete DNA circuit without incubation of miRNAs [Inputs(0,0)]; (b) the fluorescence signals of AND gate in nano-chip or discrete DNA circuit incubated only miRNA-30a [Inputs(0,1)]; (c) the fluorescence signals of AND gate in nano-chip or discrete DNA circuit incubated with only miRNA-17 [Inputs(1,0)]; (d) the fluorescence signals of AND gate in nano-chip or discrete DNA circuit incubated with both two miRNAs [Inputs(1,1)]. Red lines represented the fluorescence signals of AND gate in nano-chip responsed to inputs, blue lines represented the fluorescence signals of AND gate in discrete DNA circuit responsed to inputs. The data were further fitted to a first-order kinetic model, and the kinetic rate constant was 0.0013 min^{-1} and 0.0303 min^{-1} for the diffusible system and nano-chip, respectively.

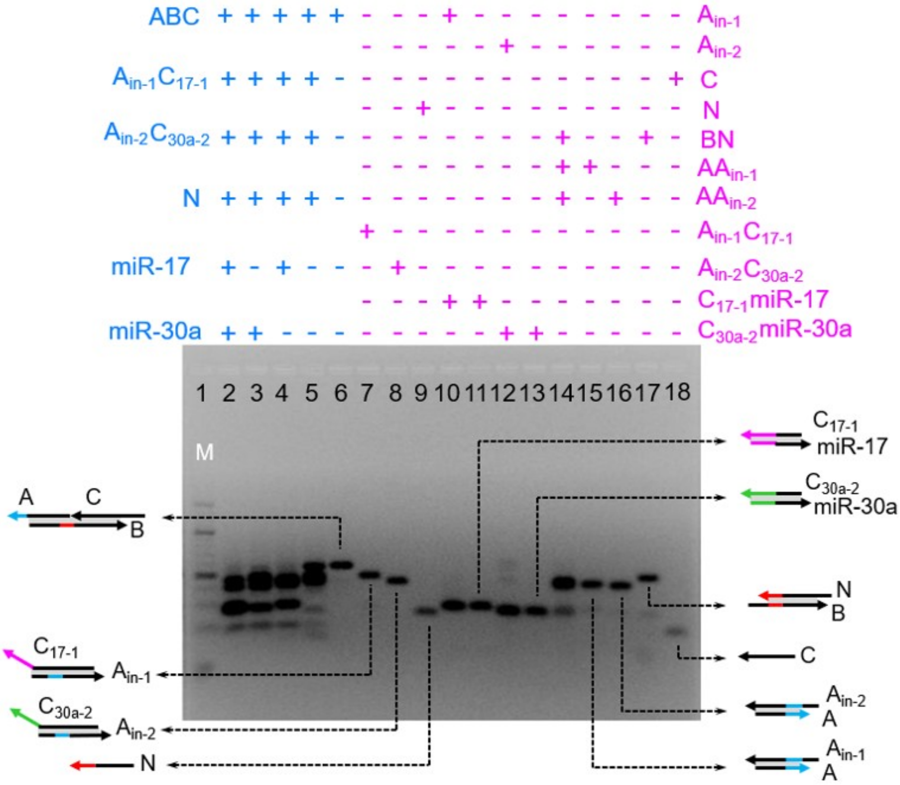

Figure S7. Agarose gel electrophoresis analysis of the toehold mediated strand displacement reaction between inputs (miRNA-17 and miRNA-30a) and the functional DNA units, including ssDNA N, two DNA duplex $A_{in-1}C_{17-1}$, $A_{in-2}C_{30a-2}$ and the triple-stranded complex ABC. Lane 2-6 showed the products of the reactions between inputs and the functional DNA units marked in blue words. Lane 7-18 exhibited the products of the corresponding ssDNA and DNA duplexes marked in purple-red words.

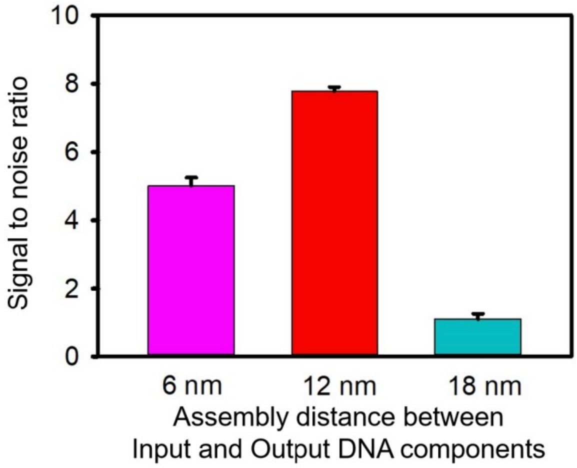

Figure S8. The optimizing the assembly distance between input and output DNA components on the AND logic nano-chip. The error bars indicate mean ± standard deviation (SD, n = 5). The more detailed descriptions about wiring instructions were given in Figure S44.

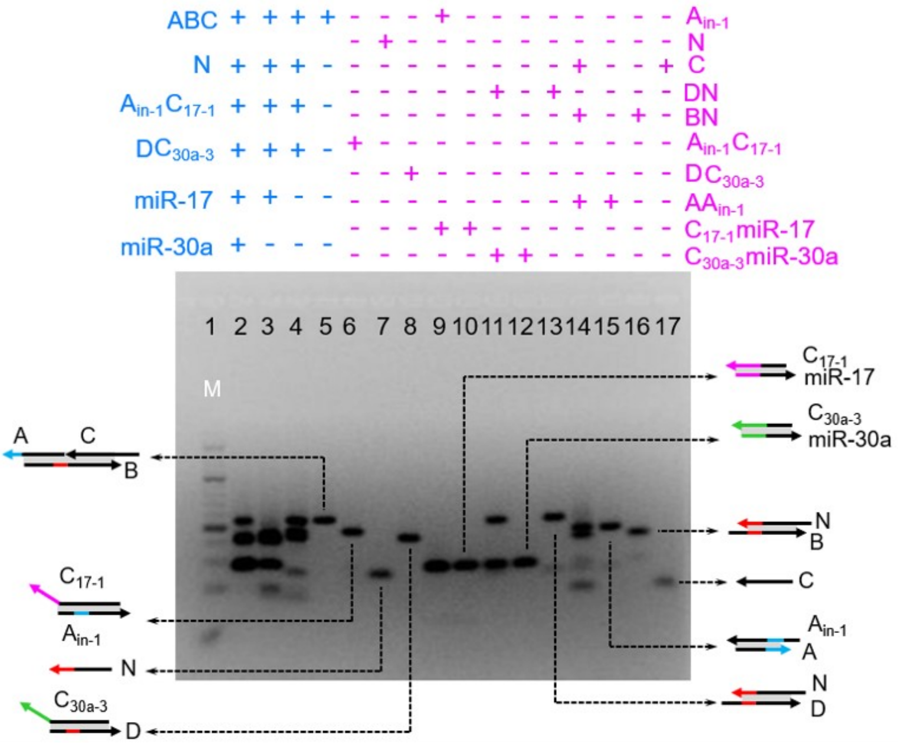

Figure S9. Agarose gel electrophoresis analysis of the toehold mediated strand displacement reaction between inputs (miRNA-17 and miRNA-30a) and the functional DNA units, including ssDNA N, two DNA duplex $A_{in-1}C_{17-1}$, DC_{30a-3} and the triple-stranded complex ABC. Lane 2-5 showed the products of the reactions between inputs and the functional DNA units marked in blue words. Lane 6-17 exhibited the products of the corresponding ssDNA and DNA duplexes marked in purple-red words.

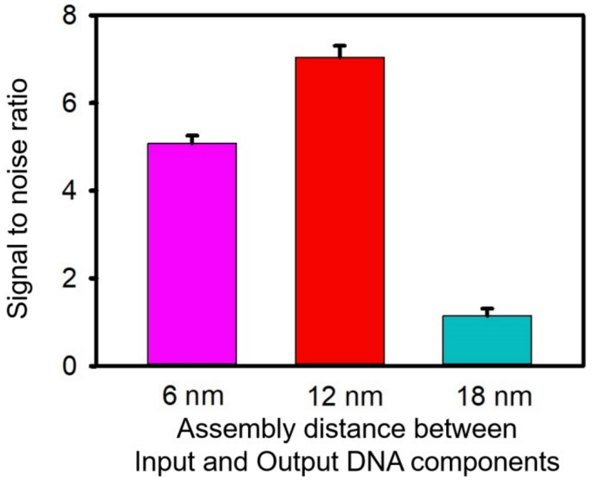

Figure S10. The optimizing the assembly distance between input and output DNA components on the AND-NOT logic nano-chip. The error bars indicate mean± standard deviation (SD, n = 5). The more detailed descriptions about wiring instructions were given in Figure S45.

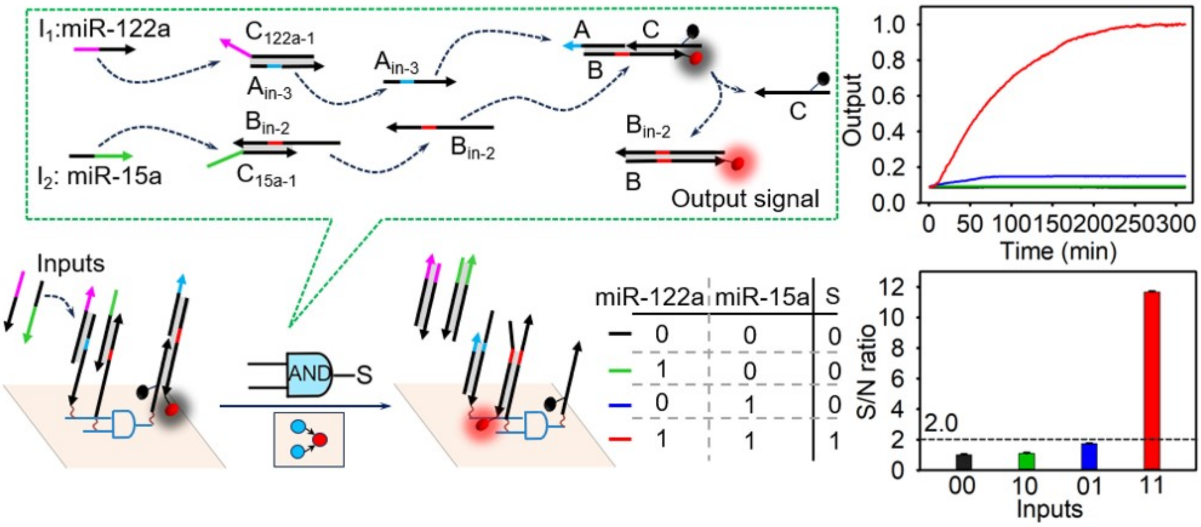

Figure S11. Design and performance of the general AND logic nano-chip using two microRNAs (-122a, -15a) as inputs. Brief, two duplex DNA complex $A_{in-3}C_{122a-1}$, $B_{in-2}C_{15a-1}$ and a triple-stranded DNA complex ABC were spatially arranged by wiring instruction to form origami-based AND gate. Two duplex DNA complex $A_{in-3}C_{122a-1}$ and $B_{in-2}C_{15a-1}$ converted the input microRNAs (-122a and -15a) to single-stranded DNA (ssDNA, A_{in-3}, B_{in-2}), and the latter hybridized with the triple-stranded DNA complex ABC to release ssDNA C and produce new duplex DNA complex BB_{in-2}, lighting up the fluorescence signal. As expected in the truth value table, when all the two miRNAs were present (1,1), a strong fluorescence signal was obtained with the true/false (1/0) threshold was calculated to be 2.0 and the fluorescence recovery almost reached the maximum after 240 min incubation. The error bars indicate mean ± standard deviation (SD, n = 5).

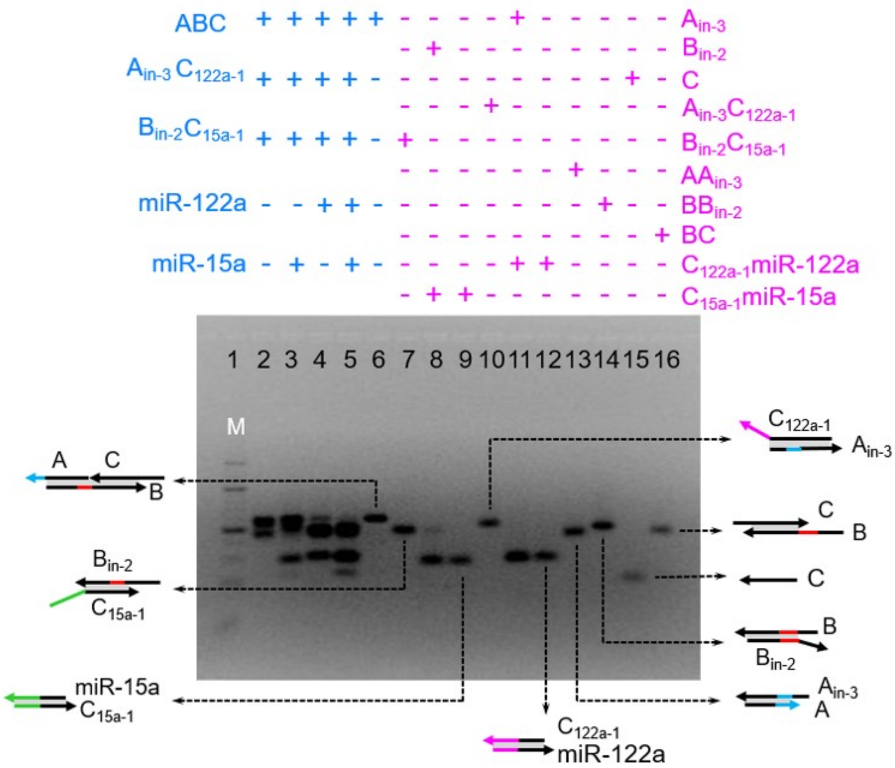

Figure S12. Agarose gel electrophoresis analysis of the toehold mediated strand displacement reaction between inputs (miRNA-122a and miRNA-15a) and the functional DNA units, including two DNA duplex $A_{in-3}C_{122a-1}$, $B_{in-2}C_{15a-1}$ and the triple-stranded complex ABC. Lane 2-6 showed the products of the reactions between inputs and the functional DNA units marked in blue words. Lane 7-16 exhibited the products of the corresponding ssDNA and DNA duplexes marked in purple-red words.

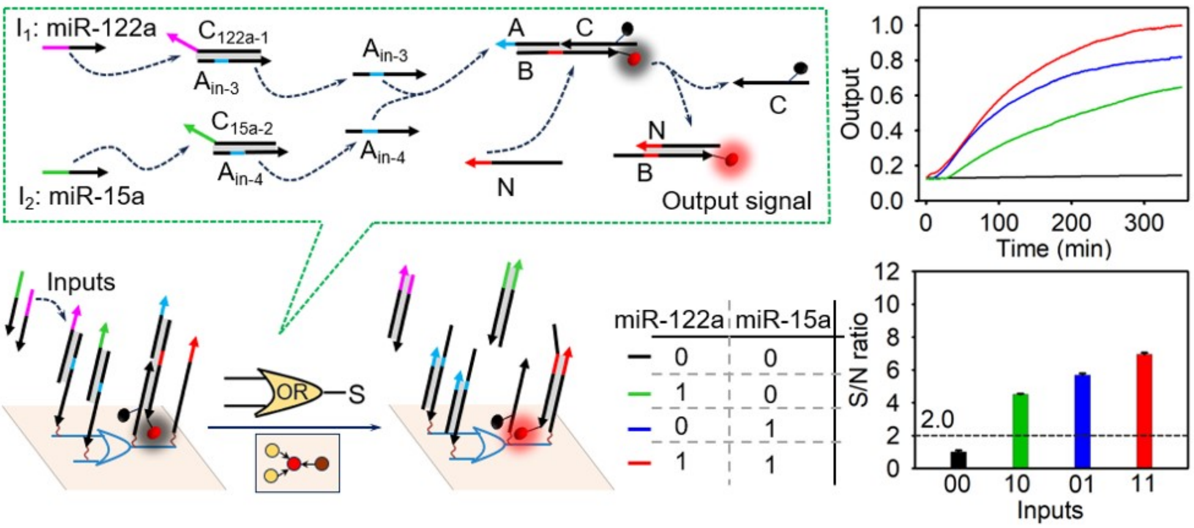

Figure S13. Design and performance of the general OR logic nano-chip using two microRNAs (-122a, -15a) as inputs. Brief, one or both two input miRNAs displaced ssDNA $A_{in\text{-}3}$ or $A_{in\text{-}4}$ from duplex DNA complex $A_{in\text{-}3}C_{122a\text{-}1}$ or $A_{in\text{-}4}C_{15a\text{-}2}$, and the latter hybridized with the triple-stranded DNA complex ABC to release duplex DNA complex BC. Then, ssDNA N liberated ssDNA C and produced duplex DNA complex BN, lighting up the fluorescence signal. As expected in the truth value table, when either one or both inputs were present (0,1), (1,0), (1,1) resulted in significant output fluorescenc, and the true/false (1/0) threshold was calculated to be 2.0. The error bars indicate mean± standard deviation (SD, n = 5).

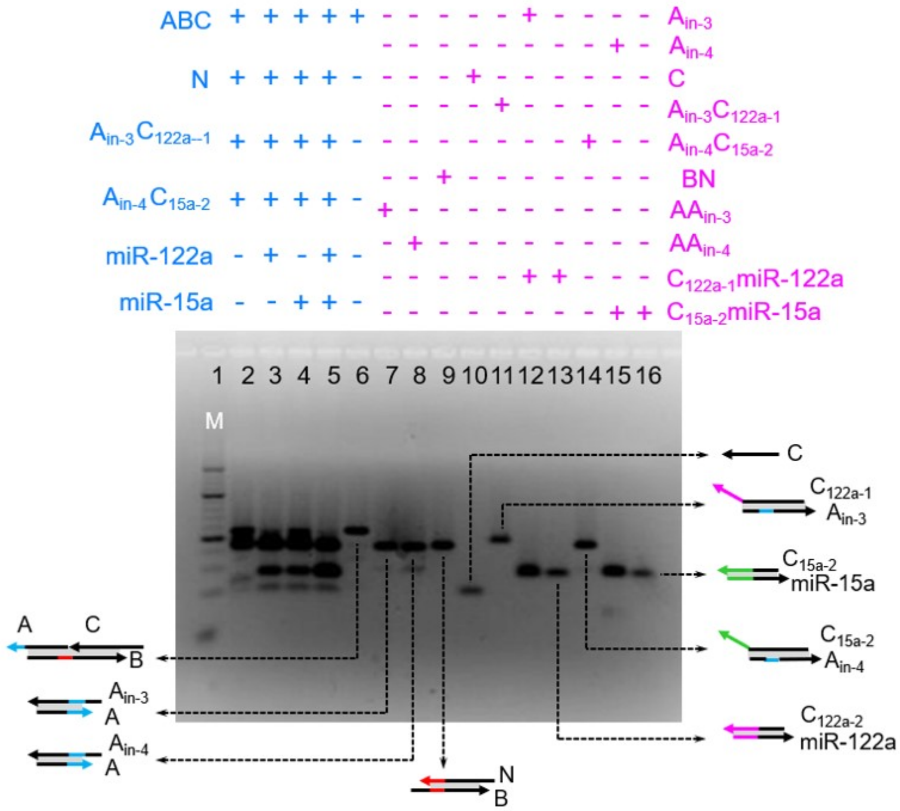

Figure S14. Agarose gel electrophoresis analysis of the toehold mediated strand displacement reaction between inputs (miRNA-122a and miRNA-15a) and the functional DNA units, including ssDNA N, two DNA duplex $A_{in-3}C_{122a-1}$, $A_{in-4}C_{15a-2}$ and the triple-stranded complex ABC. Lane 2-6 showed the products of the reactions between inputs and the functional DNA units marked in blue words. Lane 7-16 exhibited the products of the corresponding ssDNA and DNA duplexes marked in purple-red words.

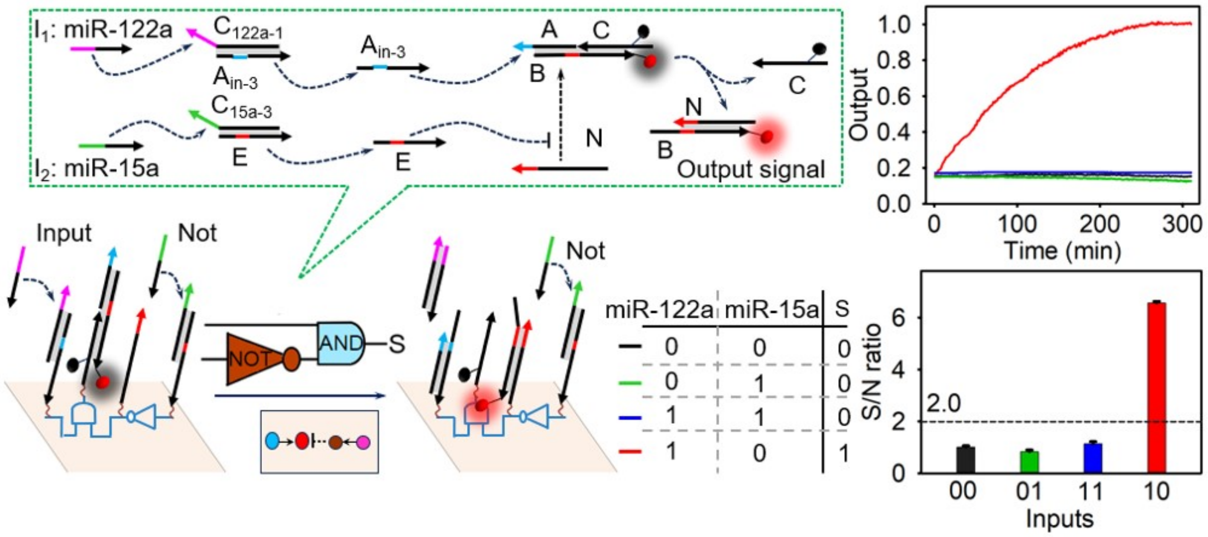

Figure S15. Design and performance of the general NOT-AND logic nano-chip using two microRNAs (-122a, -15a) as inputs. Brief, input miRNA-122a displaced ssDNA A_{in-3} from duplex DNA complex $A_{in-3}C_{122a-1}$, and the latter hybridized with the triple-stranded DNA complex ABC to release duplex DNA complex BC. In the absence of miRNA-15a, ssDNA N liberated ssDNA C and produced duplex DNA complex BN, lighting up the fluorescence signal. Once in the presence of miRNA-15a, duplex DNA complex EC_{15a-3} converted it to the ssDNA E, which could preferentially hybridize with ssDNA N to prevent subsequent displacement reaction. As expected in the truth value table, the logic cascade nano-chip generated high output signal only in the presence of miRNA-122a and absence of miRNA-15a, and the true/false (1/0) threshold was calculated to be 2.0.

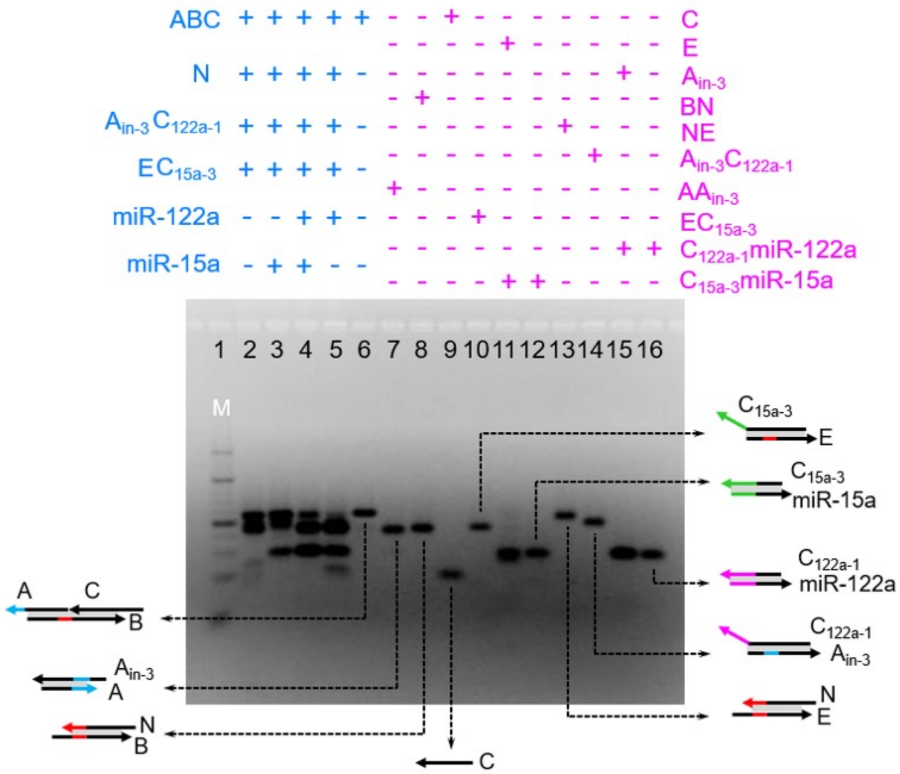

Figure S16. Agarose gel electrophoresis analysis of the toehold mediated strand displacement reaction between inputs (miRNA-122a and miRNA-15a) and the functional DNA units, including ssDNA N, two DNA duplex $A_{in-3}C_{122a-1}$, EC_{15a-3} and the triple-stranded complex ABC. Lane 2-6 showed the products of the reactions between inputs and the functional DNA units marked in blue words. Lane 7-16 exhibited the products of the corresponding ssDNA and DNA duplexes marked in purple-red words.

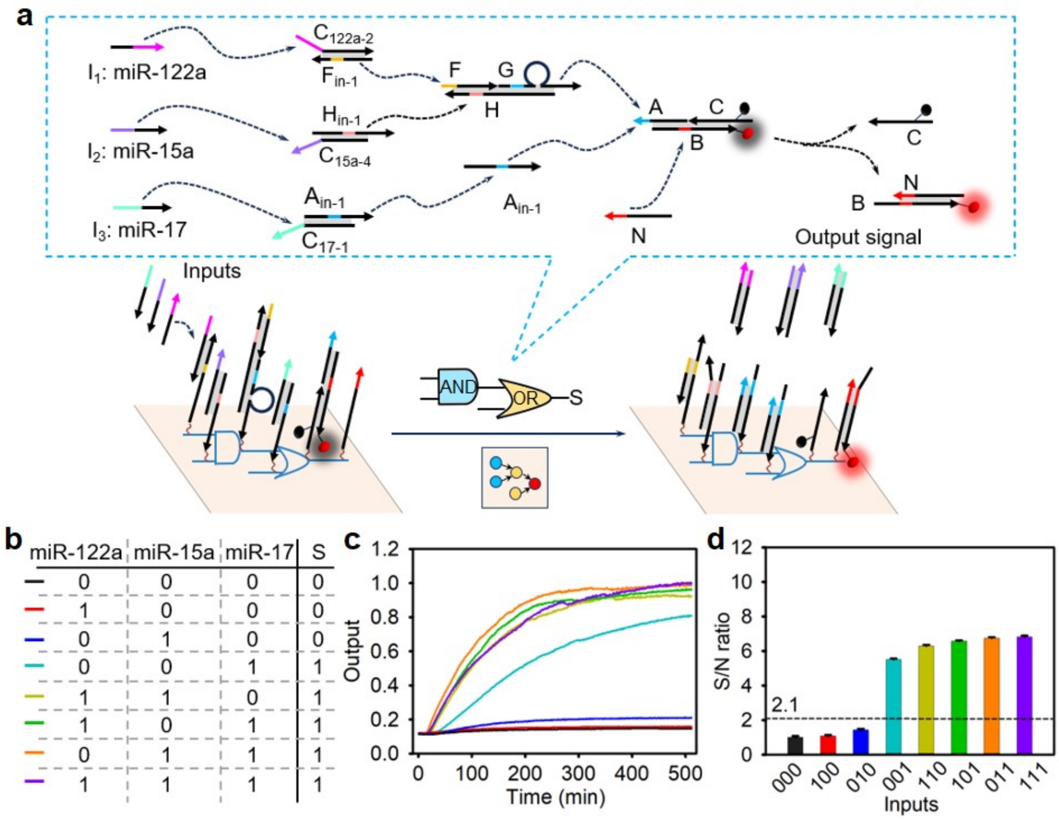

Figure S17. Design and performance of the AND-OR logic nano-chip using three microRNAs (-122a, -15a and -17) as inputs. Brief, (a) two duplex DNA complex $F_{in-1}C_{122a-2}$ and $H_{in-1}C_{15a-4}$ converted the input microRNAs (-122a and -15a) to single-stranded DNA (ssDNA, F_{in-1}, H_{in-1}), and the latter hybridized with the triple-stranded DNA complex FGH to release ssDNA G; Input miR-17 displaced ssDNA A_{in-1} from duplex DNA complex $A_{in-1}C_{17-1}$. One of ssDNA G or ssDNA A_{in-1} could hybridize with the triple-stranded DNA complex ABC to release duplex DNA complex BC. Then, ssDNA N liberated ssDNA C and produced duplex DNA complex BN, lighting up the fluorescence signal. (b) The truth value table of AND-OR logic nano-chip. (c) Time-dependent reactivity of AND-OR logic nano-chip (40 nM) corresponding to different inputs (the concentration of each input miRNAs was 40 nM). (d) S/N ratio obtained from (c) with fluorescence intensity at 300 min. The more detailed descriptions about wiring instructions were given in Figure S42a.

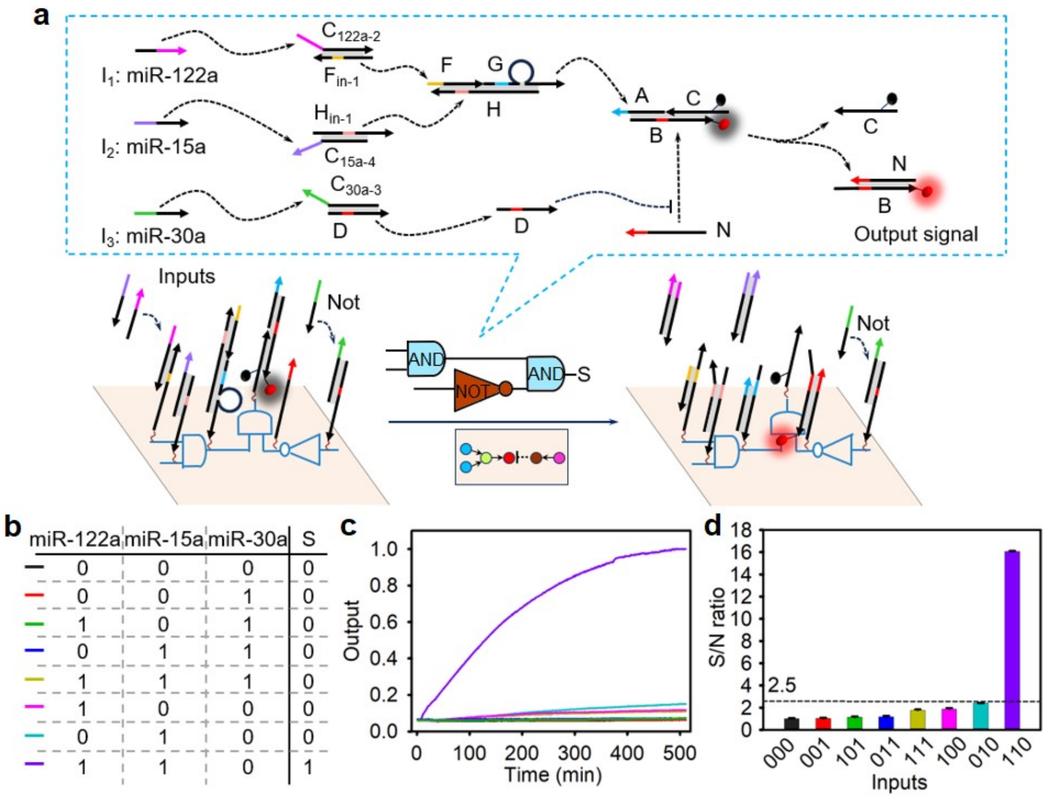

Figure S18. Design and performance of the AND-NOT-AND logic nano-chip using three microRNAs (-122a, -15a and -30a) as inputs. Brief, (a) two duplex DNA complex $F_{in-1}C_{122a-2}$ and $H_{in-1}C_{15a-4}$ converted the input microRNAs (-122a and -15a) to single-stranded DNA (ssDNA, F_{in-1}, H_{in-1}), and the latter hybridized with the triple-stranded DNA complex FGH to release ssDNA G. Then the ssDNA G hybridized with the triple-stranded DNA complex ABC to release duplex DNA complex BC. Subsenquently, ssDNA N liberated ssDNA C and produced duplex DNA complex BN, lighting up the fluorescence signal.Once in the presence of miRNA-30a, duplex DNA complex DC_{30a-3} converted it to the ssDNA D, which could preferentially hybridize with ssDNA N to prevent subsequent displacement reaction. (b) The truth value table of AND-NOT-AND logic nano-chip. (c) Time-dependent reactivity of AND-NOT-AND logic nano-chip (40 nM) corresponding to different inputs (the concentration of each input miRNAs was 40 nM). (d) S/N ratio obtained from (c) with fluorescence intensity at 500 min. The more detailed descriptions about wiring instructions were given in Figure S42b.

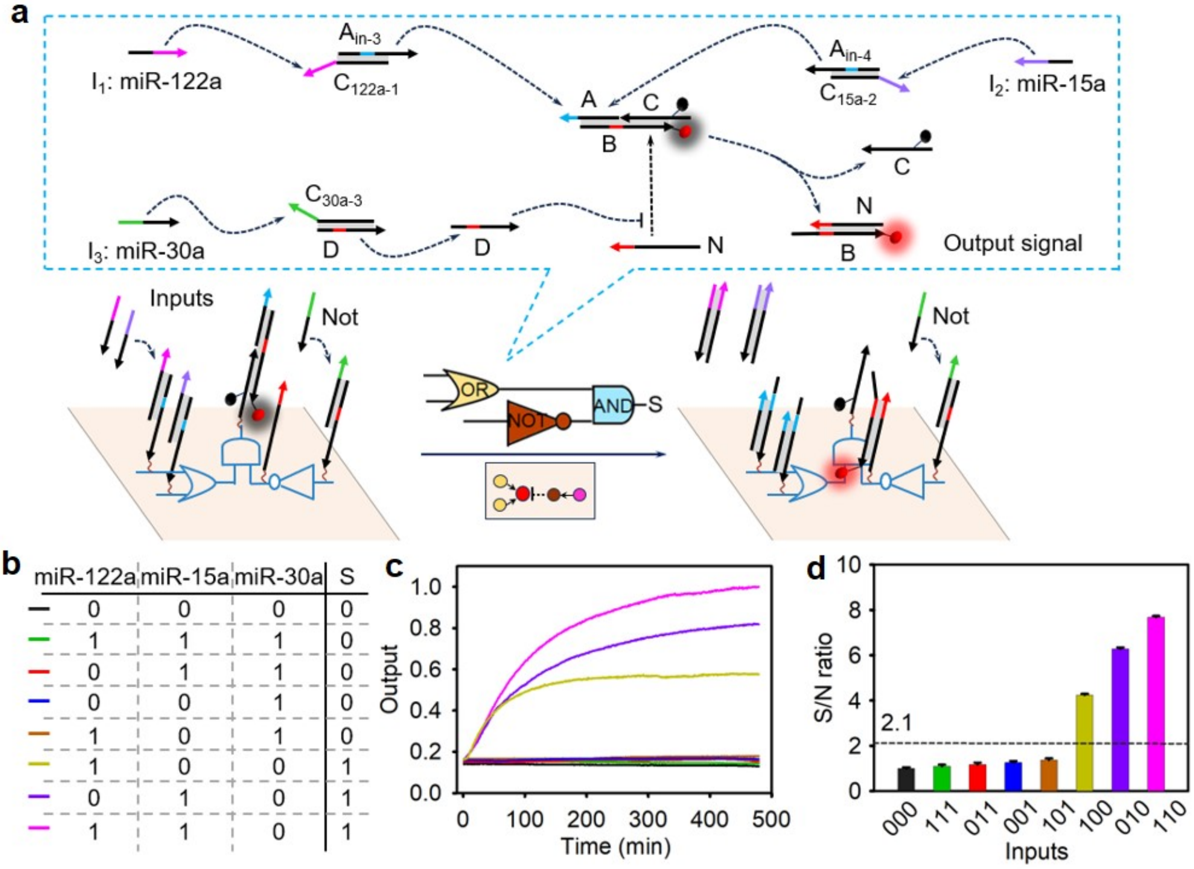

Figure S19. Design and performance of the OR-NOT-AND logic nano-chip using three microRNAs (-122a, -15a and -30a) as inputs. Brief, (a) one or both two input miRNAs displaced ssDNA A_{in-3} or A_{in-4} from duplex DNA complex $A_{in-3}C_{122a-1}$ or $A_{in-4}C_{15a-2}$, and the latter hybridized with the triple-stranded DNA complex ABC to release duplex DNA complex BC. Then, ssDNA N liberated ssDNA C and produced duplex DNA complex BN, lighting up the fluorescence signal. Once in the presence of miRNA-30a, duplex DNA complex DC_{30a-3} converted it to the ssDNA D, which could preferentially hybridize with ssDNA N to prevent subsequent displacement reaction. (b) The truth value table of OR-NOT-AND logic nano-chip. (c) Time-dependent reactivity of OR-NOT-AND logic nano-chip (40 nM) corresponding to different inputs (the concentration of each input miRNAs was 40 nM). (d) S/N ratio obtained from (c) with fluorescence intensity at 500 min. The more detailed descriptions about wiring instructions were given in Figure S43a.

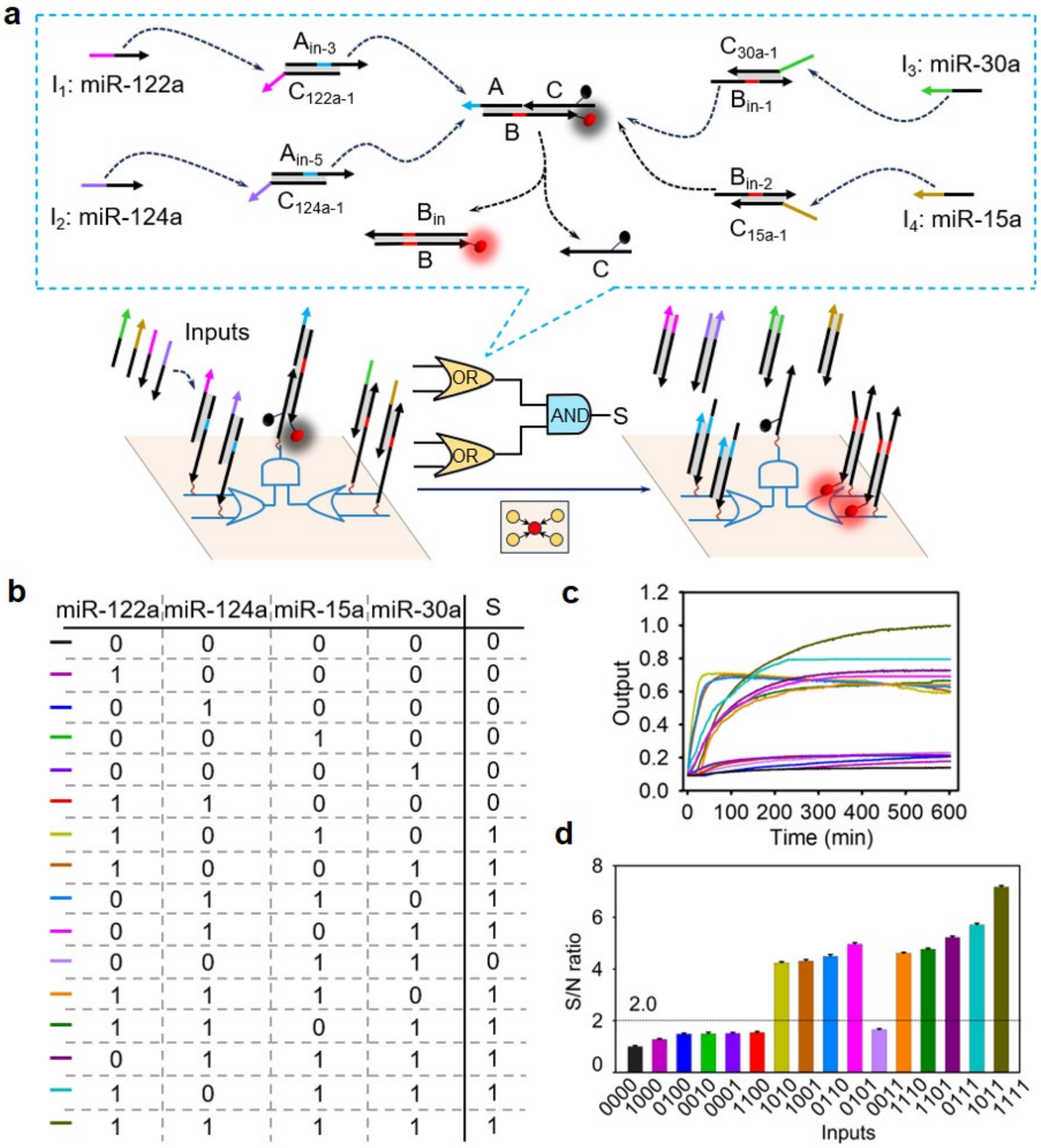

Figure S20. Design and performance of the OR-OR-AND logic nano-chip using three microRNAs (-122a, -124a, -15a and -30a) as inputs. Brief, (a) one or both two input miRNAs displaced ssDNA A_{in-3} or A_{in-5} from duplex DNA complex $A_{in-3}C_{122a-1}$ or $A_{in-5}C_{124a-1}$, and the latter hybridized with the triple-stranded DNA complex ABC to release duplex DNA complex BC. At the same time, one or both two input miRNAs displaced ssDNA B_{in-1} or B_{in-2} from duplex DNA complex $B_{in-1}C_{30a-1}$ or $B_{in-2}C_{15a-1}$, and the latter hybridized with the duplex DNA complex BC to liberate ssDNA C, lighting up the fluorescence signal. (b) The truth value table of OR-OR-AND logic nano-chip. (c) Time-dependent reactivity of OR-OR-AND logic nano-chip (40 nM) corresponding to different inputs (the concentration of each input miRNAs was 40 nM). (d) S/N ratio obtained from (c) with fluorescence intensity at 500 min. The more detailed descriptions about wiring instructions were given in Figure S43b.

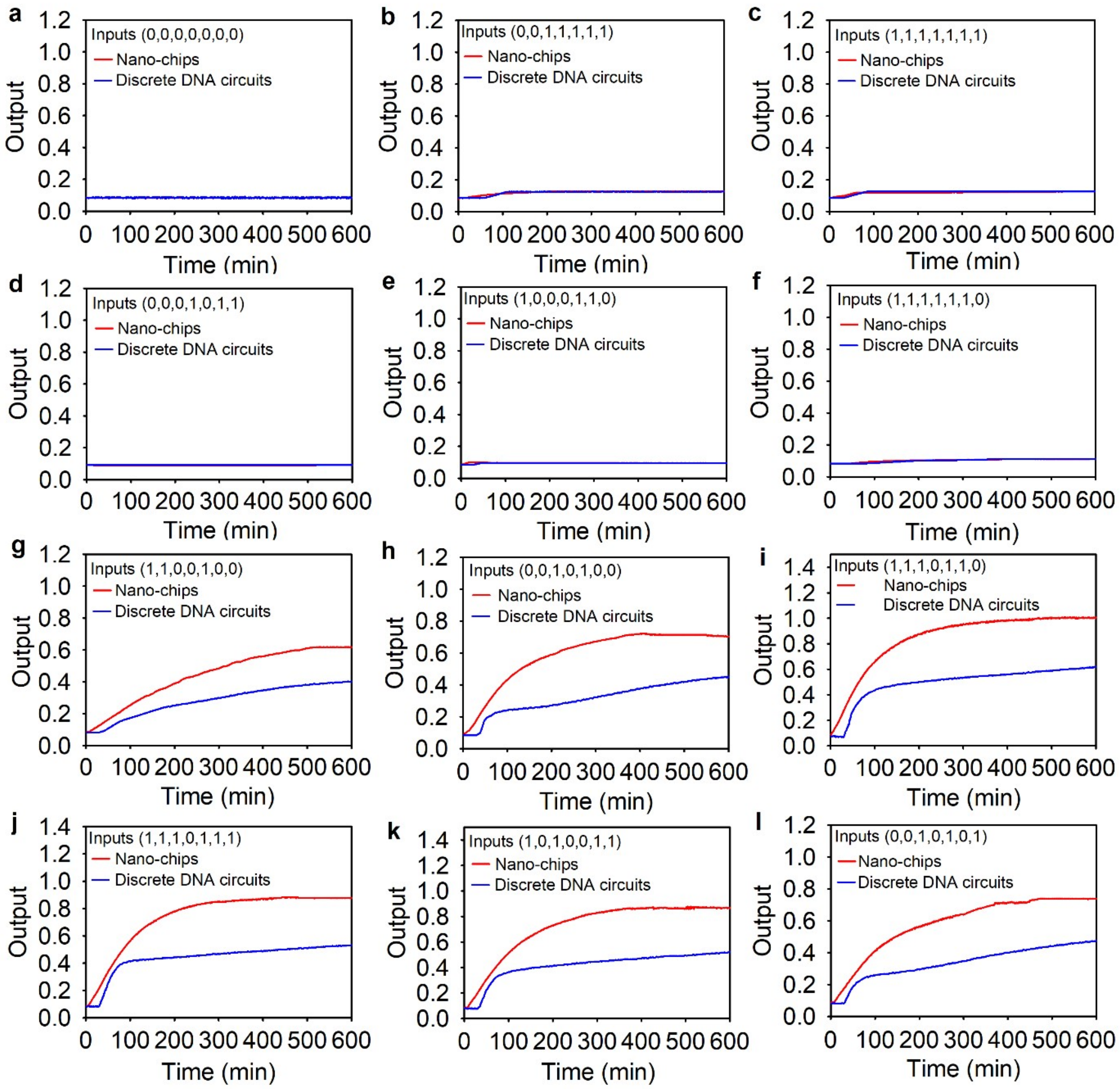

Figure S21. Time-dependent Cy5 fluorescence signals (Output S_0) of the AND-OR-NOT-AND circuit in nano-chip or discrete DNA circuit response to different inputs (the input order was miR-122a, -15a, -17, -30a, miR-124a, -143 and -10b). (a) The fluorescence signals of Inputs (0, 0, 0, 0, 0, 0, 0); (b) the fluorescence signals of Inputs (0, 0, 1, 1, 1, 1, 1); (c) the fluorescence signals of Inputs (1, 1, 1, 1, 1, 1, 1); (d) the fluorescence signals of Inputs (0, 0, 0, 1, 0, 1, 1); (e) the fluorescence signals of Inputs (1, 0, 0, 0, 1, 1, 0); (f) the fluorescence signals of Inputs (1, 1, 1, 1, 1, 1, 0); (g) the fluorescence signals of Inputs (1, 1, 0, 0, 1, 0, 0), the kinetic rate constant was 0.0010 min^{-1} and 0.0022 min^{-1} for the diffusible system and nano-chip, respectively; (h) the fluorescence signals of Inputs (0, 0, 1, 0, 1, 0, 0), the kinetic rate constant was 0.0021 min^{-1} and 0.0056 min^{-1} for the diffusible system and nano-chip, respectively; (i) the fluorescence signals of Inputs (1, 1, 1, 0, 1, 1, 0), the kinetic rate constant was 0.0043 min^{-1} and 0.0166 min^{-1} for the diffusible system and nano-chip, respectively; (j) the fluorescence signals of Inputs (1, 1, 1, 0, 1, 1, 1), the kinetic rate constant was 0.0011 min^{-1} and 0.0093 min^{-1} for the diffusible system and nano-chip, respectively; (k) the fluorescence signals of Inputs (1, 0, 1, 0, 0, 1, 1), the kinetic rate constant was 0.0009min^{-1} and 0.0083 min^{-1} for the diffusible system and nano-chip, respectively; (l) the fluorescence signals of Inputs (0, 0, 1, 0, 1, 0, 1), the kinetic rate constant was 0.0009 min^{-1} and 0.0050 min^{-1} for the diffusible system and nano-chip, respectively. Red lines represented the fluorescence signals of nano-chip responsed to inputs, blue lines represented the fluorescence signals of discrete DNA circuit responsed to inputs.

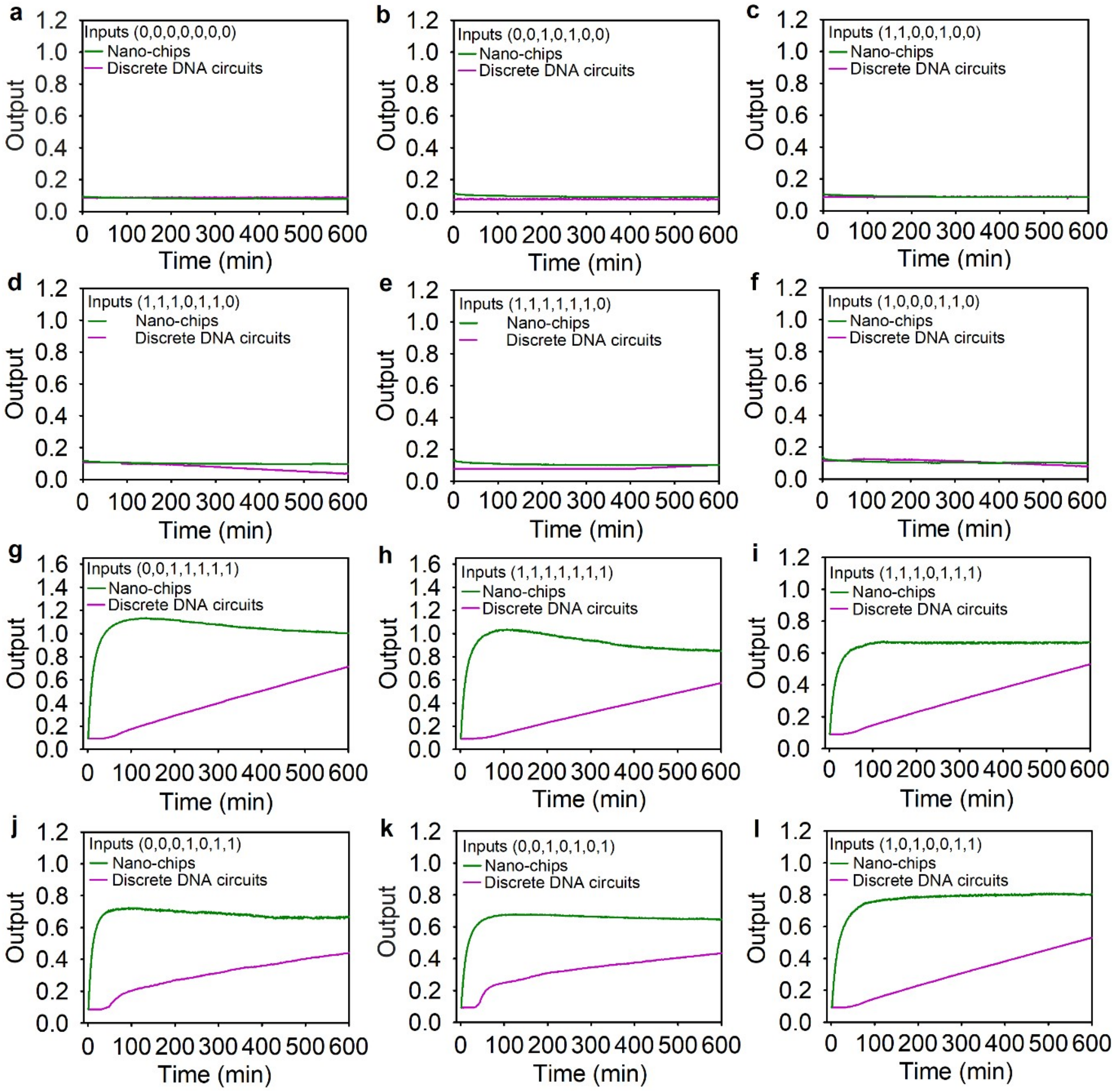

Figure S22. Time-dependent FAM fluorescence signals (Output S_1) of the OR-AND circuit in nano-chip or discrete DNA circuit response to different inputs (the input order was miR-122a, -15a, -17, -30a, miR-124a, -143 and -10b). (a) The fluorescence signals of Inputs (0, 0, 0, 0, 0, 0, 0); (b) the fluorescence signals of Inputs (0, 0, 1,0, 1,0, 0); (c) the fluorescence signals of Inputs (1, 1,0, 0, 1, 0, 0); (d) the fluorescence signals of Inputs (1, 1, 1, 0, 1, 1, 0); (e) the fluorescence signals of Inputs (1, 1, 1, 1, 1, 1, 0); (f) the fluorescence signals of Inputs (1, 0, 0, 0, 1, 1, 0); (g) the fluorescence signals of Inputs (0, 0, 1, 1, 1, 1, 1), the kinetic rate constant was 0.0020 min^{-1} and 0.0968 min^{-1} for the diffusible system and nano-chip, respectively; (h) the fluorescence signals of Inputs (1, 1, 1, 1, 1, 1, 1), the kinetic rate constant was 0.0025 min^{-1} and 0.0879 min^{-1} for the diffusible system and nano-chip, respectively; (i) the fluorescence signals of Inputs (1, 1, 1, 0, 1, 1, 1), the kinetic rate constant was 0.0013 min^{-1} and 0.0303 min^{-1} for the diffusible system and nano-chip, respectively; (j) the fluorescence signals of Inputs (0, 0, 0, 1, 0, 1, 1), the kinetic rate constant was 0.0010 min^{-1} and 0.0588 min^{-1} for the diffusible system and nano-chip, respectively; (k) the fluorescence signals of Inputs (0, 0, 1, 0, 1, 0, 1), the kinetic rate constant was 0.0008 min^{-1} and 0.0344 min^{-1} for the diffusible system and nano-chip, respectively; (l) the fluorescence signals of Inputs (1, 0, 1, 0, 0, 1, 1), the kinetic rate constant was 0.0013 min^{-1} and 0.0383 min^{-1} for the diffusible system and nano-chip, respectively. Green lines represented the fluorescence signals of nano-chip responsed to inputs, purple lines represented the fluorescence signals of discrete DNA circuit responsed to inputs.

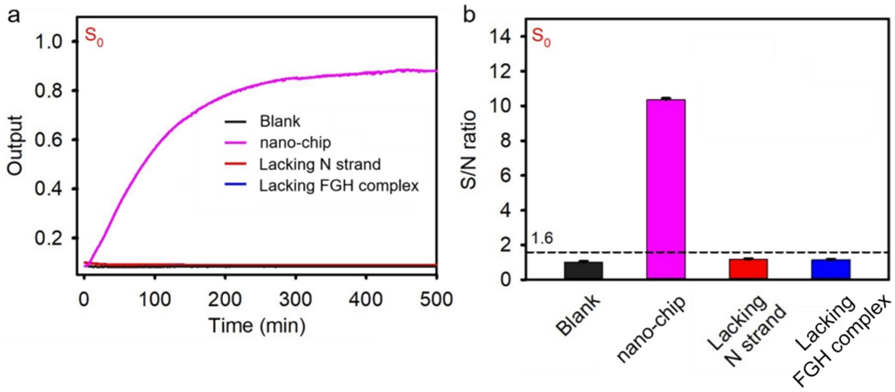

Figure S23. The performance of nano-chip loading with AND-OR-NOT-AND circuit and two control nano-chips lacking N strand or the FGH complex in responding to inputs (miR-122a, miR-15a, miR-17).

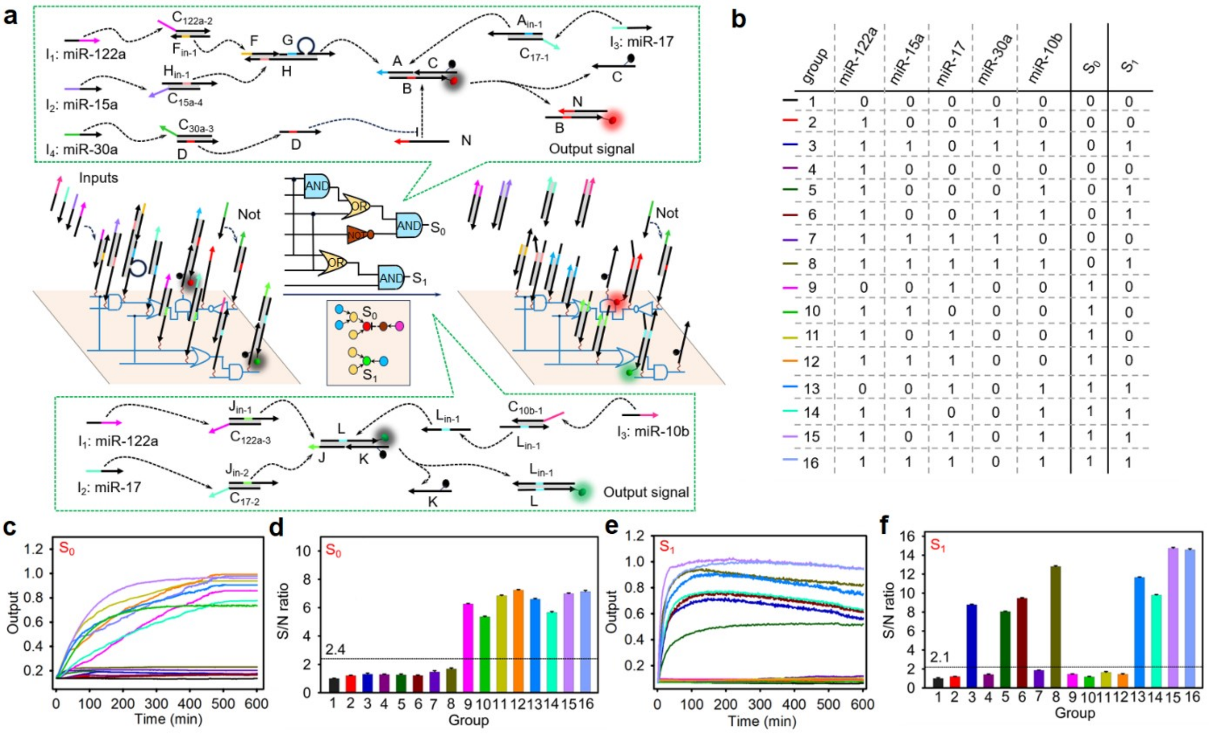

group	miR-122a	miR-15a	miR-17	miR-30a	miR-10b	S_0	S_1
1	0	0	0	0	0	0	0
2	1	0	0	1	0	0	0
3	1	1	0	1	1	0	1
4	1	0	0	0	0	0	0
5	1	0	0	0	1	0	1
6	1	0	0	1	1	0	1
7	1	1	1	1	0	0	0
8	1	1	1	1	1	0	1
9	0	0	1	0	0	1	0
10	1	1	0	0	0	1	0
11	1	0	1	0	0	1	0
12	1	1	1	0	0	1	0
13	0	0	1	0	1	1	1
14	1	1	0	0	1	1	1
15	1	0	1	0	1	1	1
16	1	1	1	0	1	1	1

Figure S24. Design and performance of the scalable cross-selective nano-chip with 11 addressable dual-rail logical components for five-inputs multi-levels logic cascading and parallel biocomputing. (a) Illustration of its operating mechanism. Brief, in the AND-OR-NOT-AND circuit, two duplex DNA complex $F_{in-1}C_{122a-2}$ and $H_{in-1}C_{15a-4}$ converted the input microRNAs (-122a and -15a) to single-stranded DNA (ssDNA, F_{in-1}, H_{in-1}), and the latter hybridized with the triple-stranded DNA complex FGH to release ssDNA G. Input miR-17 displaced ssDNA A_{in-1} from duplex DNA complex $A_{in-1}C_{17-1}$. One of ssDNA G or ssDNA A_{in-1} hybridized with the triple-stranded DNA complex ABC to release duplex DNA complex BC. Then, ssDNA N liberated ssDNA C and produced duplex DNA complex BN, lighting up the fluorescence signal S_0. Once in the presence of miR-30a, duplex DNA complex DC_{30a-3} converted it to the ssDNA D, which could preferentially hybridize with ssDNA N to prevent subsequent displacement reaction. In the OR-AND circuit, one or both two input miRNAs displaced ssDNA J_{in-1} or J_{in-2} from duplex DNA complex $J_{in-1}C_{122a-3}$ or $J_{in-2}C_{17-2}$, and the latter hybridized with the triple-stranded DNA complex JKL to release duplex DNA complex KL. Input miR-10b displaced ssDNA L_{in-1} from duplex DNA complex $L_{in-1}C_{10b-1}$. Then, ssDNA L_{in-1} liberated ssDNA K and produced duplex DNA complex LL_{in-1}, lighting up the fluorescence signal S_1. The top view of the localized circuit on origami showed the three basic logic gates as color-coded circles (AND: blue; OR: Yellow; NOT: brown; the assistant component of NOT gate: purple red; output S_0: red; output S_1: green). The segments with same color are complementary sequences of toehold domain. The more detailed descriptions about wiring instructions were given in Figure S44b. (b) The truth value table of the parallel nano-chip; (c) time-dependent reactivity of AND-OR-NOT-AND circuit corresponding to different inputs; (d) S/N ratio obtained from (c) with fluorescence intensity at 500 min; (e) time-dependent reactivity of OR-AND circuit corresponding to different inputs; (f) S/N ratio obtained from (e) with fluorescence intensity at 200 min.

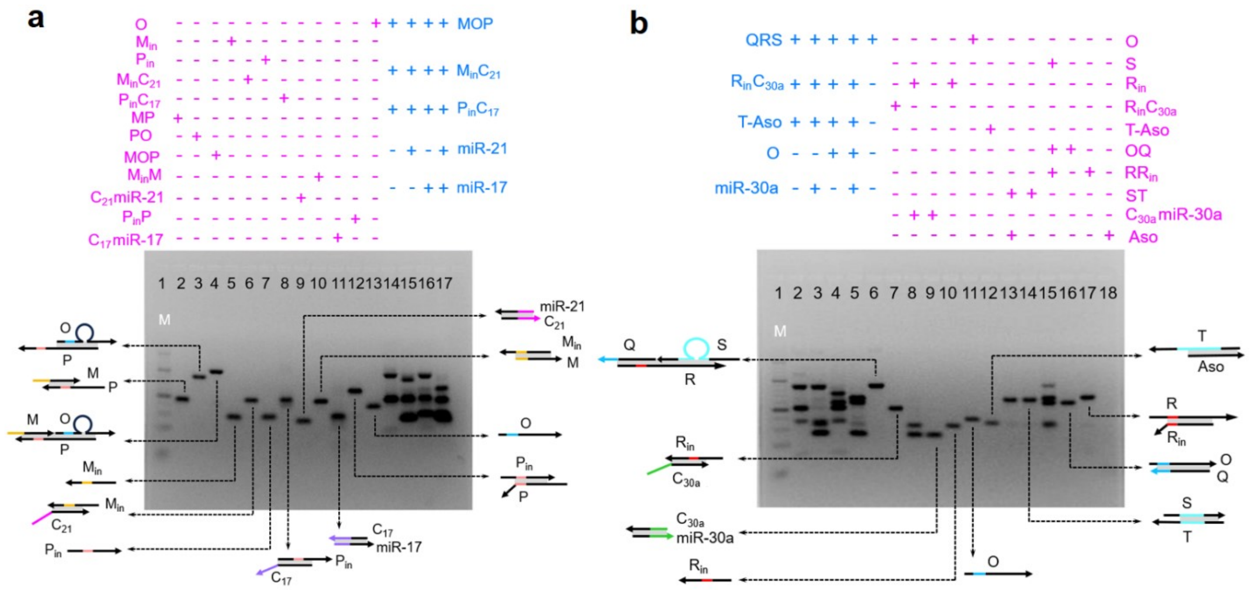

Figure S25. (a) Agarose gel electrophoresis analysis of the toehold mediated strand displacement reaction between inputs (miRNA-21 and miRNA-17) and the functional DNA units, including two DNA duplex $M_{in}C_{21}$, $P_{in}C_{17}$ and the triple-stranded complex MOP. Lane 2-13 exhibited the products of the corresponding ssDNA and DNA duplexes marked in purple-red words. Lane 14-17 showed the products of the reactions between inputs and the functional DNA units marked in blue words. (b) Agarose gel electrophoresis analysis of the toehold mediated strand displacement reaction between input miRNA-30a and the functional DNA units, including ssDNA O, two DNA duplex $R_{in}C_{30a}$, T-Aso and the triple-stranded complex QRS. Lane 2-6 showed the products of the reactions between inputs and the functional DNA units marked in blue words. Lane 7-18 exhibited the products of the corresponding ssDNA and DNA duplexes marked in purple-red words.

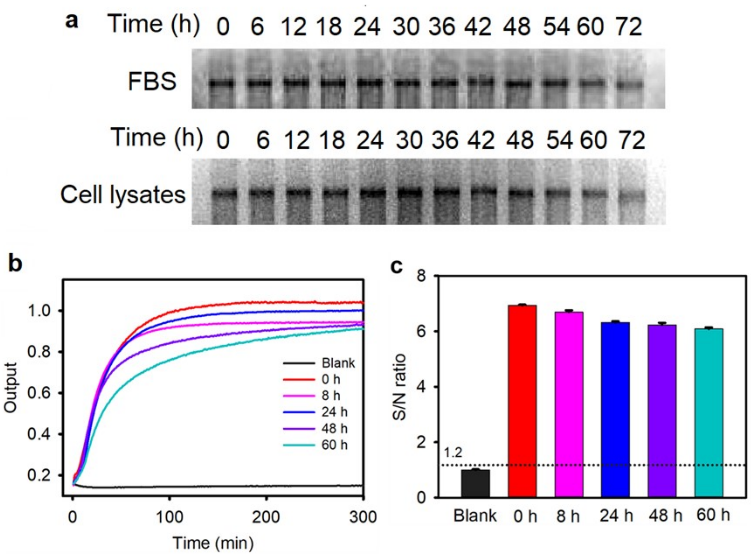

Figure S26. Study on the stability of the nano-chip. (a) Degradation analysis of nano-chip in DMEM medium containing 10% FBS (up) and cell lysates (down) at 37°C for different times. (b) the real-time kinetic monitoring of nano-chip (integrated with a three-input AND logic circuit) in responding to inputs (miR-21, miR-17 and miR-30a), the nano-chip was pre-incubated with cell lysates for different times (0, 8, 24, 48 and 60 h). (c) S/N ratio obtained from (b) with fluorescence intensity at 200 min.

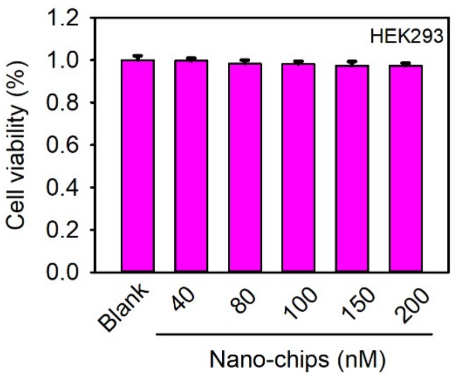

Figure S27. Cell viability of HEK293 cells after incubated with different concentrations of nano-chip (0, 40, 80, 100, 150 and 200 nM) at 37°C for 48 h.

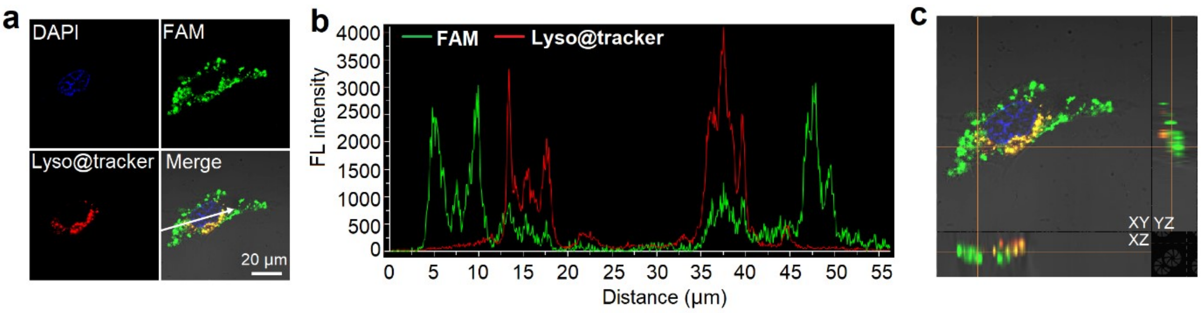

Figure S28. Endo-lysosomal release of nano-chip in HeLa cells. (a) CLSM images of HeLa cells after incubation with 40 nM FAM-modified nano-chip for 4 h followed by staining with 75 nM Lyso@Tracker Red and 10 μg/mL Hoechst 33342 for 25 min, scale bar: 20 μm. (b) The fluorescence intensity curves of Lyso@Tracker and FAM in the regions marked straight white line of (a). (c) Confocal z-stacks images with a cross-section in xy, xz and yz for localization of nano-chip in HeLa cells.

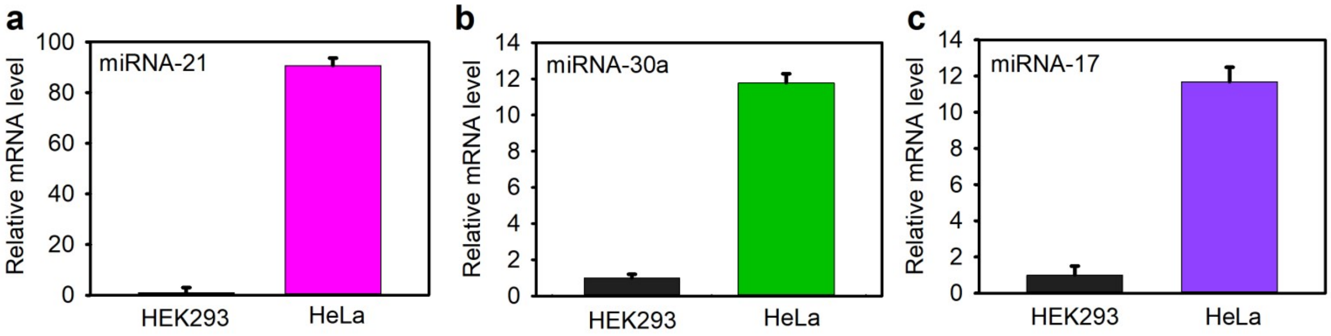

Figure S29. The relative expression levels of miRNAs in HEK293 and HeLa cells by using quantitative reverse transcription-PCR (qRT-PCR) analysis method. (a) miRNA-21, (b) miRNA-30a and (c) miRNA-17.

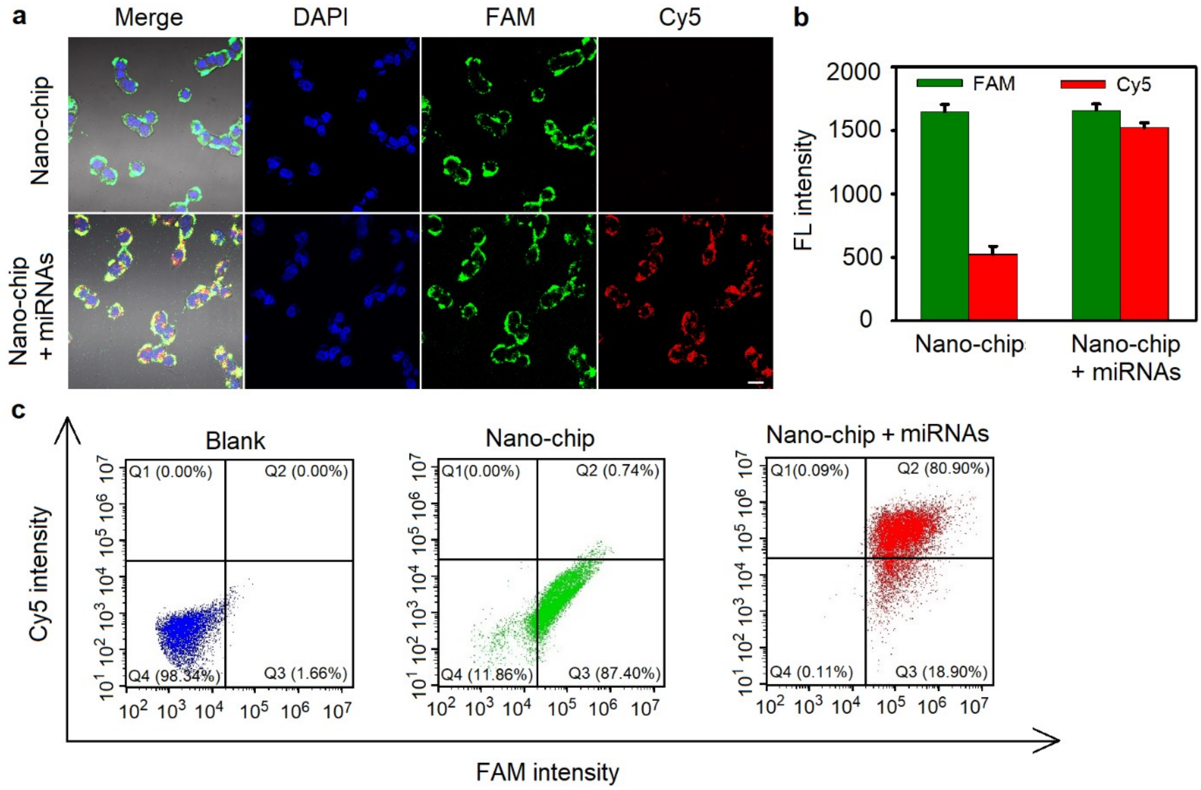

Figure S30. The performance of nano-chip in HEK293 cell. (a) CLSM images of HEK293 cells after different treatments, scale bar: 20 μm. (b) The relevant statistical histogram analysis of the CLSM images. (c) The corresponding flow cytometry analysis for HEK293 cells after different treatments.

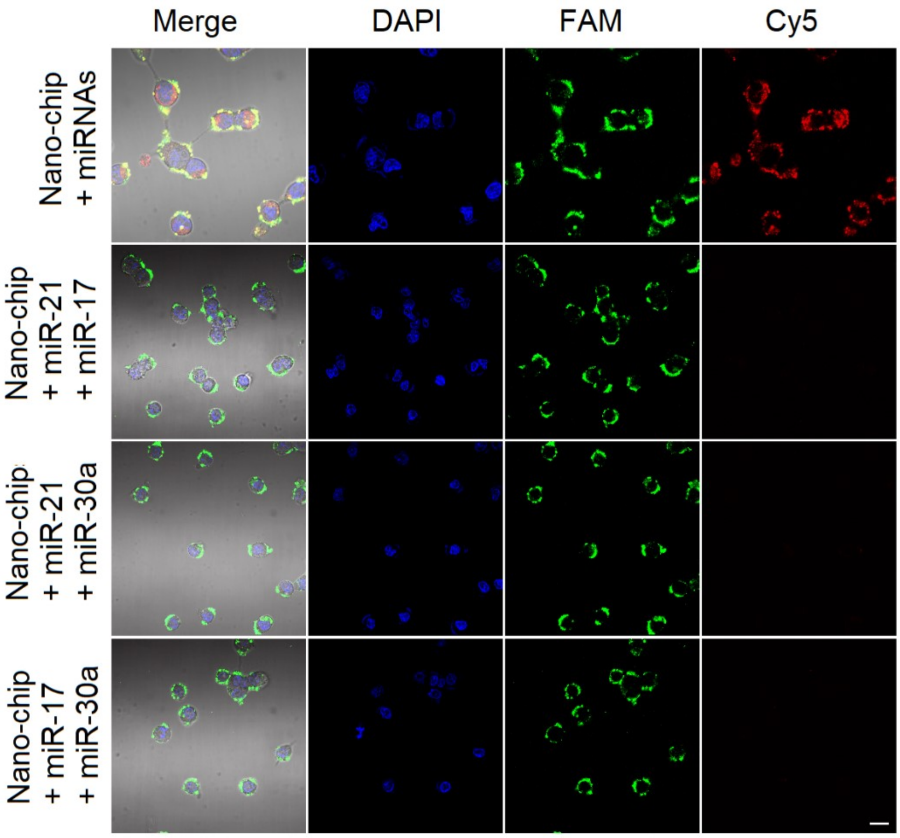

Figure S31. CLSM images of HEK293 cells after pre-treated with different miRNA mimics following incubation with nano-chip, scale bar: 20 μm.

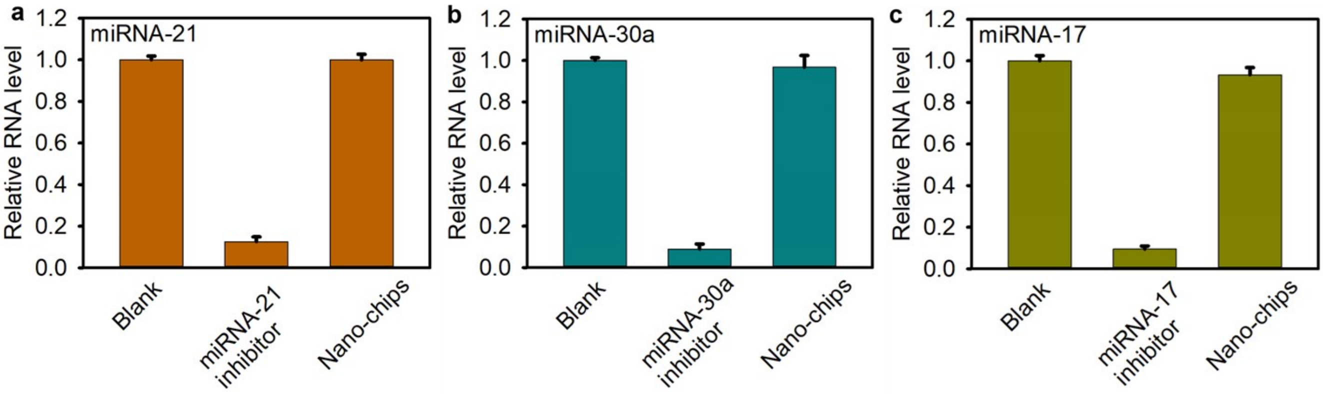

Figure S32. The relative expression levels of miRNAs in HeLa cells after treated with different miRNA inhibitors. (a) miRNA-21, (b) miRNA-30a and (c) miRNA-17.

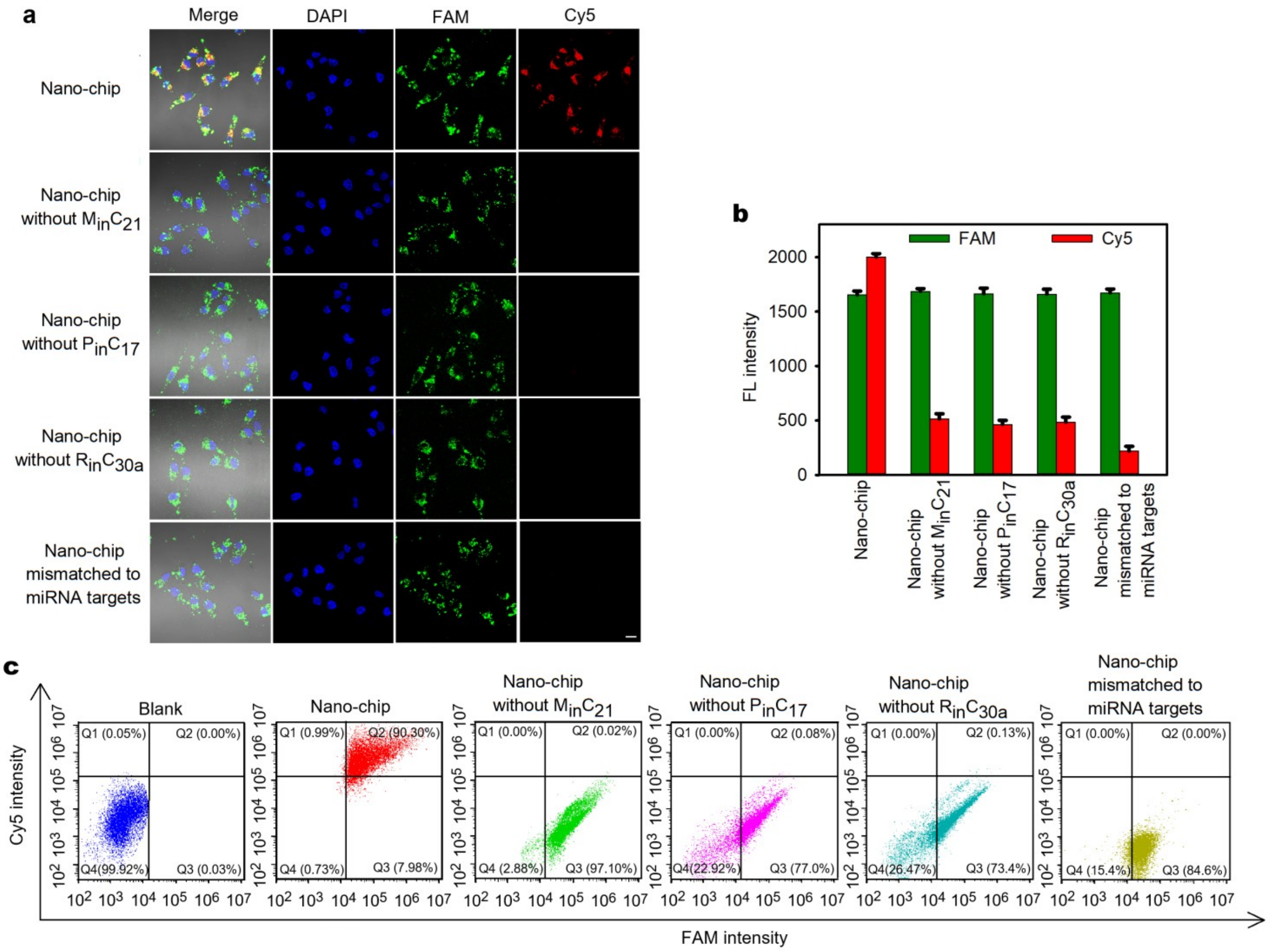

Figure S33. The performance of different nano-chips in Hela cell. (a) CLSM images of Hela cells after incubated with different nano-chips, scale bar: 20 μm. (b) The relevant statistical histogram analysis of the CLSM images. (c) The corresponding flow cytometry analysis for Hela cells after different treatments. In the Nano-chip mismatched to miRNA targets, this mismatched control nano-chip was designed to have the same structural configuration as the fully complementary AND logic gate, but with 2 base pair mismatches in the miRNA toehold-recognized regions.

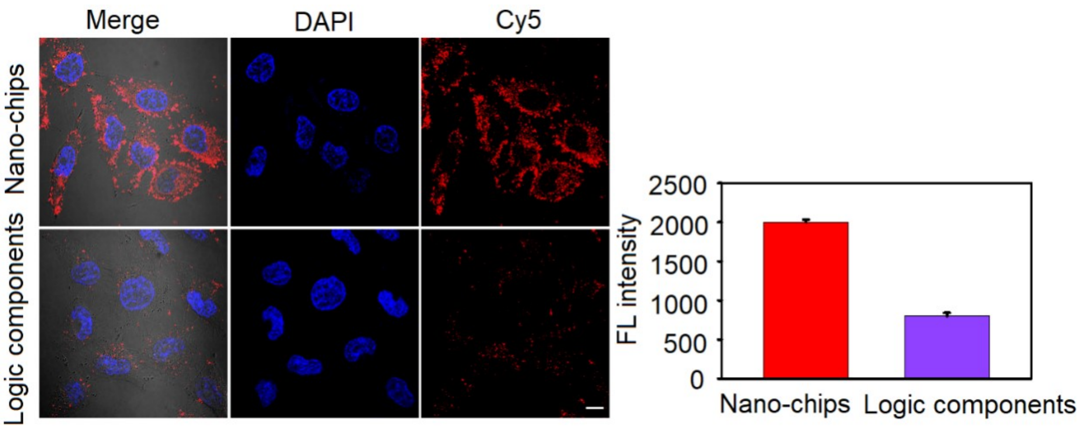

Figure S34. (a) CLSM images of Hela cells after different treatments. Up: nano-chip. Down: discrete DNA logic components, scale bar: 20 μm. (b) The relevant statistical histogram analysis of the CLSM images.

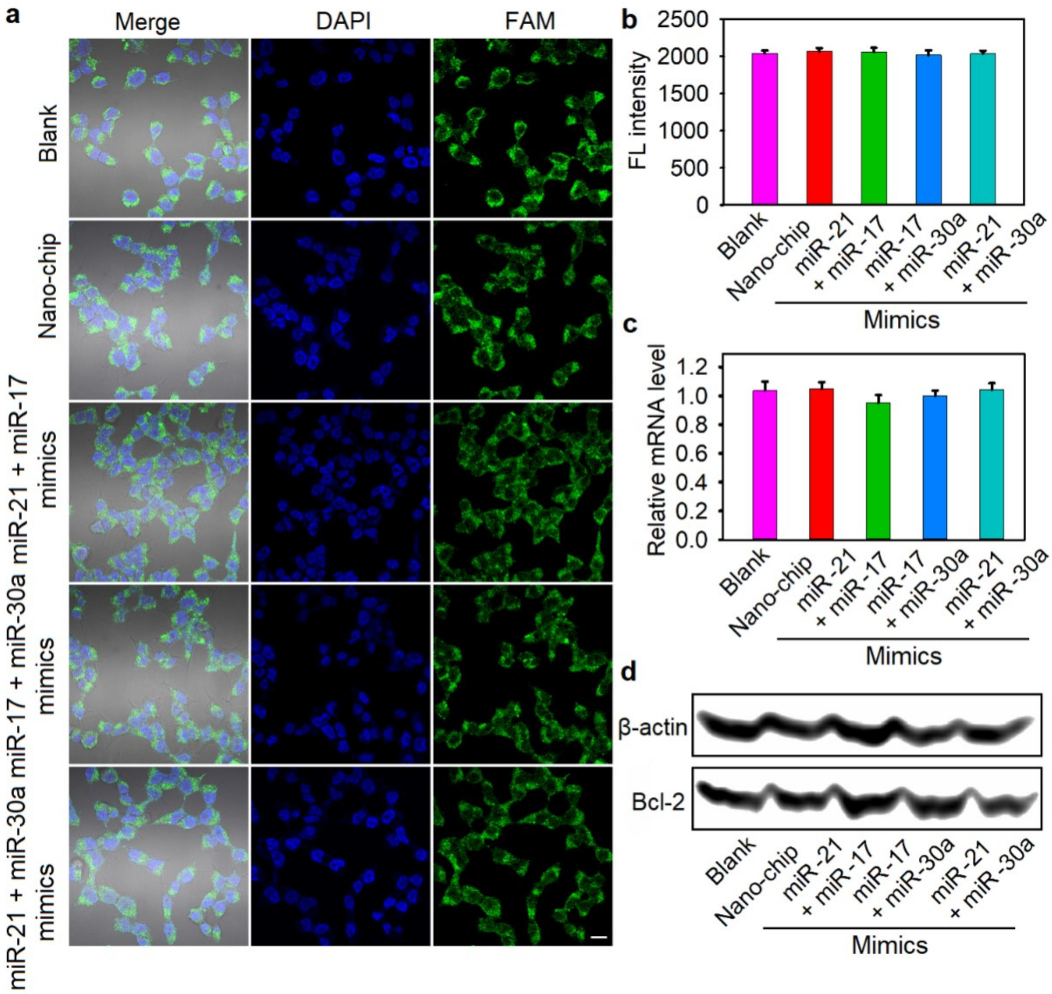

Figure S35. (a) Immuno-fluorescence imaging of Bcl2 protein in HEK293 cells after different treatments, scale bar: 20 μm. (b) The relevant statistical histogram analysis of the Immuno-fluorescence imaging. (c) The relative expression levels of Bcl2 mRNA in HEK293 cells after different treatments. (d) Western blotting assay of Bcl2 protein from cell lysate from HEK293 cells after different treatments.

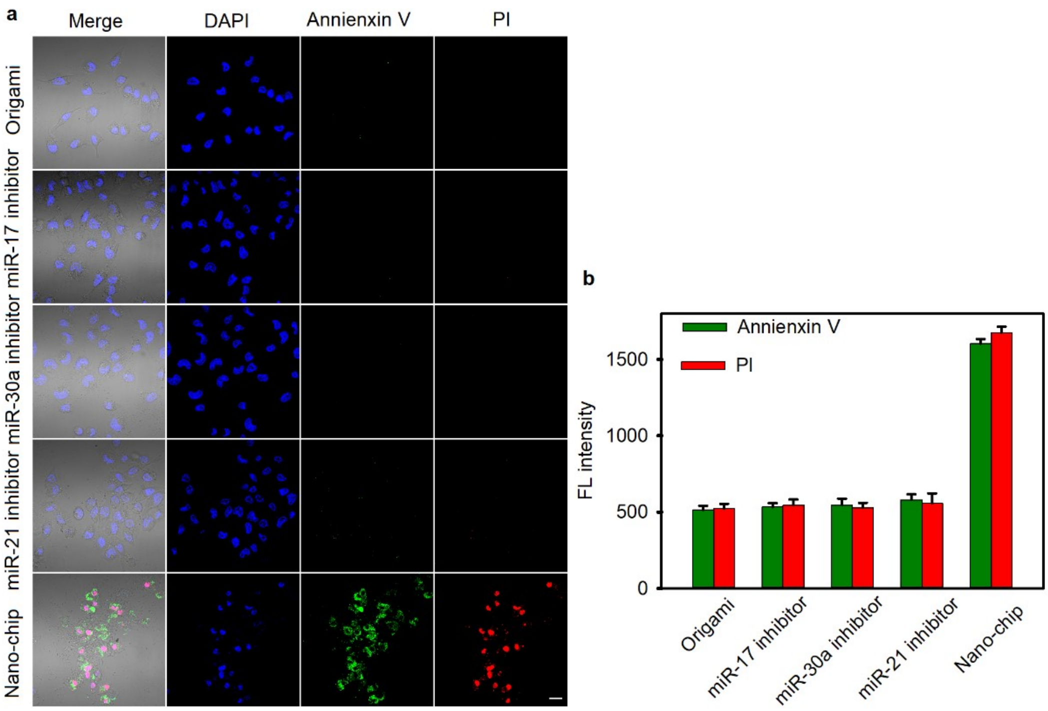

Figure S36. (a) The CLSM images of Hela cells after different treatments by using Annexin V and the PI dead cell apoptosis kit, scale bar: 20 μm. (b) The relevant statistical histogram analysis of the CLSM images.

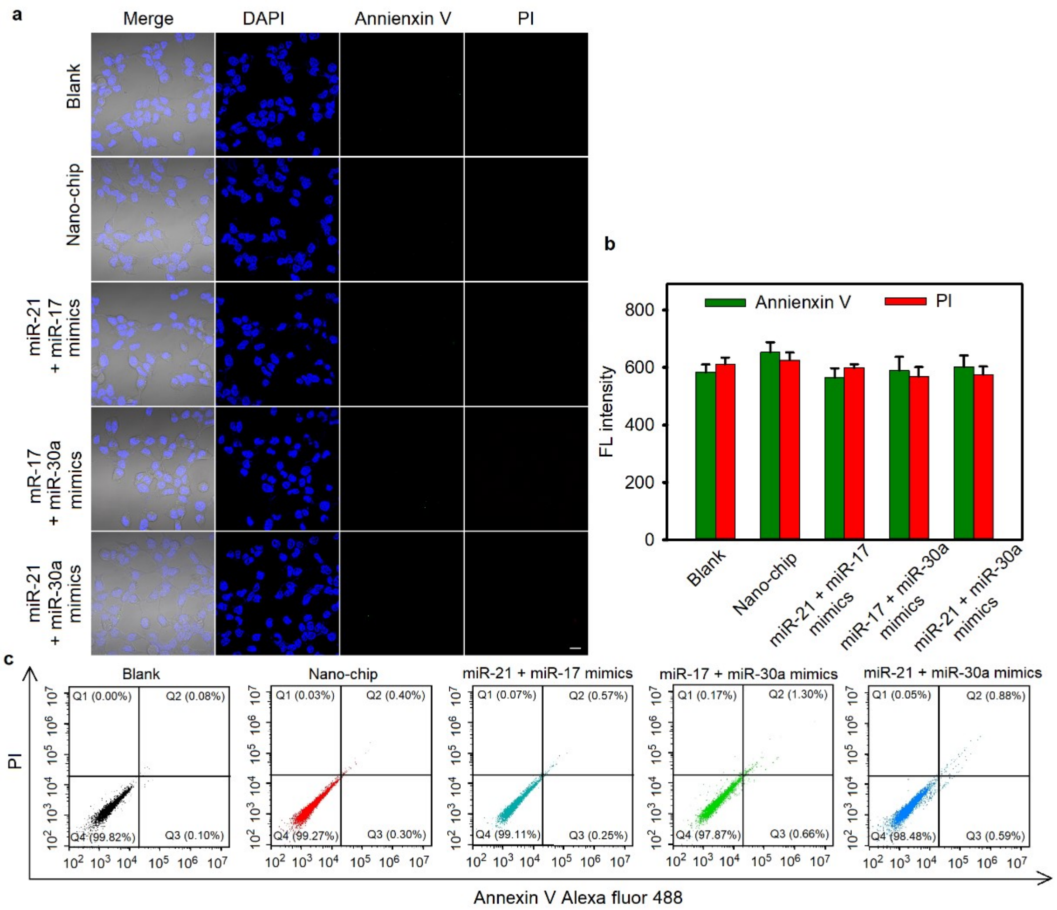

Figure S37. (a) The CLSM images of HEK293 cells after different treatments by using Annexin V and the PI dead cell apoptosis kit, scale bar: 20 μm. (b) The relevant statistical histogram analysis of the CLSM images. (e) Flow cytometry assay of analyzing apoptosis in HEK293 cells after different treatments by using Annexin V and the PI dead cell apoptosis kit.

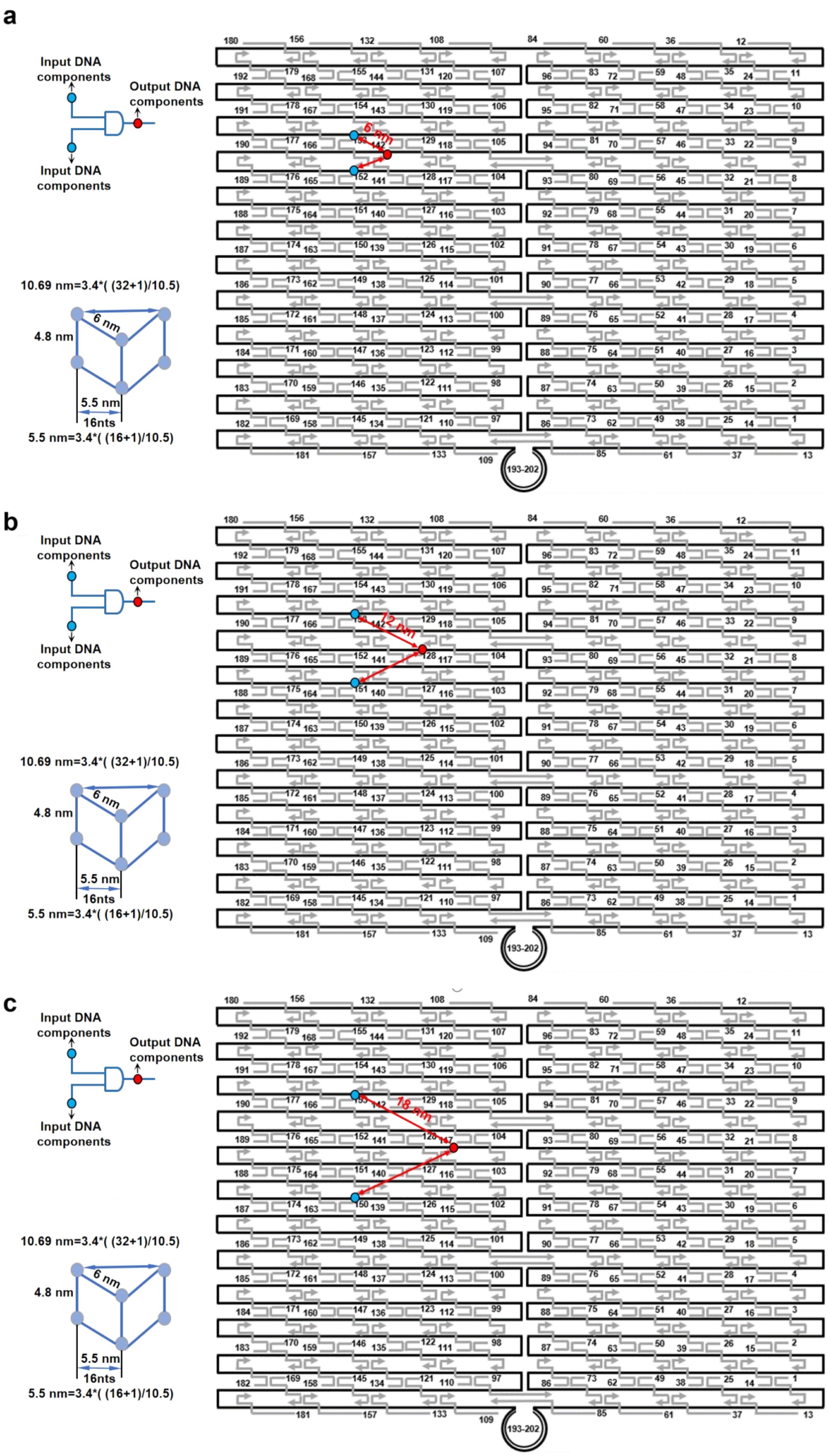

Figure S38. The wiring instructions of the AND logic gate with different assembly distance between Input and Output DNA components on DNA origami. (a) 6 nm; (b) 12 nm; (c) 18 nm.

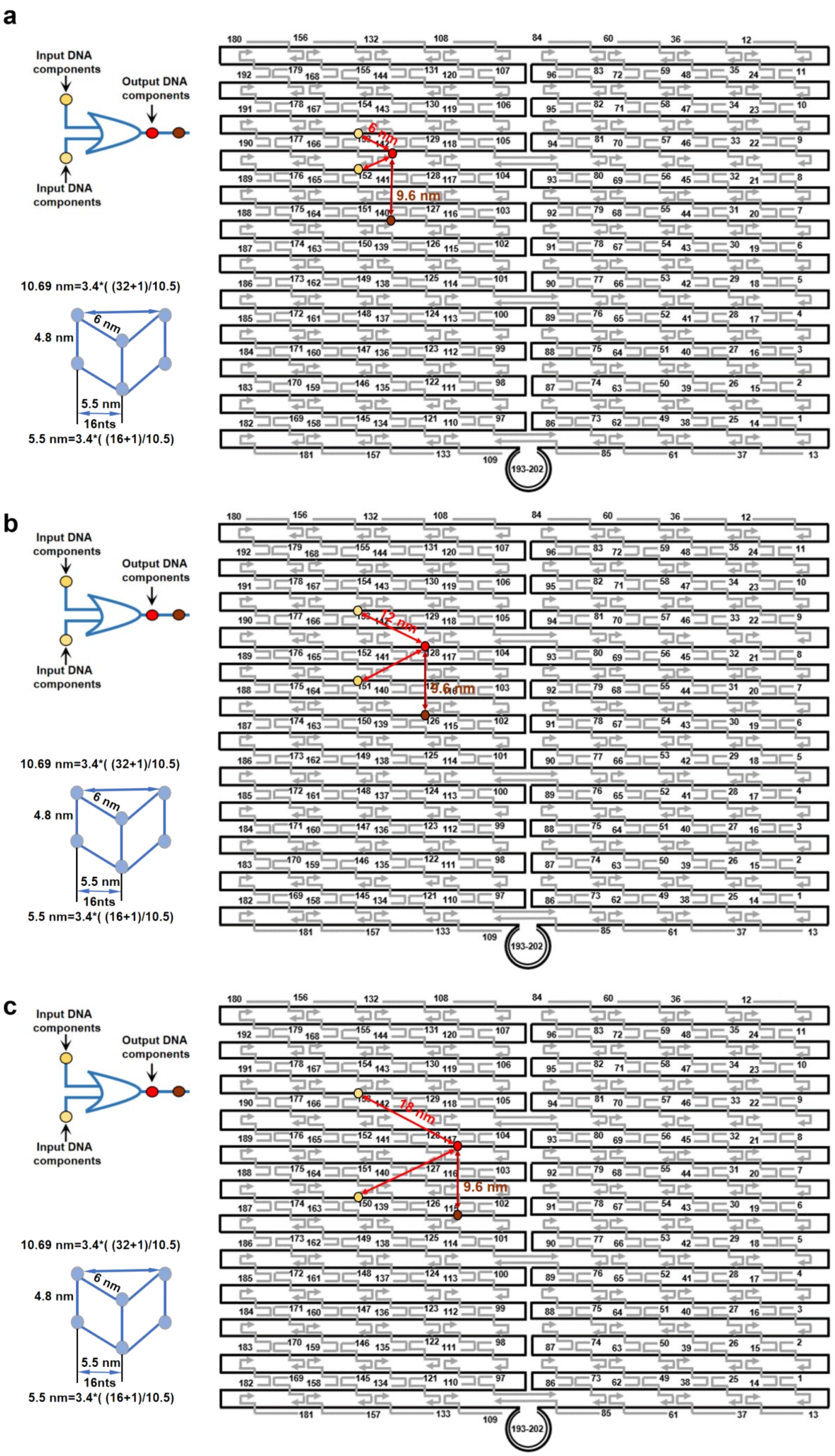

Figure S39. The wiring instructions of the OR logic gate with different assembly distance between Input and Output DNA components on DNA origami. (a) 6 nm; (b) 12 nm; (c) 18 nm.

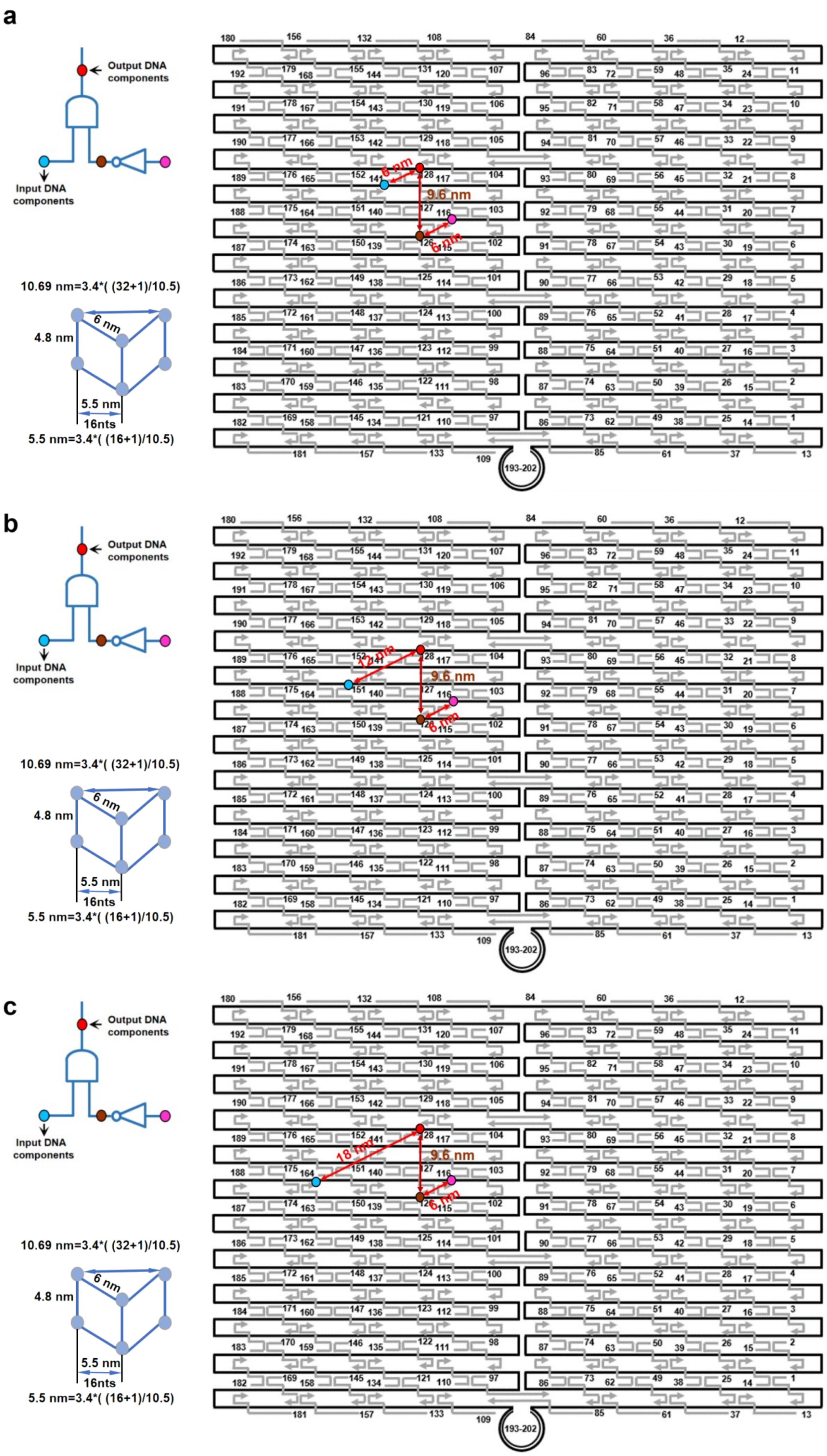

Figure S40. The wiring instructions of the AND-NOT logic gate with different assembly distance between Input and Output DNA components on DNA origami. (a) 6 nm; (b) 12 nm; (c) 18 nm.

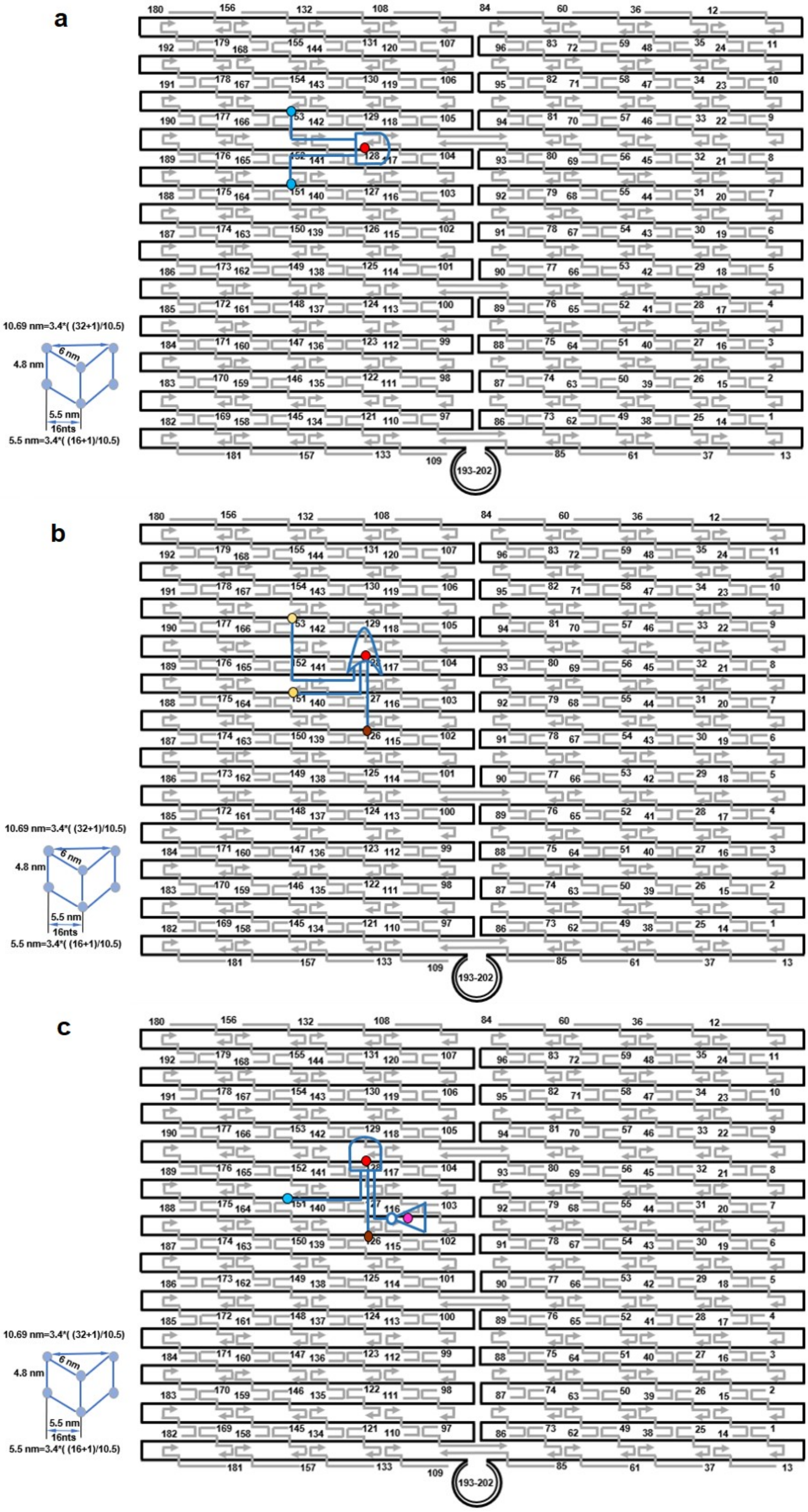

Figure S41. The wiring instructions of three basic logic gates on DNA origami. (a) AND gate; (b) OR gate; (c) NOT-AND gate.

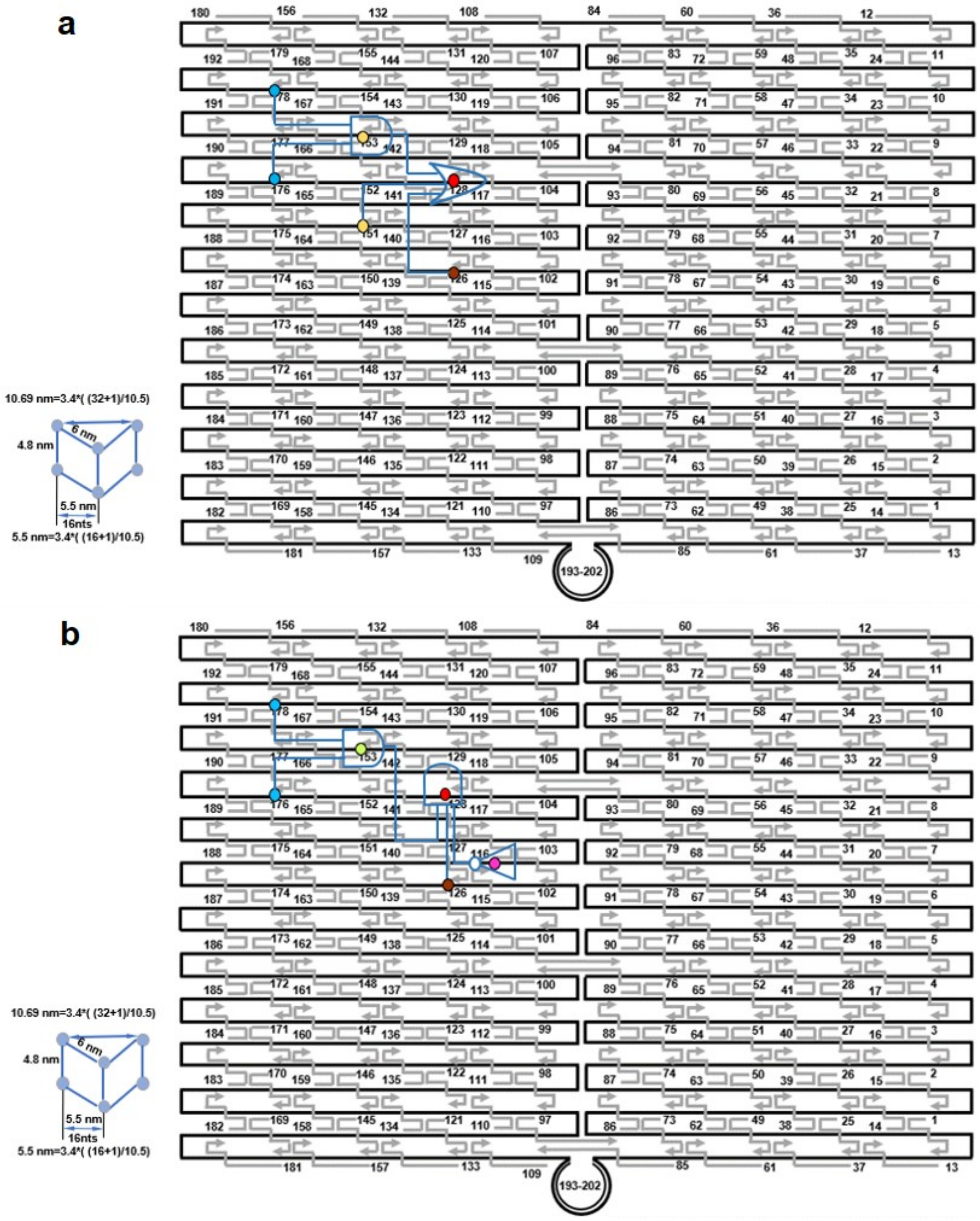

Figure S42. The wiring instructions of complex DNA cascade circuits on the basis of the basic AND nano-chip. (a) AND-OR nano-chip; (b) AND-NOT-AND nano-chip.

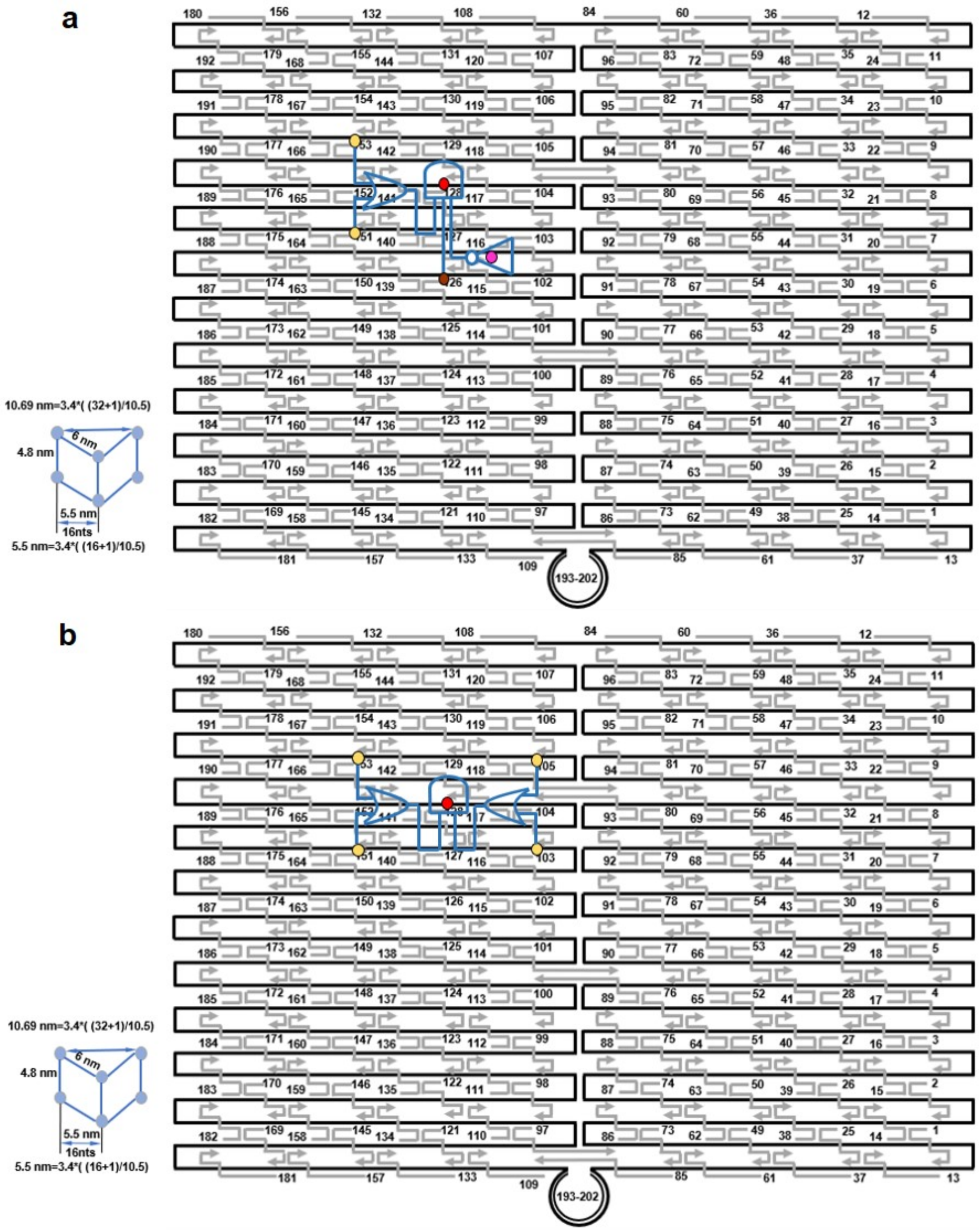

Figure S43. The wiring instructions of complex DNA cascade circuits on the basis of the basic OR nano-chip. (a) OR-NOT-AND nano-chip; (b) OR-OR-AND nano-chip.

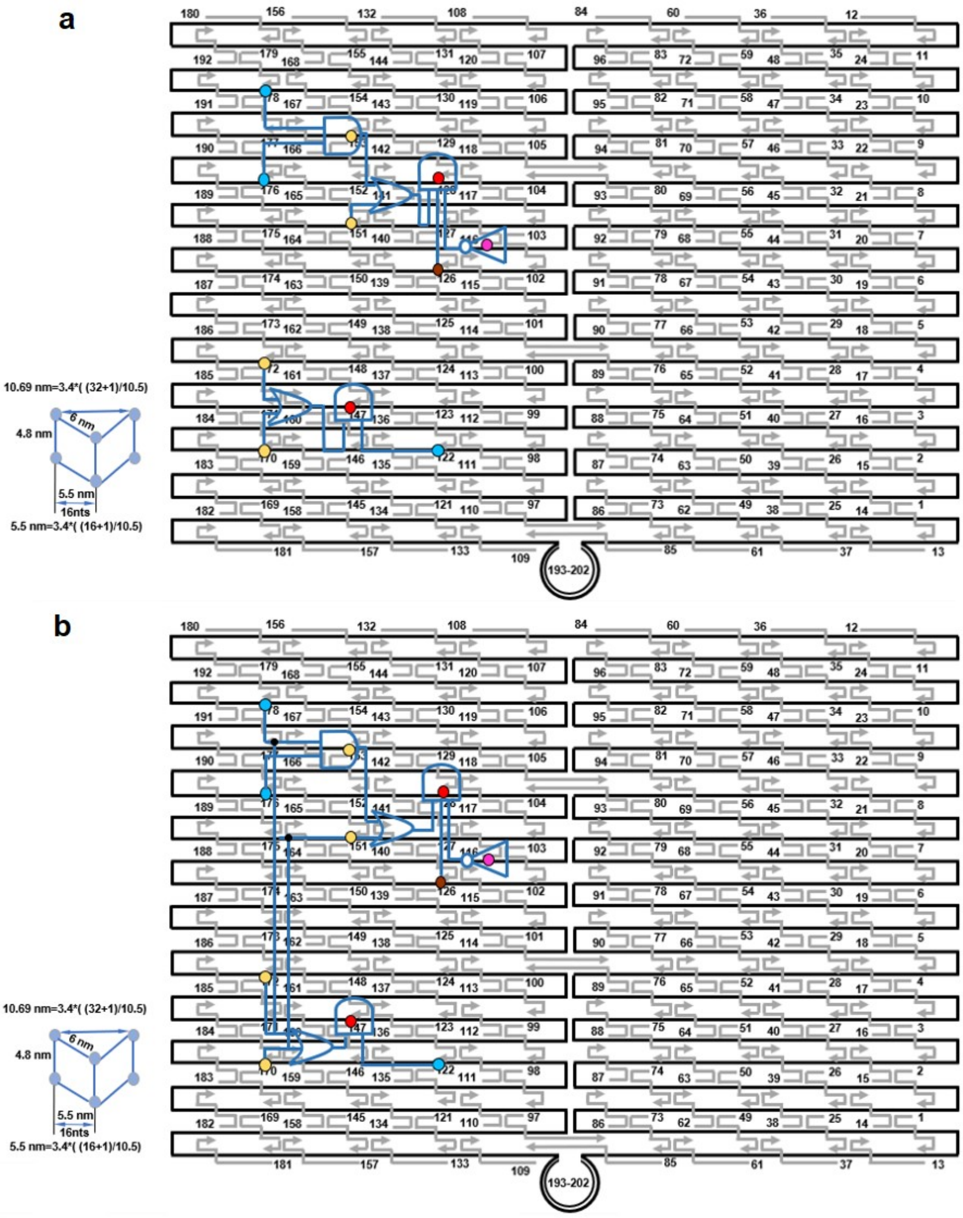

Figure S44. The wiring instructions of the scalable nano-chip with 11 addressable dual-rail logical components for multi-levels logic cascading and parallel biocomputing. (a) Seven-inputs with non-cross-selective nano-chip; (b) five-inputs with cross-selective nano-chip.

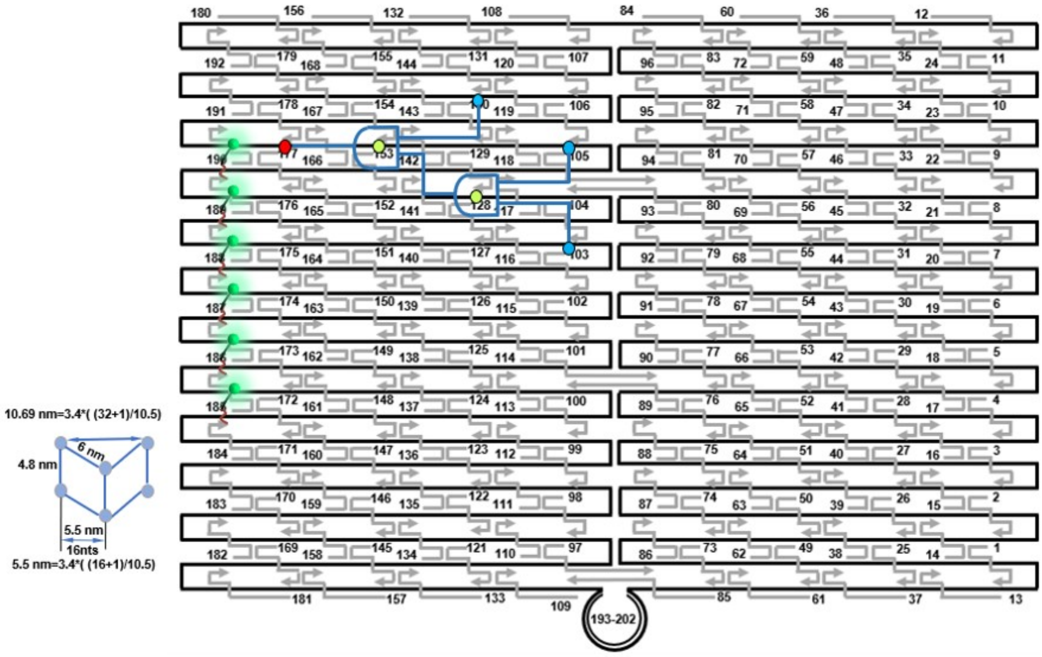

Figure S45. The wiring instructions of the three layers of cascade logic units on the nano-chip for intracellular molecular biocomputing.

Table S1. Sequence of oligonucleotides used in modular logic units.

Name	Sequence (5'-3')
A	GTAGTATTGTACTCTGTCACGATAG
B	GTGACAGAGTACAATACTACATGACTTCGACACTACAGAT-Cy5
C	TTCACCTGATAGTACCTTT(BHQ2)ATCTGTAGTGTCGAAGTCAT

A_{in-1}	AGTGCAGGTAGCTATCGTGACAGAGTACAATACTACTTTACATCACCTATCGCTA
B_{in-1}	ATCCATCTAGGATATCTTTTGTAGTGTCGAAGTCATGTAGTTGTAAACATCC
C_{17-1}	CACGATAGCTACCTGCACTGTAAGCACTTTG
miR-17	CAAAGTGCTTACAGTGCAGGTAG
C_{30a-1}	CTTCCAGTCGAGGATGTTTACAACTACATG
miR-30a	TGTAAACATCCTCGACTGGAAG
C-staple(128)	GGTACTATCAGGTGAAGCGTCTTTATAGCAGCCTTTACAGTAGCTATC
A_{in-1}-staple(154)	GAAGAAAGCGAAAGGAGCGGGCGCTAGGGCGCTAGCGATAGGTGATGT
B_{in-1}-staple(151)	GATATCCTAGATGGATATTTTATCAACAGGGAAGCGCATTATAATAAG
A_{in-2}	CGACTGGAAGCTATCGTGACAGAGTACAATACTACTTTCTATAGGATCTACCTA
C_{30a-2}	CACGATAGCTTCCAGTCGAGGATGTTTACA
N	CTCATGTCATCTCGTCTTACTGTAGTGTCGAAGTCATGTAGT
A_{in-2} staple(152)	TCTGTCCAAAATAAGAATAAACACAAATATATTAGGTAGATCCTATAG
N-staple(126)	GACGAGATGACATGAGACATCACCGTAAGAATACGTGGTTAATGCG
C_{30a-3}	GTCATGTAGTCTTCCAGTCGAGGATGTTTACA
D	CTCGACTGGAAGACTACATGACTTCGACACTTTTTCTATAGGATCTACCTA
D-staple(115)	ATTTTTGAAATTGTAAACGTTAATTAGCCAGCTAGGTAGATCCTATAG
A_{in-1}-staple(152)	TCTGTCCAAAATAAGAATAAACACAAATATATTAGCGATAGGTGATGT
C_{122a-1}	CACGATAGCAAACACCATTGTCACACTCCA
A_{in-3}	AATGGTGTTTGCTATCGTGACAGAGTACAATACTACTTTACATCACCTATCGCTA
miR-122a	TGGAGTGTGACAATGGTGTTTG
B_{in-2}	ATCCATCTAGGATATCTTTTGTAGTGTCGAAGTCATGTAGTTAGCAGCACA
C_{15a-1}	CACAAACCATTATGTGCTGCTAACTACATG
miR-15a	TAGCAGCACATAATGGTTTGTG
A_{in-4}	AATGGTTTGTGCTATCGTGACAGAGTACAATACTACTTTCTATAGGATCTACCTA
C_{15a-2}	CACGATAGCACAAACCATTATGTGCTGCTA
E	AATGGTTTGTGACTACATGACTTCGACACTTTTTCTATAGGATCTACCTA
C_{15a-3}	GTCATGTAGTCACAAACCATTATGTGCTGCTA
F	CACCTCTGAATGACGGATACTAGATGTGCT
G	CTGACAGATCTATCGTGACAGAGTACAATACTACCCTTCACCAGTCTTACCTTTTCGATTCCAGTTCACAG
H	GGTAAGACTGGTGAAGGCACGATAGATCTGTCAGAGCACATCTAGTATCCGTCATTCAG
C_{122a-2}	CAAACACCATTGTCACACTCCACACCTCTG
F_{in-1}	CCTTCGTATCGCATAGTTTAGCACATCTAGTATCCGTCATTCAGAGGTGTGGAGTGTG
H_{in-1}	ATGGTTTGTGGTGCTCTGACAGATCTATCGTGCCTTCACCAGTCATTTATCCGATTCGCATGAC
C_{15a-4}	CAGAGCACCACAAACCATTATGTGCTGCTA
G-staple(154)	GAAGAAAGCGAAAGGAGCGGGCGCTAGGGCGCCTGTGAACTGGAATCG
F_{in-1}-staple(178)	CTATGCGATACGAAGGGGTTGAGGCTTTTCATAATCAAAAAGGCCG
H_{in-1}-staple(177)	TCATGGAATGGATTATTTACATAACACCGCGTCATGCGAATCGGAT
A_{in-5}	GGTGAATGCCCTATCGTGACAGAGTACAATACTACTTTCTATAGGATCTACGTA
C_{124a-1}	CACGATAGGGCATTCACCGCGTGCCTTA
miR-124a	TAAGGCACGCGGTGAATGCC
B_{in-2}	ACTCTTCCATTCGCTATTTTGTAGTGTCGAAGTCATGTAGTTAGCAGCACA
C_{15a-1}	CACAAACCATTATGTGCTGCTAACTACATG
A_{in-5}-staple(152)	TCTGTCCAAAATAAGAATAAACACAAATATATTACGTAGATCCTATAG
B_{in-1}-staple(105)	GACGAGATGACATGAGTTTCATCATCGCACTCCAGCCAGCTTCGCTAT
B_{in-2}-staple((103)	TAGCGAATGGAAGAGTTTCTGTATGAGGTGAATTTCTTAAGGCCGCTT
J_{in-1}	ATGGTGTTTGTCGTCGTCTAAGCCTATCCTTTCCGAGCTATTTTCTTACGCAATCCTTCC
C_{122a-3}	GACGACGACAAACACCATTGTCACACTCCA
J_{in-2}	GTGCAGGTAGTCGTCGTCTAAGCCTATCCTTTCCGAGCTATTTTCAGTGCATCGTAGTCA
C_{17-2}	GACGACGACTACCTGCACTGTAAGCACTTTG
L_{in-1}	CATAGCACATACCGAGTTTTCAGTGGATCGTGAACAGTATCAGTAGCTCCCTGTAGAA
C_{10b-1}	ACACAAATTCGGTTCTACAGGGAGCTACTG
miR-10b	CCCTGTAGAACCGAATTTGTGT
L	GTCTAAGCCTATCCTTTCCGAGCTACTGATACTGTTCACGATCCACTGAT-FAM
J	TAGCTCGGAAAGGATAGGCTTAGACGACGA
K	CTCTAGTTCGTCTGCCTTTTAT(Dabcyl)CAGTGGATCGTGAACAGTATCAG
J_{in-1}-staple(173)	ATGAATATACATTTAACAATTTCACCTTAGAAGGAAGGATTGCGTAAG
J_{in-2}-staple(171)	TAATTACTAGTACCGACAAAAGGTAAATAATATGACTACGATGCACTG
K-staple(147)	GGCAGACGAACTAGAGCGTATAATAAACAGGAGGCCGAAAAAACGC
L_{in-1}-staple(122)	CTCGGTATGTGCTATGTTGAATATCATTTCAATTACCTATTTATCA
J_{in-3}	GGTGAATGCCTCGTCGTCTAAGCCTATCCTTTCCGAGCTATTTTCTTACGCAATCCTTCC
C_{124a-2}	GACGACGAGGCATTCACCGCGTGCCTTA
J_{in-4}	TGTAGCTCATCGTCGTCTAAGCCTATCCTTTCCGAGCTATTTTCAGTGCATCGTAGTCA
C_{143-1}	GACGACGATGAGCTACAGTGCTTCATCTCA
miR-143	TGAGATGAAGCACTGTAGCTCA
M	CGAGTCAGAATGTCAGATACGAGAAGTCAG
O	GAGTCATGTGCGTCGTCTAAGCCTATCCTTTCCGAGCTACCATCACCAGTCTTACCTTTTCGATTCCAGTTCACAG
P	GGTAAGACTGGTGATGGGACGACGCACATGACTCCTGACTTCTCGTATCTGACATTCTG
M_{in}	CCTTCCTATCGCATTCTTTCTGACTTCTCGTATCTGACATTCTGACTCGTAGCTTATCA
C_{21}	TCAACATCAGTCTGATAAGCTACGAGTCAG
Control C_{21}	TCA**TT**ATCAGTCTGATAAGCTACGAGTCAG
miR-21	TAGCTTATCAGACTGATGTTGA
P_{in}	GTGCAGGTAGGTCAGGAGTCATGTGCGTCGTCCCATCACCAGTCTTACCTTTTCAGAGCTTGCTAGTCA
C_{17-3}	CTCCTGACCTACCTGCACTGTAAGCACTTTG

Table S1. Sequence of oligonucleotides used in modular logic units.

**Name**	**Sequence (5'-3')**
Control C_{17-3}	CTCCTGACCTACCTGCACTGTAAGCA**TC**TTG
Q	TAGCTCGGAAAGGATAGGCTTAGACGACGC
S	CATCGATTCGTCTGCCTTTTCGTCATTGCATCAGTGGTATCTGTCTAGAGCGTGAACAGTATGAC
R	GTCTAAGCCTATCCTTTCCGAGCTAGTCATACTGTTCACGCTCCACTGATGCAATGACG

R_{in}	CATAGCACTTACCGAGTTTCGTCATTGCATCAGTGGAGCGTGAACAGTATGACTAGCTTGTAAACATCC
C_{30a-4}	CTTCCAGTCGAGGATGTTTACAAGCTAGTC
Control C_{30a-4}	CTT**TT**AGTCGAGGATGTTTACAAGCTAGTC
T	BHQ2-GCGCACGCTCTAGACAGATACCACTTTTTAGTCCACACCAATCCG
Aso	GTCTAGAGCGTGCGCCAT-Cy5
M_{in}-staple(105)	GAATGCGATAGGAAGGTTTCATCATCGCACTCCAGCCAGCTTCGCTAT
P_{in}-staple(104)	TGGCAAGAGTCTGTCCATCACGTAGTAATATGACTAGCAAGCTCTG
O-staple(129)	GGAAGGGCCGCTCACAATTCCACGCCTGGGCTGTGAACTGGAATCG
S-staple(153)	GGCAGACGAATCGATGAATTAATTACAGTAACAGTACCTAATTCAT
R_{in}-staple(130)	GAGGGTAAGTGCTATGCAGGGCGCCAGGAACGGTACGCCAAGAACAAT
T-staple(178)	GGTTGAGGCTTTTCATAATCAAAAAGGCCGCGGATTGGTGTGGACT
U	FAM-TTTTTTACCATCCTATCCGACA
U-staple(184)	AAATACCGGGCAAATCAACAGTTGAGTATTAGTGTCGGATAGGATGGT
U-staple(185)	ACCCGTCGGGGGACGACGACAGTATGTGCTGCTGTCGGATAGGATGGT
U-staple(186)	AATAAGGCGTGTCGAAATCCGCGAACTCATCTTGTCGGATAGGATGGT
U-staple(187)	ATTTCAACAACCCTCGTTTACCATTTGCAATGTCGGATAGGATGGT
U-staple(188)	ATAAAAATTCAGAGCATAAAGCTAACAGTTGATGTCGGATAGGATGGT
U-staple(189)	GTGCCTAAGTTTGCCCCAGCAGGGGCAAAATGTCGGATAGGATGGT

Table S2. Sequence of oligonucleotides used to synthesize DNA origami.

Name	Sequence (5'-3')
1	AACATAAACTGAATCTTACCAACAATAGCA
2	ACCGTAATAATTCATATGGTTTACACATACAT
3	ATATATGTATCAATATGATATTCAAGACAGTC
4	GACGCTGACTGATGCAAATCCAATAGTATCAT
5	ATCCTTTGATTATCAGATGATGGCTTTACATC
6	CAGTTTGAGATTCTCCGTGGGAACAATCAGAA
7	CACCACACTTTTTATAATCAGTGAACTCAAAC
8	GAGGCTTTCATTAAACGGGTAAACCAACTT
9	TTTGAGTCAGAAGGAGCGGAATTGATTGCT
10	CTGCAACAATCTAAAGCATCACTTTAAAAG
11	CAAGAACACAAGCAAGCCGTTTCAGTTACA
12	GCCTGGCCTCACTGCCCGCTTTGAATTCGT
13	GATTAGCGGGGTTTTGCTCAGTACCAGGCGGA
14	AAAGGTGGAATTGAGTTAAGCCCAAGACGGGA
15	ATCGATGATGTACCCCGGTTGATAAACGGC
16	AACCTGTTGAGAGTACCTTTAATTAAAGACTT
17	ACCTCAAATGAGGCGGTCAGTATTTGGCAGAT
18	ATGCAATCAAAGAATTAGCAAATTTAGTTT
19	CAATATAAAACAATTCGACAACTAATATCT
20	GTGAGACGTCGTGCCAGCTGCATTTCGACTCT
21	TAGTAGTAGGTGAGAAAGGCCGGACCGTTC
22	ATAAAGCCTTTTAGAACCCTCATATATCAGGT
23	TATTCCTGCCCGAACGTTATTAATCTTGCTGA
24	GAGAAGTGCCGCCGCGCTTAATGCGCCGCTA
25	TTACCGAAATGATTAAGACTCCTTCGATTGAG
26	ATTACCGCCACACGACCAGTAATAGAAGATAA
27	ACGAGTAGAACGGAGATTTGTATCGCGCGAAA
28	CGCTGAGGCAGCGATTATACCAAATCGCCT
29	GAAAGCCGAAATCAAAAGAATAGCCAAGCGGT
30	TATCGGCCCCAACAGAGATAGAACGCCATTAA
31	CAACTAAAAATCTCCAAAAAAAAGGCTACA
32	TCACCAGTCAGCCATTGCAACAGGTTAAAGGG
33	TCTAAGAAAAGTCCTGAACAAGAAAAAGTAAT
34	CAAAAAGCTGGATAGCGTCCAAATAACGCC
35	GAGGCAATCGTCACCCTCAGCATATCAGCT
36	GGGTTGAGGCCCCCGATTTAGAGCTTGACGGG
37	GGCATTTCAAATAAATCCTCATTAAAGCCA
38	TAATCTTGAAGGGAACCGAACTGAATACGTAA
39	CAGGATTATAGCTATATTTTCATCTACTAA
40	GGGTAATTTAAATTGGGCTTGAGAACACCAGA
41	CCGACTTGTGCAGAACGCGCCTGTCAACATGT
42	ATATTTAGAGATCTACAAAGGCTATTTTAA
43	CCACAAGCAACATATAAAAGAATCACAATC
44	TGATAAGAAGATACATTTCGCAAAAATCATAC
45	TTCCCAATTGCTGAATATAATGCTTTACCCTG
46	AGAGGATCTAAGTTGGGTAACGCCGCATCTGC
47	ATATTTTTGCAATACTTCTTTGATCAAATTAA
48	CTGTTTGAAAGCATAAAGTGTAAAACAACATA
49	TGCCACTAGAACGAGGGTAGCAACGGCTCCAA
50	GAGCCGCCCCTCAGAGCCGCCACCAGAACC
51	AAGATTATGAACACCCTGAACAGAGATAAC
52	TTGACCCCCTTGCAGGGAGTTAAAACAGCTTG

Table S2. Sequence of oligonucleotides used to synthesize DNA origami.

Name	Sequence (5'-3')
53	AACAGAGGTATCAAACCCTCAATCCGTATTAA
54	ACAGTTTCTTTAATTGTATCGGTTGCGAAAGA
55	TGCTTTCGGGATTTTGCTAAACAACTTTCA
56	GCCATTCGTAACCAATAGGAACGCAAATCAGC

57	ACCACATTTGGGAAGAAAAATCTAATCAAGAG
58	AATAGAACAGTAGCGACAGAATTTTTCATC
59	GTCCACTACGTAAAGCACTAAATCGGAACCCT
60	ACCGCTTCGCGTCTGGCCTTCCTGATTTTGTT
61	GGTTGATATAAGTATAGCCCGGAATAGGTGTA
62	ACTTTACATCCTGATTGTTTGGATAACGTCAG
63	CCTTTAGCGAATAAGTTTATTTTGACGCAAAG
64	AAATGAAACCAGAGCCTAATTTGCTTATTTTC
65	CCGTTGTATGGTGGTTCCGAAATCCGAAAATC
66	AACATGTTGTTTCATTCCATATAAATCGGT
67	GAAACGTCAGGGCGACATTCAACATTACGC
68	GAATTAACGTTGCTATTTTGCACCTCCGGTAT
69	TCATTTTTTTTGCACGTAAAACAGTACCATAT
70	ATGCGTTAGCATTTTCGAGCCAGTTAGAAACC
71	AAAATTCGAAATTAATGCCGGAGAGGTAAAGA
72	ATACCGATTTTCCAGACGTTAGTAAATGAATT
73	AAAAGGAAAGAACTGGCTCATTCATTACCC
74	TCAGCTAAGAAATACCGACCGTGTCTAAATTT
75	ACCACCAGCCACCACCGGAACCGCGCAAAATC
76	AGTATGTTACAATGAAATAGCAAAGAGAAT
77	TTCAAAAGGCATTAACATCCAATATGGTCAAT
78	AAATTGCTAATGGAAACAGTACTTCTGTAA
79	AATAGATCGCGAGGCGTTTTAGCTTAAATC
80	CGAACCAGAAAATAGCGAGAGGCTGACGACGA
81	ATTTTAGAGTACTATGGTTGCTTTGACGAGCA
82	CAAATATCTTGCCAGAGGGGGTAAAGAGCAAC
83	GGATAGCAAGCCCAATAGGAACCCATGTACCGTAACACTG
84	AAATGCTTTCAAAAATCAGGTCTGTAGCTC
85	CAACGCTTTAACAACGCCAACATATCATTC
86	ATAGCCGCAATAATAACGGAATAATTATTC
87	AAAACGAATTCATCAGTTGAGATCCCCCTC
88	GACCATTGGTCATTTTTGCGGAGATTGCAT
89	TCCCTTATGCGAACGTGGCGAGAAAGGAAGG
90	AGGCAGAGTACAAATTCTTACCAGTAACTATA
91	GAAACCTGGGCAACAGCTGATTGCGAACAAGA
92	TGTTTAGAATTAAGAGGAAGCCCGGCTCCTTT
93	CAAACTACAACGCCTGTAGCATTCCACAGACA
94	CCACGCTGTGAGTGAGCTAACTCAGTGTGAAA
95	ACTATTATTAAATATTCATTGAATTTAGGAAT
96	AAAGGGATGTTGTTCCAGTTTGCCTTCACC
97	ATCGTCGATTTCATCTTCTGACGATAAATA
98	AAGAAGTTGCGTTTTAATTCGAGAACAGGT
99	TTGTCGTCAGTTGCGCCGACAATATTCGGT
100	TACGCCAGGTCATAGCTGTTTCCTCATTAATT
101	AACCAGAGAGCCGCCGCCAGCATTGACAGGA
102	GGATTGACGCGCATCGTAACCGTAGGGTTT
103	TTCTGTATGAGGTGAATTTCTTAAGGCCGCTT
104	TGGCAAGAGTCTGTCCATCACGTAGTAATA
105	TTTCATCATCGCACTCCAGCCAGCTTCGCTAT
106	ACCAGTAGGGTAAATATTGACGGAACCCAAAA
107	TGAGGAAGATAGCCCTAAAACATCCCTTCTGA
108	ATGTTACTCGTAACAAAGCTGCTCTACCTTAT
109	CGAGCTGAACCGGAAGCAAACTCCCTTCAAAG
110	CCATCGCCAGTTAGCGTAACGATCTAAAGTT
111	AAGCCCCAGAGAGTCTGGAGCAAAACGCAAGG
112	AGTAAAAGTGTAGCGGTCACGCTGCGCGTAAC
113	CATTGCCTAAAACAGGAAGATTGTGAGTAACA
114	CAGCATCGCGAAGGCACCAACCTAGCAGACGG
115	ATTTTTGAAATTGTAAACGTTAATTAGCCAGC
116	GCGTTGCGCCTGAGAGAGTTGCAGCCGAGATA
117	GAACTGGCGCCCTTTTTAAGAAAAAACGTCAA
118	TGAATAATCATTTGAGGATTTAGAAAAGGAAT
119	AAGACAAAACCAATGAAACCATCGTATTAGCG
120	ACACCACGGAGCGCTAATATCAGAAAGTCAGA
121	TCCCATCCAGATATAGAAGGCTTACAGCTACA
122	TTGAATATCATTTCAATTACCTATTTATCA
123	AATCATGCTGGCGAAAGGGGGATCGGCCTC
124	TGCCCCCTGCCTATTTCGGAACCTATTATTCT
125	CGAGCCGGCCATTCAGGCTGCGCAGGCAAAGC
126	ACATCACCGTAAGAATACGTGGTTAATGCG
127	TTGCGGGAAAGAATACACTAAAACCCTGCTCC
128	GCGTCTTTATAGCAGCCTTTACAGTAGCTATC

Table S2. Sequence of oligonucleotides used to synthesize DNA origami.

Name	Sequence (5'-3')
129	GGAAGGGCCGCTCACAATTCCACGCCTGGG
130	CAGGGCGCCAGGAACGGTACGCCAAGAACAAT
131	CTCTGAATTTACCGTTCCAGTAAGCGTCATAC
132	ACCTTTTTGTAGATTTTCAGGTTTTATACTTC

133	GCGATTTTATTACGAGGCATAGTATAGTAAAA
134	AATGGTTTGAGTGAATAACCTTGCATAAATCA
135	AAATCAATAGCCGGAACGAGGCAAACGAAA
136	ACTATCATTTTAATCATTGTGAATATTCAGTG
137	AATAATTCTGGTGCCGGAAACCAACTGTTG
138	CGGCCAACCAGGGTGGTTTTTCTCCAACGT
139	AATCAATAAATCATTACCGCGCCCGCTAACGA
140	GAATATAAAGAAAAAGCCTGTTTCGCAAGA
141	GGTCAGTTAACGAACCACCAGCAAAAGGGA
142	TGAAAGAGGGCTGGCTGACCTTCCGTTAAT
143	AGGCGTTGACGACGACAATAAATTATCAAC
144	TGTAAATGGAAGAGTCAATAGTGAGAGCAAAA
145	GATAAATTTTGCCCTGACGAGAATGGTTTA
146	TGATGATACAGGAGTGTACTGGTAATAAGTTT
147	CGTATAATAAACAGGAGGCCGAAAAAACGC
148	CATTCTGGTTGCTGGTAATATCCGAATCCT
149	TAGCTGATCATTAAATTTTTGTTCATCAAA
150	AAAGTATTAAGAGGCTGAGACTCCTCAAGAGA
151	ATTTTATCAACAGGGAAGCGCATTATAATAAG
152	TCTGTCCAAAATAAGAATAAACACAAATATAT
153	AATTAATTACAGTAACAGTACCTAATTCAT
154	GAAGAAAGCGAAAGGAGCGGGCGCTAGGGCGC
155	AGCTTAATTCTGCGAACGAGTAGAATTAAGCA
156	TCCCAGTCTTGCATGCCTGCAGGAATGAAT
157	CAAAGGGCACCCAAATCAAGTTTTTTGGGGT
158	CACTAACAGAATGGCTATTAGTCTCACAGACA
159	GGAGGGAACACCATTACCATTAGCATCACCGG
160	GTTTAGTACCGCCACCCTCAGAACCGCCACCCTCAGAACC
161	CGAGGTGCTTAAAGAACGTGGACTTTTCACCA
162	AAGGAGCCAGCGGAGTGAGAATAGAAAGGAA
163	CAAAGAACAACATAGCGATAGCTAAAACAA
164	ATCGTAGGATCGGCTGTCTTTCCTTGTAATTT
165	CGAACTGGTTATCTAAAATATCCGTCAATA
166	TTTGCCATCAGGTCAGACGATTGGCCTTGATA
167	CCTGAAAGTTGCCTGAGTAGAAGAGGCCACCG
168	AGCAAGAAAGCAAACGTAGAAAATCAGCGCCA
169	AATCGTCAAGTCAGAAGCAAAGCGTGGCTTAG
170	AGCAAATCTAATTTACGAGCATGAATAAGA
171	TAATTACTAGTACCGACAAAAGGTAAATAATA
172	TAAAAACCCGGGAGGTTTTGAAGCCGAACCTC
173	ATGAATATACATTTAACAATTTCACCTTAGAA
174	TCAATCATACAAGAACCGGATATTATACCAGT
175	AAGGCGATCCCGGGTACCGAGCTCCCAGTCGG
176	AGGCAAGGGCCTGAGTAATGTGTAGGGTAGCT
177	TCATGGAATGGATTATTTACATAACACCGC
178	GGTTGAGGCTTTTCATAATCAAAAAGGCCG
179	TCCTTGAAGCGAGAAAACTTTTTCCGGAATCA
180	TTCACAAATCGGTCATAGCCCCCTATAGCAGC
181	TCAGTGCCTTGAGTAACAGTGCCCGTATAAAC
182	GAAGATGACAATAACGGATTCGCCTATCATCA
183	GAATGGAAGTCAGACTGTAGCGCGCAAGTTTG
184	AAATACCGGGCAAATCAACAGTTGAGTATTAG
185	ACCCGTCGGGGGACGACGACAGTATGTGCTGC
186	AATAAGGCGTGTCGAAATCCGCGAACTCATCT
187	ATTTCAACAACCCTCGTTTACCATTTGCAA
188	ATAAAAATTCAGAGCATAAAGCTAACAGTTGA
189	GTGCCTAAGTTTGCCCCAGCAGGGGCAAAA
190	ATTAAAGTGGGAATTAGAGCCACTCCCTCA
191	AAATAAAAACGATTTTTTGTTTGTAAGCAG
192	GATAATAGGAAGGGTTAGAACCAAATAAAG
193	TTTAGTTACTATTAATTAATTTTCTTTGAATT
194	GGGAGAAATGAAACAAACATCAAGTAGATTAA
195	CAAAATTAACTAATAGATTAGAGCTTTAGGAG
196	TGTACCAAGAAGCCTTTATTTCACAAGAGA
197	AGGAAGAACATTAAATGTGAGCATAAGCAA
198	TTGTTATCGATCGGTGCGGGCCTCTTTCCGGC
199	CAGGACGTCAACTAATGCAGATACTACTGCGG
200	AAATCATTTAGGTTGGGTTATATATAAAGC
201	CAAAGTACCACGCATAACCGATATGACAACAA
202	AAATCACCAAAGGTGGCATCAATTTTGGGGCG
